# Rational design, synthesis of some quinolinone-Schiff bases/ pyridazino[4,5-c]quinolinones with potent anti-lung cancer and antituberculosis performance

**DOI:** 10.1038/s41598-025-22255-0

**Published:** 2025-11-04

**Authors:** Eslam A. Ghaith, Nedaa N. Elnaggar, Wafaa S. Hamama

**Affiliations:** Chemistry Department, Faculty of Science, Mansoura University, Mansoura, 35516 Egypt

**Keywords:** Quinolinone, Schiff-base, Pyridazine, Anti-lung, Antituberculosis, Molecular modeling, Biochemistry, Cancer, Chemical biology, Chemistry, Drug discovery

## Abstract

This study reports the synthesis of binary and fused polycyclic hybrids based on quinolinone *through* the reaction of 3-acetyl-4-hydroxy-1-phenylquinolinone (AHQ) **1** with different amine and hydrazine derivatives to give corresponding Schiff bases **2-6** and hydrazones **8,9a-c**. Whereas, formylation of hydrazone **9a**, afforded pyrazolylquinolinone carbaldehyde **10**. In addition, angular tricyclic systems **14-19** were synthesized from the reaction of hydrazone **8** with different aldehydes involving piperonal, chloroquinoline-3-carbaldehyde, chromene-3-carbaldehyde, ferrocenecarboxaldehyde, glyoxal and terephthalaldehyde. Whereas, the treatment of hydrazone **8** with different ketones afforded the corresponding *bis*-hydrazones **20**-**22** instead of the pyridazino skeletons. As well, all the synthesized hybrids were screened for their potential *in vitro* anti-lung cancer and antituberculosis activities (A549) and H37Rv strains, respectively. The molecular docking studies showed various strong π and hydrogen interactions with the newly synthesized quinolinone scaffolds. Interestingly, compounds **15**, **16**, and **19** manifested superior activity against both A549 and H37Rv. Interestingly, the most potent compound was **15** with an IC_50_ value of 10.38 µM and a MIC value of 6.25 µM. Conclusively, a density functional theory (DFT) calculation was studied.

**Supplementary Information:**

The online version contains supplementary material available at 10.1038/s41598-025-22255-0.

## Introduction

Cancer is one of the most worrisome diseases, with an estimated 10 million fatalities each year^1^ As it is a complicated disease, the increased incidence has a major impact on our health^2,3^ In particular, lung cancer (LC) is the leading disease both in rate and mortality during the recent decade as lung cancer is increasingly diagnosed. In addition, lung cancer is one of the most frequently diagnosed cancers in this decade, and is considered one of the top eight most prevalent and deadly cancers affecting humans over the past two decades, accounting for 12% of total cancer cases combined^4^ Whereas, non-small cell lung cancer (NSCLC) is the main subtype of lung cancer, causing majority^5^ Whereby, radiotherapy involving ionizing radiation (IR) is a primary treatment option for NSCLC, particularly in inoperable patients. Although radiotherapy has a high efficacy, the tumor is inherently radioresistant, resulting in radiotherapy failure. So, it is an intractable challenge frequently faced by clinicians and oncologists. Moreover, NSCLC patients continue to have a poor survival rate even with the use of optimized therapy modalities like adjuvant immunotherapy and concurrent chemotherapy^6^ Despite the availability of effective anti-cancer medications, drug resistance has grown to be a serious problem. This has made it necessary to find new, reasonably priced medications with much structural variation. So, medicinal chemists are increasingly focused on developing anticancer drugs that are more effective and have fewer side effects^7^ In addition, tuberculosis (TB) is another major public health problem worldwide caused by Mycobacterium tuberculosis (Mtb)^8,9^ The H37Rv strain of Mtb is the most commonly infectious strain globally, and its genome serves as the standard reference sequence for Mtb^10^ The World Health Organization (WHO) reports that it is the second leading cause of death from infectious diseases globally, following HIV, as approximately 25% of people worldwide suffer from TB^11^ Also, TB can be pulmonary (affecting the lungs) or extra-pulmonary (affecting other locations)^12^ There are numerous theories that explain how Mtb infection results in lung cancer through immune system suppression, DNA damage, and the generation of inflammation. The primary theory is that Mtb promotes lung cancer by causing chronic inflammation^13,14^ In addition, chronic inflammation from Pulmonary tuberculosis (TB) induces lung tissue scarring and genetic mutation and alterations, as well as induction of necrosis and apoptosis or TB reactivation, creating a pro-carcinogenic environment. Immune suppression during TB may impair tumor surveillance, increasing susceptibility to malignant cell growth. Residual fibrosis or granulomas post-TB recovery may also elevate long-term LC risk^15-17^

**Fig. 1:**
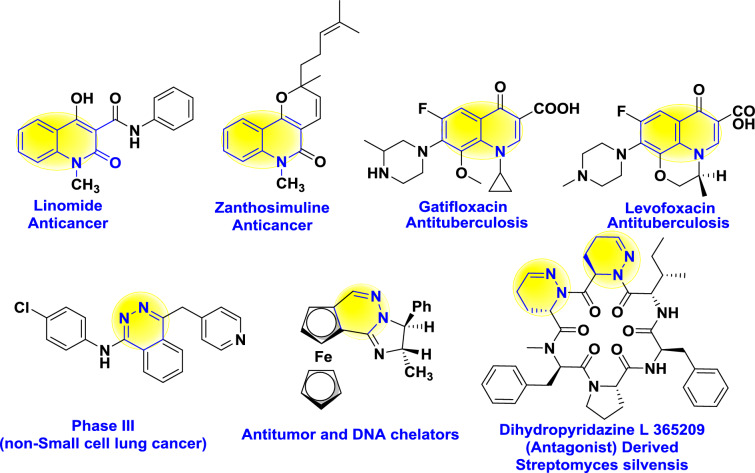
Natural and synthetic examples of bioactive candidates of quinolinone and pyridazine hybrids

Furthermore, nitrogenous heterocyclic hybrids play an important role in the design and manufacturing of chemotherapeutic medicines^18,19^ Quinolinone derivatives attract increased interest due to the stupendous chemical properties and their biological and pharmaceutical activity^20-22,23^, such as anticancer, antituberculosis (anti-TB), antiviral, antibacterial, antihypertensive, and others^24-25^, as shown in Figure 1. Moreover, many natural compounds, particularly alkaloids, include a quinoline ring, such as linomide, zanthosimuline, gatifloxacin, levofoxacin, identified as a potential lead bioactive precursor for developing anticancer and antituberculosis drugs^26,27,28^ On the other hand, pyridazine derivatives have a unique structural feature with characteristic electron-rich groups that readily bind with various receptors in biological systems *via* diverse interactions, exhibiting broad biological activities Figure 1.^29,30^ Whereas, pyridazine scaffolds possess inimitable physical properties, including an elevated dipolar moment, forming H-bond interactions due to the capability of two adjacent nitrogenous centers, facilitating salt formation; stimulating their industrial production^31^ Besides, pyridazines act as original functional surrogates of pyridine, carboxamide, and amines in many medicinal applications with more selectivity and a low toxicity ADME profile as a lipophilic drug candidate with bioisosteric consideration^29,30^

Because of the aspects mentioned above, herein new quinolinone derivatives were reported including Schiff bases, hydrazones, and polycyclic fused pyridazine, which have attracted continuous attention in the medical field due to their great anticancer profiles with the aid of molecular docking studies, Swiss ADME, and DFT as computational analysis tools.^32,35^

### Rational design

**Fig. 2:**
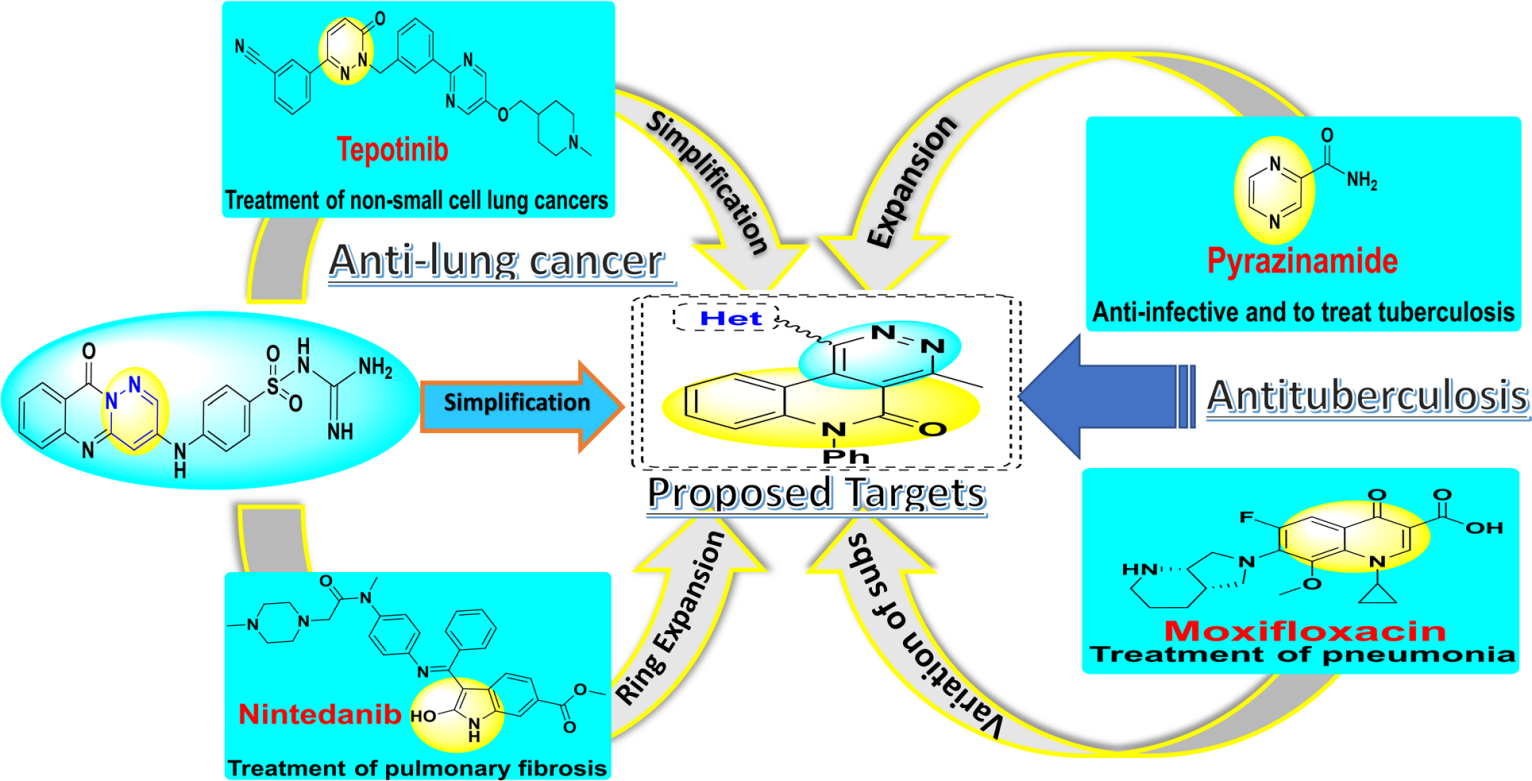
Rational design of fused pyridazino[4,5-c]quinolinone hybrids for the tested bioactivities.

Quinolinone Schiff bases and fused pyridazinoquinolinone derivatives represent canonical classes of anticancer and broad biological profiles. Herein, a panel of parent quinoline scaffolds clubbed with endowed nitrogenous entities involving amines, hydrazines, and pyridazines was designed and synthesized through direct methodologies. The established scaffolds inculpating different moieties such as benzene, benzo[*d*][1,3]dioxole, quinoline, chromone and ferrocene as bioactive hybrids for obtaining promising surrogates targeting anti-lung cancer and anti-tuberculosis through strategies for drug optimization Fig2.

## Results and discussion

### Synthesis and characterization

**Scheme. 1:**
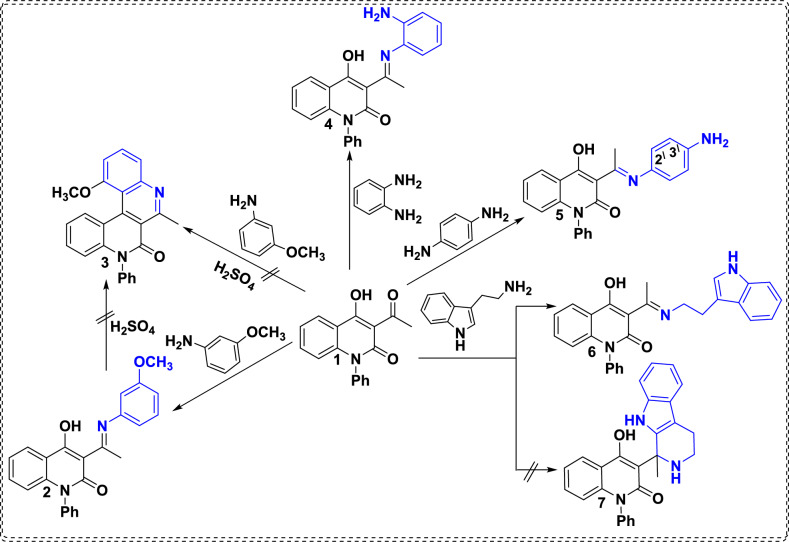
Reaction of compound** 1** with appropriate amine derivatives.

The condensation of 3-acetyl-4-hydroxy-1-phenylquinolin-2(1*H*)-one (AHQ) (**1**) with functionalized mono /di-nucleophilic amino derivatives such as *m*-anisidine, *o*-phenylenediamine (OPD), and *p*-phenylenediamine (PPD) afforded the corresponding Schiff bases **2**, **4**, and **5**, respectively (Scheme 1). Whereby, the ortho position for 3-methoxyphenylimino substrate of Schiff base **2** is strongly activated toward S_E_Ar, resulting in a preference for 6-membered ring cyclization with the pendant hydroxy group of quinolinone under acidic conditions. However, the attempts for formation of compound **3** was unsuccessful either direct or indirect reactions (Scheme 1). The chemical structures for the Schiff bases **2**, **4**, and **5** were elucidated by a combination of ^1^H, ^13^C NMR, mass spectrometry (MS), and IR techniques that are in accordance with their suggested structures. For constitution **2**, its IR spectrum displayed bands at 1640 and 3420 cm^-1^ attributed to amidic carbonyl and OH groups, respectively. Whereas, its ^1^H NMR spectrum shows two singlet signals at *δ* 2.64 and 3.78 ppm corresponding to the methyl and methoxy groups and a downfield signal at 16.53 ppm for the OH group. On the other hand, the IR spectrum of structure **4** reveals two bands at 3421 and 3336 cm^-1^ for the NH_2_ group besides the lactamic band at 1634 cm^-1^. Whereby, the ^1^H NMR spectrum for scaffold **4** revealed three singlet signals at* δ* 2.55, 5.31, and 15.82 ppm for CH_3_, NH_2_ and OH. Also, the MS spectrum of **4** showed a molecular ion peak at *m/z* 369.53, compatible with its molecular formula C_23_H_19_N_3_O_2_. Similarly, aiming to synthesize poly-functionalized *bis*-quinolinone *via* reaction of AHQ **1** with PPD furnished (*E*)-3-(1-((4-aminophenyl)imino)ethyl)-4-hydroxy-1-phenylquinolin-2(1*H*)-one **5** instead of the desired *bis* product. Its ^1^H NMR chart displays singlet signals at *δ* 2.63, 5.45, and 15.84 ppm for CH_3_, NH_2_ and OH groups, respectively. Interestingly, the inserted aromatic protons of PPD appeared as two doublet signals at *δ* 6.65 and 7.04 for C3^\^*-*C2^\^ with *J*_*coupling*_= 8.4 *Hz*.

The scope of this tandem route was extended by the reaction of AHQ** 1** with tryptamine at pH 4.7 (physiological conditions) according to the Pictet-Spengler reaction furnished (*E*)-3-(1-((2-(1*H*-indol-3-yl)ethyl)imino)ethyl)-4-hydroxy-1-phenylquinolin-2(1*H*)-one **6** instead of the desired product tetrahydro-*β*-carboline system **7**.^35^ Various spectral data established the molecular structure of **6**, as its ^1^H NMR spectrum revealed singlet signal at *δ* 2.65 ppm for methyl group, two signals at *δ* 3.22, 3.84 ppm characteristic for two methylene groups, singlet signal at 8.16 ppm characteristic for proton of indole and broad signal at *δ* 15.06 ppm for OH group. The ^13^C NMR spectrum displayed new signals at *δ* 25.4, and 44.6 ppm for two methylene groups. The mass spectrometry measurement of compound **6** shows a molecular ion peak at *m/z* 421.74, which confirms the recommended structure (Scheme 1).

**Scheme 2:**
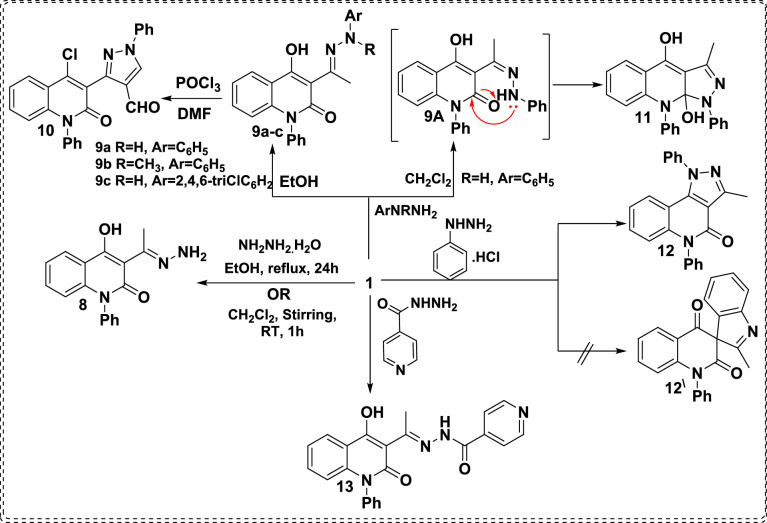
Reaction of acetyl compound** 1** with appropriate hydrazines.

The stupendous chemical reactivity of **1** toward binucleophiles was investigated through the treatment of AHQ **1** with hydrazine hydrate in EtOH or CH_2_Cl_2_ afforded (*E*)-3-(1-hydrazineylideneethyl)-4-hydroxy-1-phenylquinolin-2(1*H*)-one (**8**). It is worth mentioning that this reaction had previously been carried out in the presence of EtOH as the solvent; however, it required 24 hours to complete the reaction^28^ Therefore, a fundamental modification was made to the preparation methodology to obtain a pure sol product within 1h at ambient temperature by using CH_2_Cl_2_ as a weak polar solvent as an alternative solvent (Scheme 2).

The structure of compound **8** was elucidated by various spectral analyses, as its ^1^H NMR spectrum displays the presence of a singlet signal at 2.62 ppm characteristic of a methyl group alongside the presence of a characteristic singlet signal at *δ* 6.11 ppm attributed to NH_2_. The MS spectrum exhibits its molecular ion peak at *m/z* 293.28, which is attributable to the suggested structure.

In a similar manner, the chemical reactivity of **1** towards various arylhydrazines was implemented through the treatment of **1** with phenyhydrazine,^36^ 1-methyl-1-phenylhydrazine, and 2,4,6-trichlorophenylhydrazine in EtOH under stirring conditions at room temperature for 2h, furnished hydrazones **9a-c** (Scheme 2). The constitution of compound **9b** was confirmed by its ^1^H NMR spectrum, displaying the presence of two singlet signals of two methyl groups at *δ* 2.82 and 3.25 ppm and a singlet signal attributed to OH at *δ* 15.42 ppm. Also, ^13^C NMR spectrum assured the presence of signals at upfield *δ* 17.0 and 41.7 ppm for two methyl groups, besides downfield signals at *δ* 179.4, 177.4, and 163.5 attributed to (C-OH), (C=O, lactamic), and (C=N) groups. The mass spectrum confirms the constitution of scaffold **9b**, showing its molecular ion peak at *m/z* 383.54 as compatible with the suggested structure. The ^1^H NMR spectrum of hydrazone **9c** displays the appearance of three singlet signals at *δ* 2.80, 8.44, and 15.58 ppm characteristic of CH_3_, NH, and OH functionalities. Whereby, its IR spectrum showed two characteristic bands at ν^\^1640 and 3241 cm^-1^ related to the lactamic carbonyl and NH groups, respectively.

Due to the renowned biological activities of the quinolinone and pyrazole moieties, a combination of quinolinone and pyrazole in one molecular framework is one of the main targets for this manuscript. Subsequently, hydrazone **9a** was subjected to react with Vilsmeier-Haack reagent (DMF-POCl_3_) to afford binary pyrazoloquinolinone scaffold **10** (Scheme 2). The spectral analyses of compound **10** were in accordance with the suggested structure, as its ^1^H NMR shows singlet signals at 9.34 and 9.83 ppm characteristic of HC_Pyrazole_ and formyl protons, respectively. Whereas aromatic protons appeared as a multiplet at *δ* 7.55-7.65 ppm. On the other hand, its ^13^C spectrum displays a characteristic signal at *δ* 184.9 ppm corresponding to the formyl group, and upfield signals of aliphatic carbons are absent. Whereby, the mass spectrometry of scaffold **10** exhibited its molecular ion peak at *m/z* 425.14 and M^+^+2 at *m/z* 427.12 corresponding to chlorine isotopes, which is attributable to the suggested structure.

**Scheme 3:**
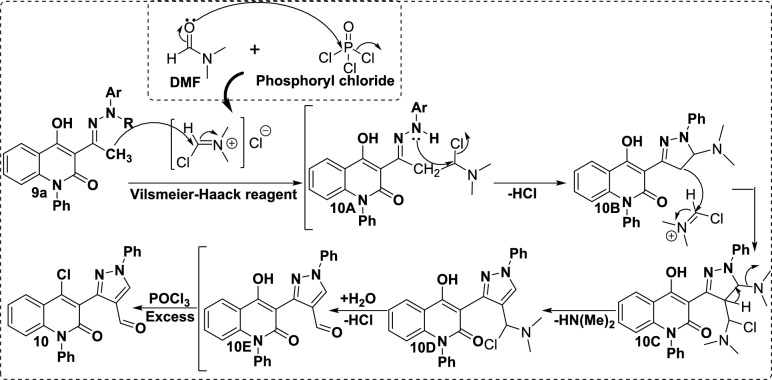
Plausible mechanism for formylation and chlorination of binary scaffold **10**.

The synthetic route for the binary quinolinone-pyrazole **10** is rationalized through an elegant mechanistic pathway that involves the generation of an iminium salt as a Vilsmeier-Haack reagent. Next, the terminal methyl electron-rich attacks the iminium salt, leading to intermediate **10A**. Whereby, nucleophilic attack of secondary NH functionality followed by cyclization and subsequent elimination of chloride ion to generate intermediate **10B**. Further, the intermediate **10B** encounters another iminium salt, which leads to the formation of intermediate **10C**. Then, the dimethylamine molecule is stripped off to yield intermediate **10D**. After that, the hydrolysis process of intermediate **10D** results in the formation of binary polycyclic formyl pyrazole intermediate **10E**. Finally, enolizable hydroxy proton is subjected to the chlorination process due to the excess effect of the POCl_3_ reagent afforded dihydroquinolin-1-phenyl-1*H*-pyrazole-4-carbaldehyde **10** (Scheme 3)^37,38^

Whereas, stirring of AHQ **1** with phenylhydrazine in CH_2_Cl_2_ at room temperature for 2h produced 3-methyl-1,9-diphenylpyrazolo[3,4-*b*]quinolinediol **11** instead of the anticipated Schiff base **9a** (Scheme 2). This reaction proceeds *via* a condensation reaction followed by nucleophilic attack of NH functionality to the electrophilic lactamic carbonyl group, followed by ring closure to yield fused pyrazolo[3,4-*b*]quinoline** 11**. The proposed structure **11** was elucidated by mass spectrometry measurement that showed a molecular ion peak at *m/z* 369.46. ^1^H NMR spectrum assured the presence of three singlet signals at *δ* 2.64, 9.55 and 16.23 ppm for the methyl group and two exchangeable OH groups.

Whereby, the difference in reactivity related to the utilized solvents was observed by Hamama *et al*.^39^ reported the chemoselectivity of the true carbonyl group through using a highly polar solvent (EtOH), whereas the use of a less polar solvent (CH_2_Cl_2_) led to the localization of the electropositivity on the other less reactive carbonyl group, and the viability of these postulations was supported by density functional theory (DFT) calculations.

**Fig. 3:**
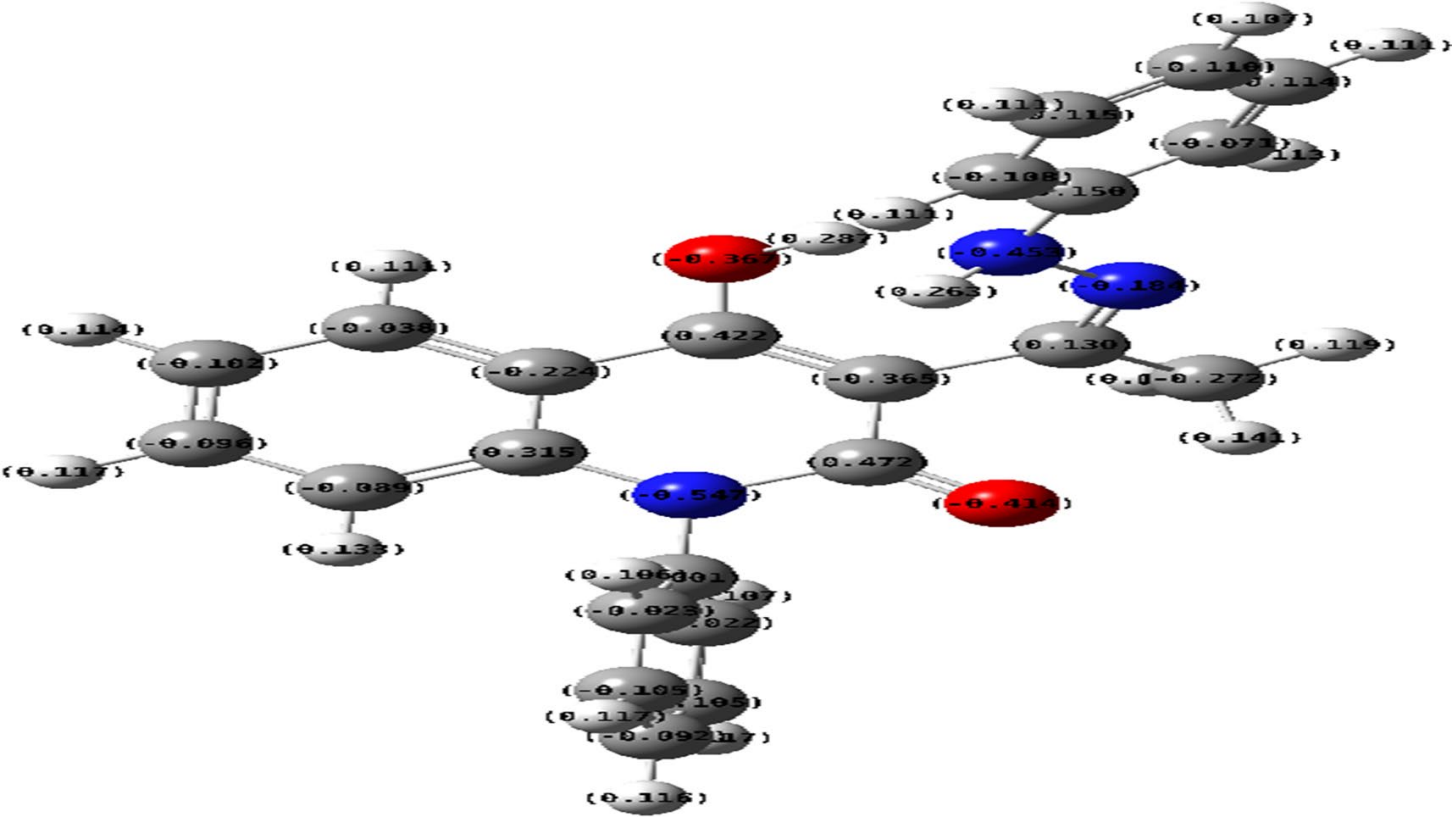
Electron density charge distribution of intermediate **9A**.

Analogously, the most acceptable explanation for formation of compound **11** was recognized through calculating the electron density values *via* density function studies (DFT) using BY3LP as function of charge on each atom, as the using of the CH_2_Cl_2_ as solvent (less polar solvent) alters the logic charge distributions of compound **11**, leading to accumulation of negative charge on NH functionality makes it more nucleophilic position (-0.453), whereby the most electropositive charge is allocated on the lactamic carbonyl group (+0.472) than other enolic carbon at position 4 (+0.422) as presented in figure 3.

Additionally, reaction of **1** with phenylhydrazine hydrochloride in a refluxing mixture solvent system of glacial acetic acid and concentrated hydrochloric acid (4:1) according to the Fischer indole synthesis procedures afforded 3-methyl-1,5-diphenyl-1,5-dihydro-4*H*-pyrazolo[4,3-*c*]quinolin-4-one (**12**)^36^ in 88% yield instead of the anticipated spiro Fischer indole product **12**^**\**^. The structure of compound **12** was confirmed by its ^1^H NMR spectrum, which displayed the presence of a singlet signal of methyl groups at *δ* 2.56 ppm. The ^13^C NMR spectrum showed a signal at *δ* 12.8 ppm for the methyl carbon. Whereas, the IR spectrum showed a broad band at 1667 cm^-1^ characteristic of lactamic group. The mass spectrum of this compound showed a molecular ion peak at *m/z* 351.61 (35.43) and 352.27 (M^+^+1, 26.97), confirming the proposed structure.

Isoniazid (INH) is a trademarked antibiotic used to treat both latent and active tuberculosis, besides demonstrating potential anti-proliferative actions. Till now, INH is still considered the first-line drug against TB due to its lesser toxicity, higher efficacy, and high aqua-solubility. Furthermore, it causes inhibition of the synthesis of mycolic acid as an essential component of the mycobacterial cell wall^40-42^ Based on the merits as mentioned above, and in continuation with our trials to synthesize and develop new anti-cancer agents, INH was utilized to react with AHQ **1**, affording 1,2-dihydroquinolin-3-yl(ethylidene)isonicotinohydrazide **13** with an unsuccessful trial of its formylation through the Vilsmeier–Haack reaction (Scheme 2). The constitution of hydrazone **13** was established based on IR, ^1^H NMR, and mass spectra. As its IR spectrum shows bands at 1615 and 1688 cm^-1^ corresponding to two carbonyl groups, and the appearance of a strong band at 3201 cm^-1^ is characteristic of NH. Also, its ^1^H NMR spectrum displays the presence of three singlet signals at *δ* 2.72, 11.96 and 16.88 ppm attributed to the methyl, NH and OH groups, respectively. Whereas the MS spectrum of compound** 13** exhibited its molecular ion peak at *m/z* 398.02, which confirms the suggested structure.

**Scheme. 4:**
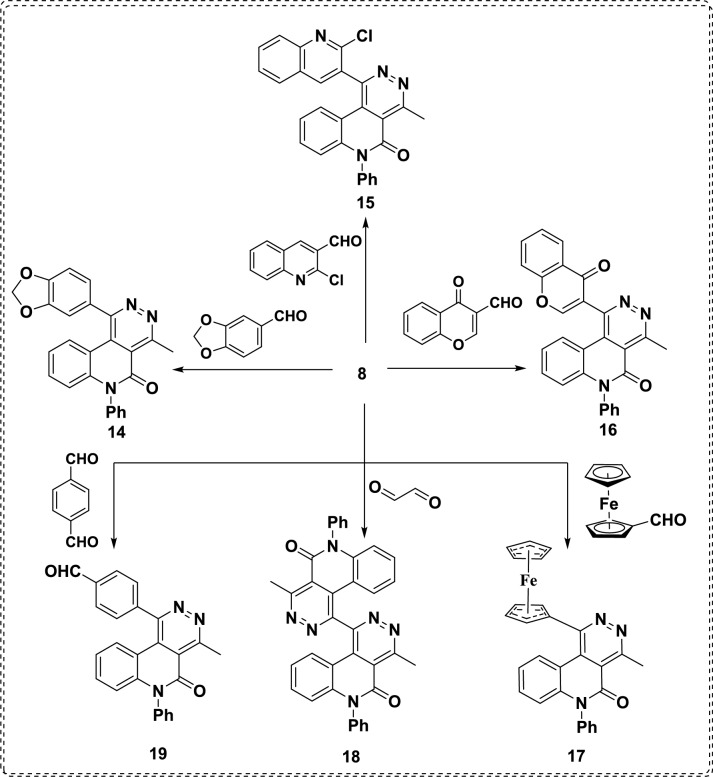
Reaction of hydrazone **8** with various aldehyde derivatives gave pyridazino derivatives **14-19.**

Pyridazine derivatives are aromatic heterocyclic compounds belonging to the diazine

family, characterized by nitrogen atoms at the 1,2-positions. Also, pyridazine can be utilized as a bioisostere for benzene or pyridine. Compared to other diazines, pyridazines exhibit higher pKa values (indicating greater basicity) and higher dipole moments. Besides, pyridazines have high potential for coordination with metals and forming hydrogen bonding, as well as their polarity increases due to the presence of additional nitrogenous atoms, leading to the formation of more water-soluble salts or crystals^43^ Thus, the reaction of hydrazone derivative** 8** with aldehydes seemed to be a unique route for synthesizing promising annulated pyridazinoquinolinone systems according to ring closure Baldwin’s rules *via* 6-*exo*-*trig* cyclization (Scheme 4).

**Scheme. 5:**
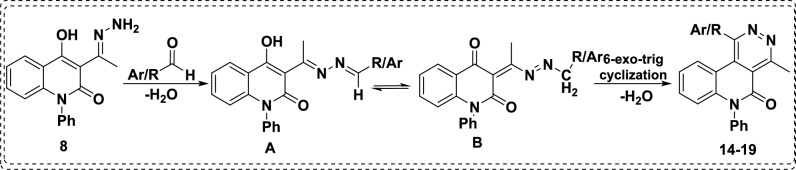
Mechanistic pathway for the reaction of hydrazone **8** with aldehydes, affording pyridazino derivatives.

The mechanistic pathway of the reaction of skeleton **8** and various aldehydes is described in Scheme 5, starting with a condensation reaction giving dihydrazone intermediate that isomerizes to ketoform, followed by 6-*exo*-*trig* cyclization yielding six new-fangled pyridazine entities **14-19** (Scheme 5). Consequently, treatment of hydrazone **8** with piperonal yielded 1-(benzo[*d*][1,3]dioxol-5-yl)-4-methyl-6-phenylpyridazino[4,5-*c*]quinolin-5(6*H*)-one (**14**) that was elucidated by different spectroscopic analyses, which were consistent with its proposed structure. As its ^1^H NMR spectrum demonstrates a singlet signal of the methyl protons at *δ* 3.10 ppm, it also shows a singlet signal at *δ* 6.04 for the methylene group of the piperonal moiety and has demonstrated the disappearance of NH_2_ and OH protons related to the parent structure of constitution **8**. Whereby, its IR spectrum shows a band at 1643 cm^-1^ characteristic of the lactamic C=O group. The mass spectrometry showed a molecular ion peak at *m/z* 407.93 (M^+^, 18.39) corresponding to a molecular formula C_25_H_17_N_3_O_3_.

Similarly, cyclocondensation reactions of hydrazone **8** with 2-chloroquinoline-3-carbaldehyde, 4-oxo-4*H*-chromene-3-carbaldehyde, and ferrocenecarboxaldehyde were carried out affording pyridazino[4,5-*c*]quinolin-5(6*H*)-one derivatives **15**-**17**, respectively. As the IR spectra of products **15**-**17** show the presence of characteristic lactamic peaks at 1625-1643 cm^-1^ due to highly conjugated systems.

The ^1^H NMR spectrum of compound **16** reveals a singlet signal at* δ* 3.00 ppm for CH_3_ and a sharp singlet signal at *δ* 9.07 ppm for the olefinic proton of the chromone moiety. Its ^13^C NMR spectrum demonstrates deshield signals at *δ* 174.8 and 158.0 ppm corresponding to carbonyl groups of C=O_γ-chromone_ and lactamic groups. Whereas, ^1^H NMR spectrum for** 17** gives singlet signals at *δ* 4.29, 4.58, and 4.75 ppm characteristic for protons of ferrocene moiety, the MS spectrum of **17** shows a molecular ion peak at *m/z* 471.92 (M^+^, 27.34) coinciding with a molecular formula C_28_H_21_FeN_3_O.

In the course of this study, the synthesis of *bis*-pyridazino[4,5-*c*]quinoline entities was investigated through the reaction of hydrazone **8** with glyoxal and terephthaldehyde as dialdehyde constitutions afforded the *bis*-cyclized product bipyridazino[4,5-*c*]quinolin]-5,5'(1*H,*6'*H*)-dione **18** and 6-phenyl-5,6-dihydropyridazino[4,5-*c*]quinolin-1-ylbenzaldehyde **19**, respectively. These structures are proven by various elemental analyses and spectral data. As the ^1^H NMR spectrum of **18** reveals a singlet signal at *δ* = 3.03 ppm for two symmetrical methyl groups and the absence of any signal related to formyl groups, confirming that the condensation reaction occurred at the two formyl groups of glyoxal. Moreover, ^13^C NMR spectrum showed signals at* δ* 181.4 and 174.6 ppm for two lactamic C=O, and showed two signals at *δ* 17.6 and 17.4 ppm for two methyl groups. Finally, the mass spectrometry measurement of **18** shows a molecular ion peak at *m/z* 572.00 (M^+^, 14.50), confirming the proposed structure. On the other hand, the ^1^H NMR spectrum of compound **19** demonstrates two singlet signals at *δ* 3.15 and 10.07 ppm attributed to methyl protons and the formyl proton. Meanwhile, characteristic signals were seen in the ^13^C-NMR spectrum for **19** at *δ* 191.4 and 180.9 ppm corresponding to two carbonyl groups. Whereas, the MS spectrum provides robust evidence to the suggested constitution displaying a molecular ion peak at m/z 391.65 (M^+^, 27.61) and a base peak at *m/z* 63.20, verifying a molecular formula C_25_H_17_N_3_O_2_.

**Scheme. 6:**
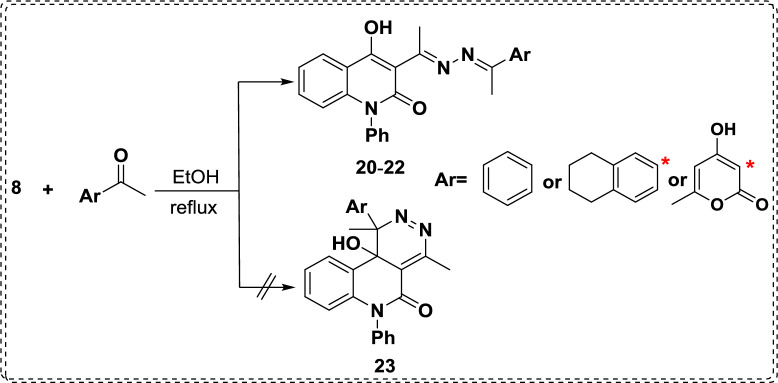
Reaction of hydrazone **8** with various ketonic derivatives.

The success of the cascade cyclization methodology encouraged us to repeat this reaction using a ketonic scaffold, aiming at extending the scope of our strategy through treatment of hydrazone **8** with different ketonic compounds, which afforded the opened condensation products **20-22**, not the cyclized one **23**. Thus, the reaction of **8** with acetophenone, 6-acetyltetralin and DHA yielded dihydrazone **20**-**22** (Scheme 6). The structures of hydrazones **20-22** were unambiguously elucidated using various spectroscopic analyses. As ^1^H NMR spectrum for **20** demonstrated two singlet signals of the two methyl protons at *δ* 2.63 and 3.14 ppm, its ^13^C NMR spectrum supports the suggested structure as recording sixteen signals corresponding to the diverse carbon atoms of the molecular formula C_25_H_21_N_3_O_2_. In addition, the EI-MS spectrum revealed its molecular ion peak at *m/z* 395.10, which was in accordance with its molecular formula.

Whereby, EI-MS reinforced the constitution of compound **21** due to the appearance of its molecular ion peak at *m/z* 449.28 (M^+^, 16.18), which was compatible with its constitution. Also, ^1^H NMR spectrum of designated **21** confirms the presence of upfield signals in the range of *δ* 1.75 and 2.78 ppm related to aliphatic protons of the tetrahydronaphthalenyl moiety. Whereas, ^1^H NMR spectrum of **22** shows three singlet signals at *δ* 2.22, 2.88, and 2.95 ppm related to three methyl groups, alongside the presence of two exchangeable singlet signals at *δ* 16.07 and 17.30 ppm characteristic for two hydroxyl groups, whereby their carbons resonated at *δ* 182.3 and 164.4 ppm in ^13^C NMR analysis. Furthermore, the IR spectrum showed two characteristic carbonyl signals related to lactonic and lactamic carbonyl at 1717 and 1644 cm^-1^, respectively. Its mass spectrum revealed a peak at *m/z* 443.39 corresponding to its molecular ion.

**Fig. 4:**
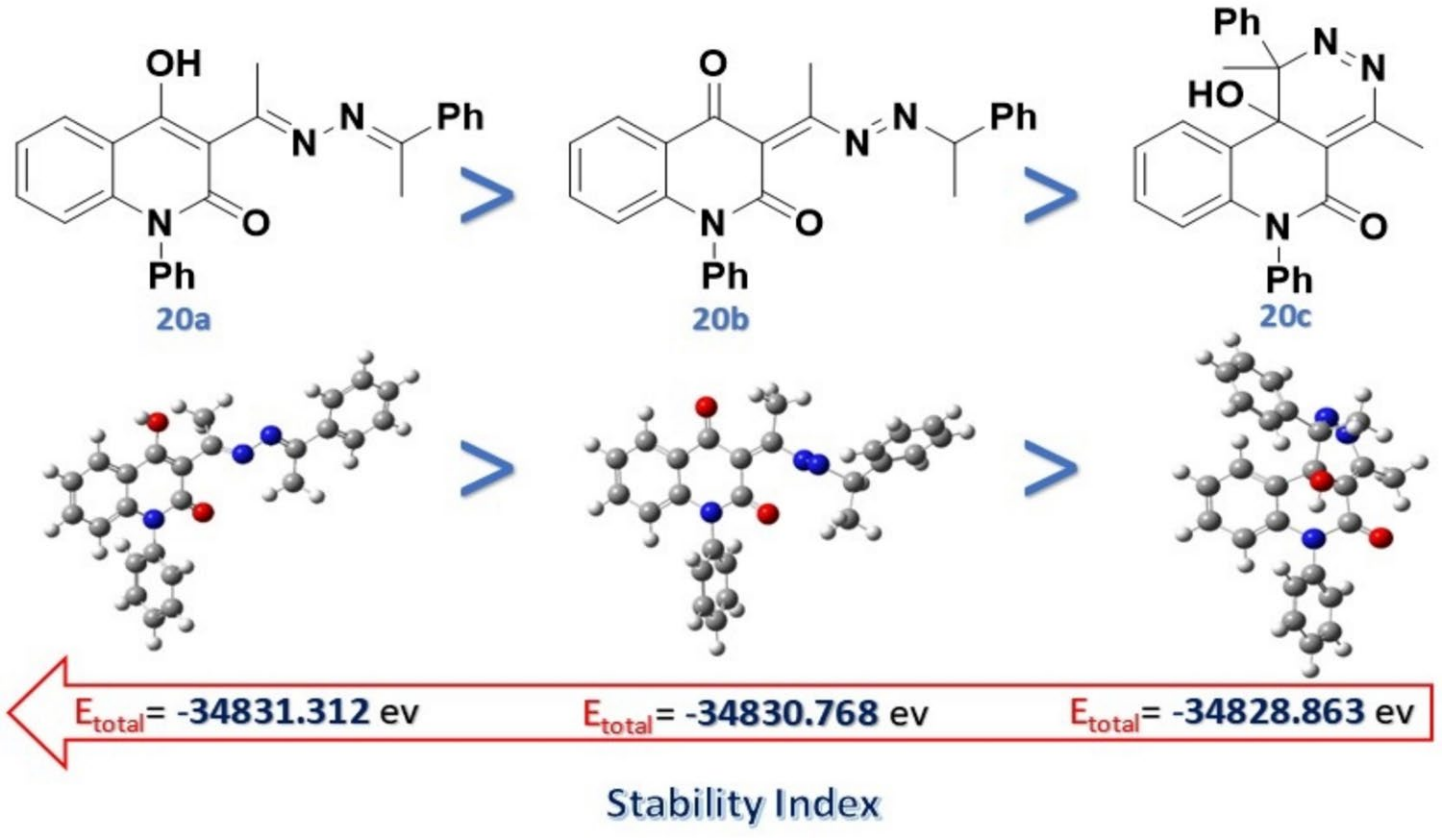
Total energies of the suggested geometrical product **20**.

According to Baldwin’s rules, an undesired (slow) reaction does not have a rate capable of competing effectively with the preferred (fast) alternative reaction. Additionally, DFT studies confirmed the obtained product **20a**, which predominates over the others (**20b**,**20c**) due to chemical stability (lower total energy, -34831.312 eV) than the others (-34820.768 and -34828.863 eV, Fig. 4.

### Biological evaluation

All the target compounds were screened for their potential *in vitro* anticancer activity against the lung cell strain (A549 cell line, which was obtained from VACSERA, Cairo, Egypt) by MTT assay. Also, they were tested for their potential *in vitro* antituberculosis activity against the Mtb H37Rv strain by Microplate Alamar Blue Assay (MABA).

#### In vitro anticancer screening

**Fig. 5:**
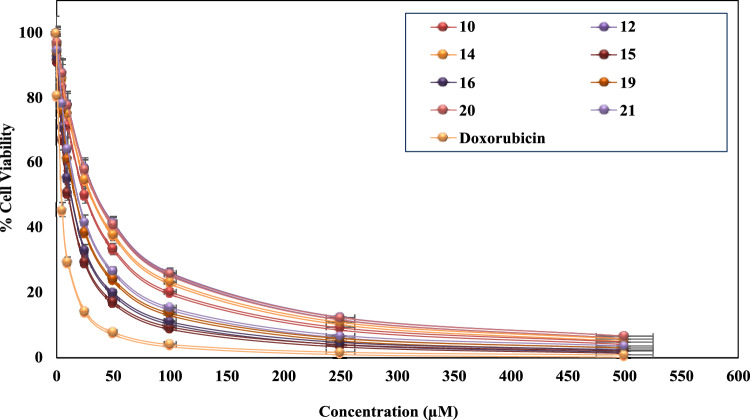
Cell viability curves of the most potent synthesized compounds for their anticancer (A549 cell line).

Initially, all of the newly synthesized quinolinone derivatives and the starting materials were examined *in vitro* to quantify their inhibitory activity against the anticancer (A549 cell line). Anticancer was performed using the MTT (Thiazolyl Blue Tetrazolium Bromide) method^44,45^ Doxorubicin, one of the common anti-cancer drugs, was employed for comparison. The concentration of compounds needed to inhibit the growth of 50% of cancer cells was expressed as (μM) and displayed in Table 1. The compounds under examination demonstrated varying degrees of inhibitory effects on the tested human tumor cells. All synthesized compounds and the starting material were evaluated for their efficacy in inhibiting lung cancer compared to Doxorubicin (IC_50_= 4.18 µM). The inhibition potency of the synthesized compounds was in the range of 10.38–over 100 µM. Compound **15** exhibited remarkable activity with an IC_50_ value of 10.38 µM. Further, the compounds **10**, **14**, **16**, **19**, and **21**, are proven to be of distinguished activity with IC_50_ values of 25.23, 30.55, 12.40, 15.91, and 18.01 µM, respectively. On the other hand, compounds **9b**, **9c**, **12**, **17**, **20** and **21** demonstrated moderate activity with IC_50_ values of 52.32, 57.62, 35.31, 54.12, 34.54 and 59.52 µM, respectively. The derivatives **2**, **4**, **5**, **6**, **8** and **11** recorded weak values of IC_50_ 71.54, 77.33, 80.23, 73.65, 92.22 and 93.23 µM, respectively. Finally, compounds **1**, **13**, and **18** showed no acceptable growth inhibitor effect (IC_50_ >100) (Table 1). Pyridazino[4,5-c]quinoline derivatives (**14**-**19**) demonstrated the highest activity with IC_50s_ in the 10.38-30.55µM range. Figure 5 shows the A549 cell line viability that is highly sensitive to compounds **15**, **16**, and **19** in the presence of varying concentrations.

**Table 1:** *In vitro* cytotoxicity of the samples against the A549 cell line. Data are presented as mean ± standard deviation (SD) from three independent experiments (n = 3).

Compound	In vitro Cytotoxicity IC_50_ (µM)	Compound	In vitro Cytotoxicity IC_50_ (µM)
**1**	>100	**13**	>100
**2**	71.54±0.12	**14**	30.55±0.39
**4**	77.33±0.21	**15**	10.38±0.22
**5**	80.23±0.17	**16**	12.40±0.08
**6**	73.65±0.35	**17**	54.12±0.27
**8**	92.22±0.62	**18**	>100
**9b**	52.32±0.42	**19**	15.91±0.13
**9c**	57.62±0.38	**20**	34.54±0.09
**10**	25.23±0.18	**21**	18.01±0.11
**11**	93.23±0.52	**22**	59.52±0.16
**12**	35.31±0.48	Doxorubicin	4.18±0.08

Significant values are in underline.

#### *In vitro* antituberculosis screening

Next, all the newly synthesized compounds were initially screened for their *in vitro* tuberculosis activity at a concentration of 3.125 mg/mL against Mtb H37Rv strain using the MABA. Isoniazid, one of the common antituberculosis drugs, was employed for comparison with compounds. The results of the antitubercular studies are presented in Table 2. The compounds under examination demonstrated varying degrees of inhibitory effects on Mtb (H37Rv) strain. All synthesized compounds and the starting material were evaluated for their efficacy in inhibiting Mtb (H37Rv) strain in comparison to Isoniazid (MIC= 3.12 µM). The inhibition potency of the synthesized compounds was in the range of 6.25–32.26 µM. Compounds **15** and **14** exhibited remarkable activities with an MIC value of 6.25 and 8.37 µM, respectively. Further, the compounds **6**, **16**, **19** and **21** are proven to be of high activity with MIC values of 18.58, 11.34, 12.25 and 14.72 µM, respectively. On the other hand, compounds **4**, **5**, **9b**, **9c**, **10**, **11**, **12**, **13**, **20**, and **22** demonstrated moderate activity with MIC values ≤30 µM. The derivatives **2**, **8**, and **18** recorded values of MIC >30 µM. Finally, compound **1** (starting material) showed the lowest value of MIC (Table 2).

**Table 2:** *In vitro* antitubercular activity of the samples against the *M. tuberculosis* H37Rv strain. Data are expressed as mean ± standard deviation (SD) from three independent experiments (n = 3).

Compound	MIC^a^ (μg/mL) ± SD	Compound	MIC^a^ (μg/mL) ±SD
**1**	42 ± 0.12	**13**	22 ± 0.07
**2**	32.26 ± 0.09	**14**	8.37 ± 0.11
**4**	26 ± 0.21	**15**	6.25 ± 0.16
**5**	25 ± 0.25	**16**	11.34 ± 0.22
**6**	18.58 ± 0.32	**17**	19 ± 0.52
**8**	37 ± 0.15	**18**	38 ± 0.62
**9b**	28 ± 0.17	**19**	12.25 ± 0.24
**9c**	26 ± 0.27	**20**	22.12 ± 0.13
**10**	23.52 ± 0.23	**21**	14.72 ± 0.18
**11**	20.82 ± 0.36	**22**	29 ± 0.31
**12**	27.53 ± 0.39	Isoniazid	3.12 ± 0.09

^a^Minimum inhibitory concentration against H37Rv strain of Mtb (μg/mL).

Significant values are in underline.

### Molecular simulations

Major efforts have been devoted to improving the algorithm for docking predictions, as molecular docking is an essential tool for rational medication design in the disciplines of biology and pharmacology. In light of that, molecular simulations can be used to analyze the potential interactions between produced drugs and protein receptors, providing vital insights into their binding patterns and potential antituberculosis and anticancer activity^46,47^ This analysis was intended to determine the efficacy of the synthesized scaffolds. The drug–target interactions were examined using molecular docking analysis to clarify the binding sites and to understand the mode of action for the high-potential synthesized candidates. Compounds **6**, **10**, **14**, **15**, **16**, **19**, **20**, and **21** were selected based on their high biological activity, indicating higher binding energies and higher therapeutic efficacy. The highest binding energy conformation was considered the most favorable docking pose. The binding energies of these compounds are presented in Tables 3 and 5. The components are as follows: (1) Final Intermolecular Energy, including Van der Waals forces, hydrogen bonds, and desolvation energy; (2) Final Total Internal Energy; (3) Torsional Free Energy; and (4) Energy of the Unbound System. The formula [(1) + (2) + (3) - (4)] was used to calculate the binding energies for each derivative in kcal/mol. Moreover, the K_i_ value or the inhibition constant is in fact the dissociation constant of the docked enzyme inhibitor complex. The smaller the value of K_i_, the lower will be the probability of dissociation, and hence higher will be the inhibition. It is calculated as Ki = exp(ΔG/(R*T)) where ΔG is the free energy of binding, R is the gas constant (1.987 cal *K*^−1^ mol^−1^), and T is the temperature (298.15 K).

**Table 3:** Docking results in terms of binding energy of the most potent synthesized compounds against 1M17 (anticancer application).

Compound	Binding energy (kcal/mol)	Inhibition constant (K_i_) (µM)	Final Intermolecular Energy (kcal/mol)	Final Total Internal Energy (kcal/mol)	Torsional Free Energy (kcal/mol)	Unbound System’s Energy (kcal/mol)
**2**	-7.21	5.23	-8.70	0.00	1.49	0.00
**6**	-8.36	0.75	-9.25	0.00	0.89	0.00
**10**	-9.08	0.22	-10.28	0.00	1.19	0.00
**12**	-7.94	1.51	-8.24	0.00	0.30	0.00
**14**	-8.84	0.33	-9.44	0.00	0.60	0.00
**15**	-9.97	0.05	-10.57	0.00	0.60	0.00
**16**	-9.87	0.06	-10.47	0.00	0.60	0.00
**19**	-9.59	0.09	-10.48	0.00	0.89	0.00
**20**	-8.08	1.19	-9.57	0.00	1.49	0.00
**21**	-9.37	0.13	-10.87	0.00	1.49	0.00

Significant values are in underline.

**Fig. 6:**
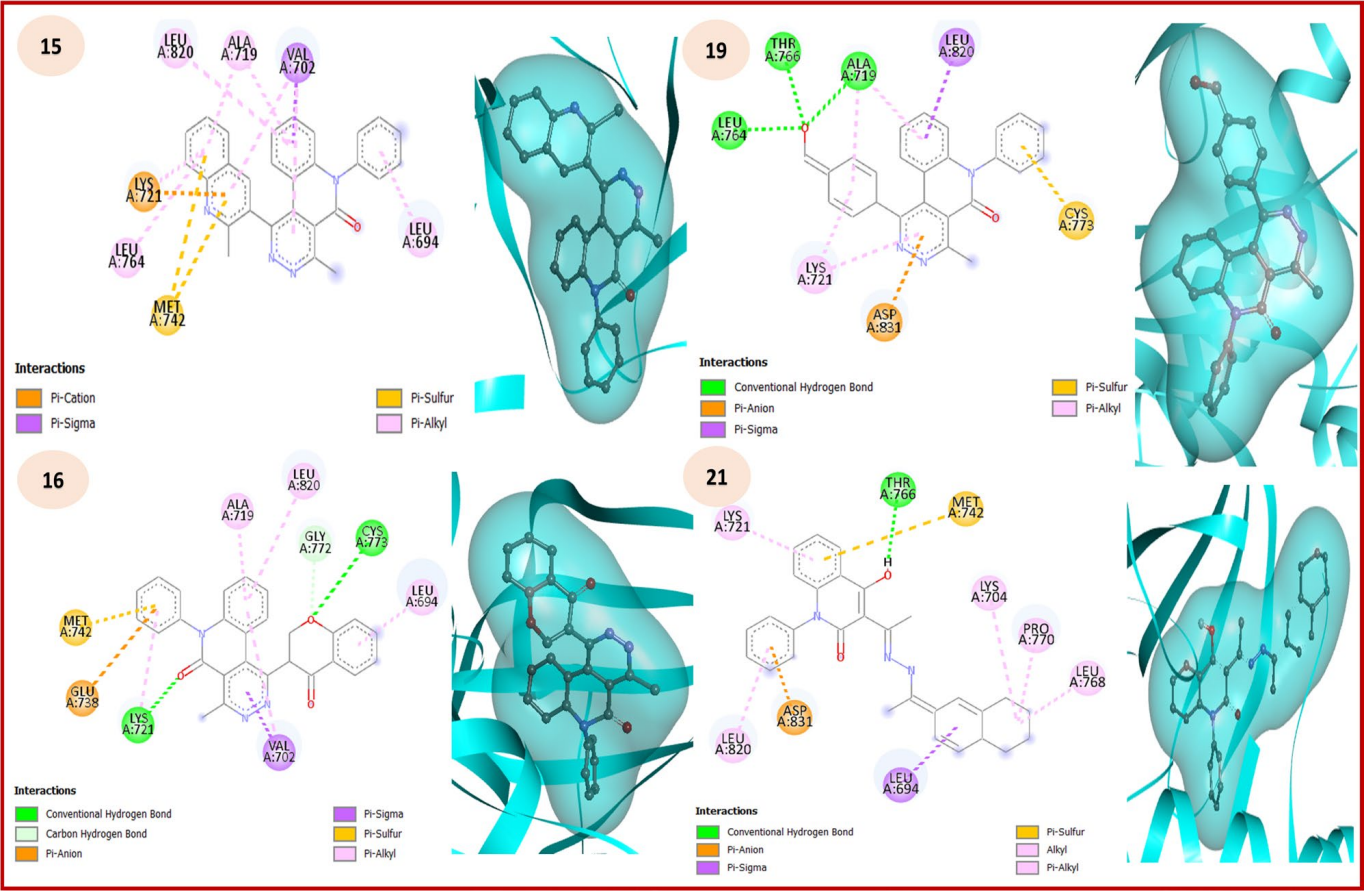
2D and 3D interaction plots of the high potent quinolinone ligands with **1M17** active site.

Whereas, epidermal growth factor receptors (EGFRs) are transmembrane receptors present on cell membranes, represent an effective role in controlling cellular functions involving cell growth, cell proliferation and apoptosis. In addition, the mutations of EGFRs can lead to abnormal stimulation of the receptors causing unregulated cell division, causing cancer such as NSCLC^48^ Whiles, 1M17 is a reprehensive protein code for EGFR tyrosine kinase domain as a crucial element in cell signaling related to growth and proliferation as a well-established anticancer inhibitor^49,50^ On observing the docking score of the most bioactive compounds with 1M17, it was observed that **15** displayed the higher docking score (-9.97 kcal/mol) followed by **16**, **19**, **21**, **10** and **14** with binding energy -9.89, -9.59, -9.37, -9.08 and -8.84 kcal/mol (decreasing order), compounds with higher docking score are pyridazino[4,5-*c*]quinolinone hybrids due their biological activity as illustrated in Table 3. The 2D and 3D docking interaction pattern revealed that essential interactions were present in all docked synthesized compounds, as it is clearly visible from Figure 6 and Table 4.

**Table 4:** Interactions of the most potent (highly active) synthesized compounds with 1M17.

No.	Interaction type	Amino acid residues	Distance for key Hits (Å)	No.	Interaction type	Amino acid residues	Distance for key Hits (Å)
10	*π*-sigma	GLY695	3.82	16	H.B	CYS773	2.91
	*π*-sigma	VAL702	3.08		*π*-anion	GLU738	4.81
	*π*-sulfur	MET742	5.64		*π*-sigma	VAL702	2.45
	*π*-alkyl	LYS721	4.05		*π*-sulfur	MET742	4.63
	*π*-alkyl	LEU764	5.44		*π*-alkyl	VAL702	4.77
14	*π*-anion	ASP831	4.86	19	H.B	ALA719	2.61
	*π*-sigma	VAL702	3.76		*π*-anion	ASP831	3.45
	*π*-sigma	VAL702	3.61		*π*-sigma	LEU820	3.99
	*π*-sulfur	CYS773	5.56		*π*-sulfur	CYS773	5.10
	*π*-alkyl	ALA719	4.29		*π*-alkyl	ALA719	5.17
15	*π*-sulfur	MET742	5.76	21	H.B	THR766	2.31
	*π*-alkyl	ALA719	4.29		*π*-anion	ASP831	2.69
	*π*-alkyl	LEU820	5.03		*π*-sigma	LEU694	3.92
	*π*-alkyl	ALA719	4.55		*π*-sulfur	MET742	5.90
	*π*-alkyl	LEU764	5.24		*π*-alkyl	LEU820	4.99

**Fig. 7:**
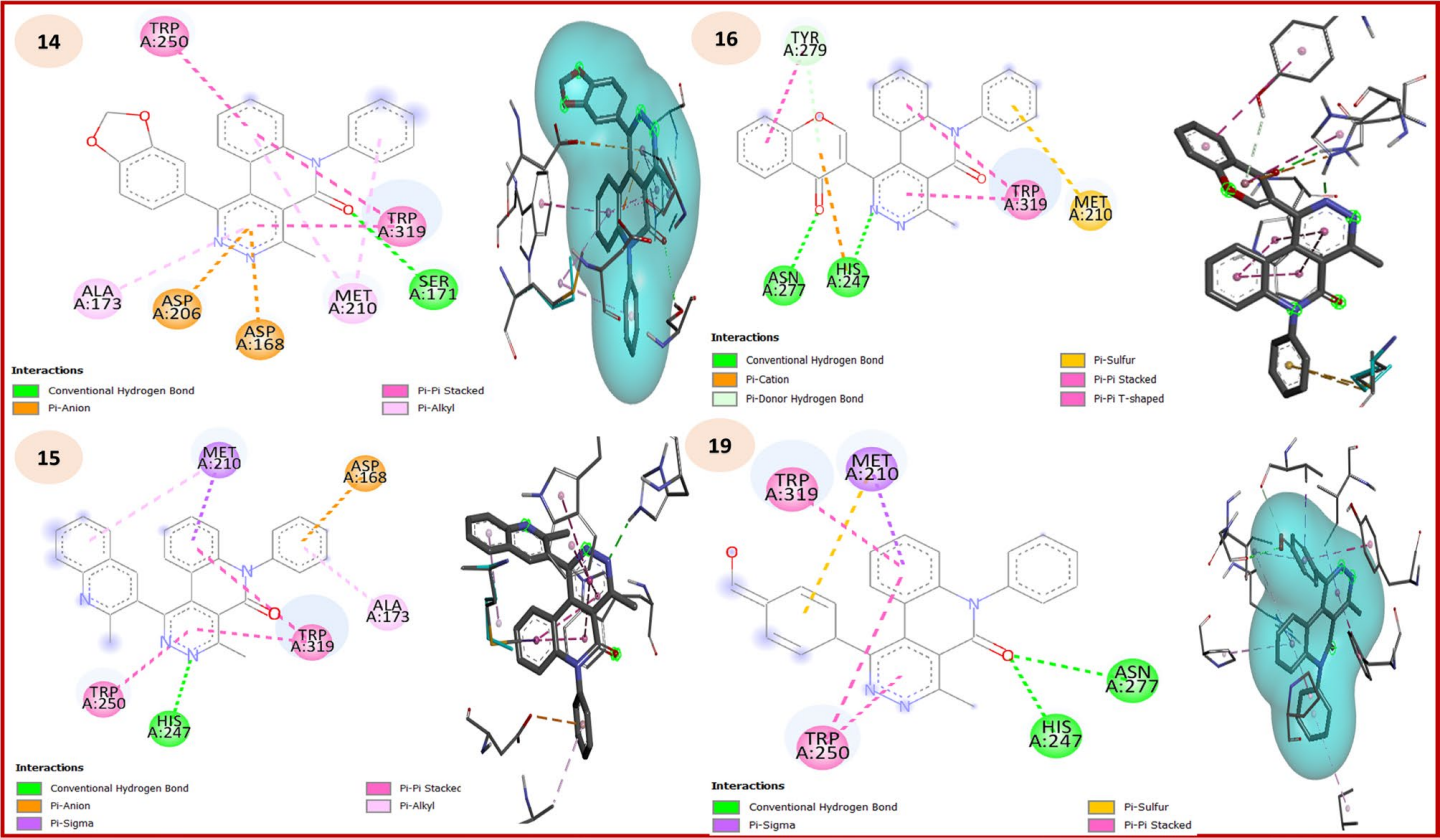
2D and 3D interaction plots of the high potent quinolinone ligands with **1OUZ** active site**.**

On the other hand, 1OUZ is a vital code for the InhA protein which plays a crucial role in the development of antitubercular drugs, as it is an effective carrier protein reductase from Mycobacterium tuberculosis, so inhibiting process for this enzyme prevents the bacterium from synthesizing its cell wall that is essential for the pathogen’s viability and survival.^51,52^ The docking score of the highly active compounds with 1OUZ (M.tb protein) supported the biological activity of the synthesized compounds. Also, it was observed that **15** displayed the highest docking score (-10.59 kcal/mol) followed by **14**, **16**, **19**, **21** and **6** with binding energies of -10.12, -10.05, -9.79, -9.51 and -9.41 kcal/mol (decreasing order) (Table 5). The 2D docking interaction pattern revealed that essential interactions were present in all docked synthesized compounds. Moreover, it is clearly visible from Figure 7 and Table 6.

**Table 5:** Docking results in terms of binding energy of the most potent synthesized compounds against 1OUZ (antituberculosis application).

Compound	Binding energy (kcal/mol)	Inhibition constant (K_i_) (µM)	Final Intermolecular Energy (kcal/mol)	Final Total Internal Energy (kcal/mol)	Torsional Free Energy (kcal/mol)	Unbound System’s Energy (kcal/mol)
**2**	-8.61	0.49	-10.10	0.00	1.49	0.00
**6**	-9.41	0.13	-10.00	0.00	0.59	0.00
**10**	-9.25	0.12	-10.44	0.00	1.19	0.00
**12**	-7.96	1.47	-8.26	0.00	0.30	0.00
**14**	-10.12	0.04	-10.72	0.00	0.60	0.00
**15**	-10.59	0.02	-11.18	0.00	0.60	0.00
**16**	-10.05	0.04	-10.65	0.00	0.60	0.00
**19**	-9.79	0.07	-10.69	0.00	0.89	0.00
**20**	-8.98	0.26	-10.17	0.00	1.19	0.00
**21**	-9.51	0.11	-10.70	0.00	1.19	0.00

Significant values are in underline.

**Table 6:** Interactions of the most potent (highly active) synthesized compounds with 1OUZ.

No.	Interaction type	Amino acid residues	Distance for key Hits (Å)	No.	Interaction type	Amino acid residues	Distance for key Hits (Å)
6	H.B	HIS247	2.62	16	H.B	HIS247	1.82
	*π*-anion	ASP206	4.96		*π*-cation	HIS247	4.14
	*π*-H.B	ASN277	2.57		*π*-sulfur	MET210	4.70
	*π*-sigma	MET210	3.98		*π*-*π* stacked	TRP319	4.26
	*π*-*π* stacked	TRP319	4.67		*π*-*π* T-shaped	TYR279	5.95
14	H.B	SER171	2.52	19	H.B	HIS247	1.97
	*π*-anion	ASP168	3.88		*π*-sigma	MET210	3.49
	*π*-*π* stacked	TRP250	4.67		*π*-sulfur	MET210	4.64
	*π*-alkyl	MET210	5.29		*π*-*π* stacked	TRP250	3.92
	*π*-alkyl	ALA173	5.11		*π*-*π* stacked	TRP319	4.68
15	H.B	HIS247	2.21	21	*π*-anion	ASP206	4.71
	*π*-anion	ASP168	3.49		*π*-*π* stacked	TRP319	4.06
	*π*-sigma	MET210	3.48		*π*-*π* stacked	TRP250	4.08
	*π*-*π* stacked	TRP319	4.19		Alkyl	ALA173	4.38
	*π*-alkyl	ALA173	4.00		*π*-alkyl	TRP319	5.42

**Fig. 8:**
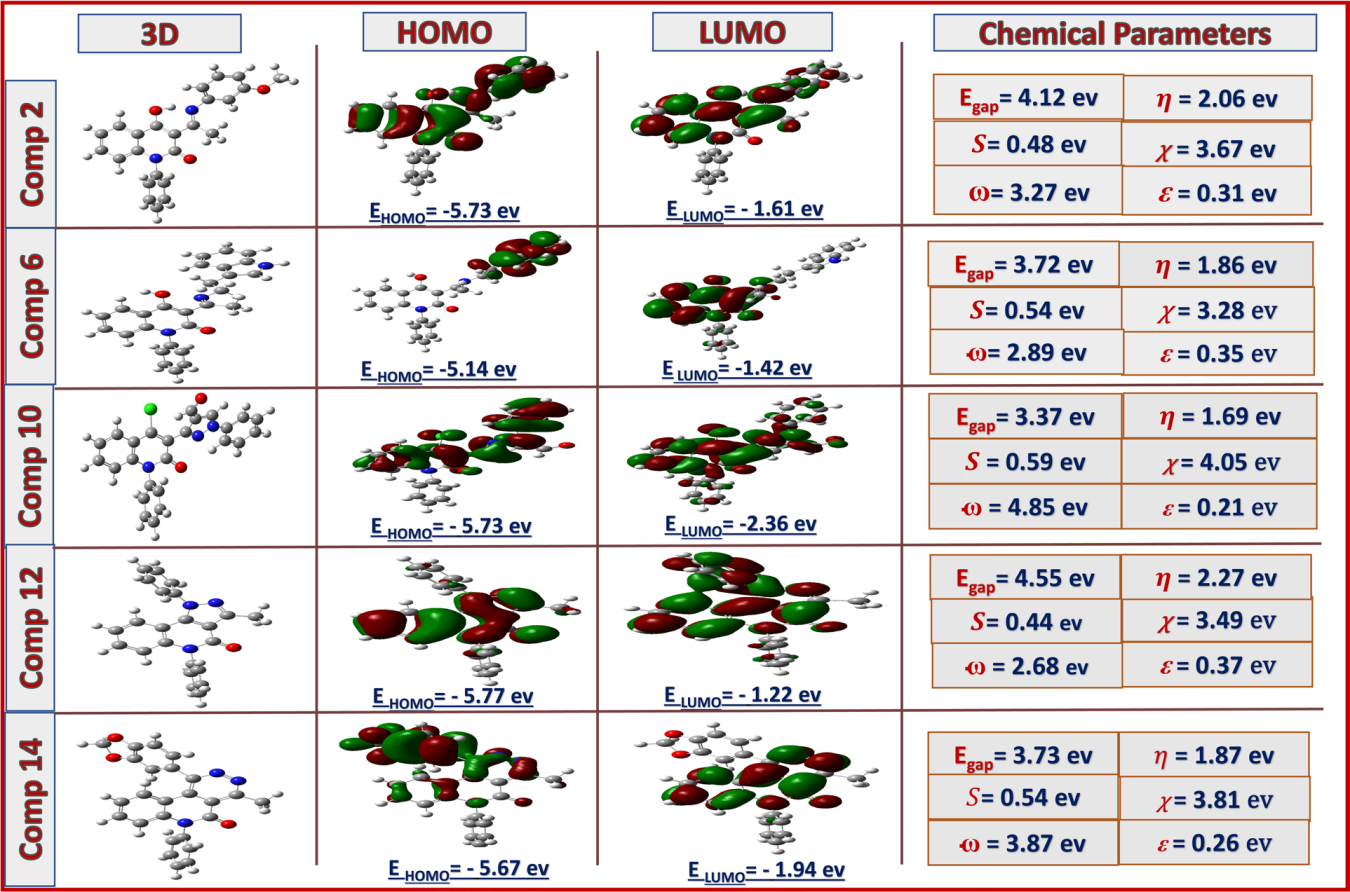
HOMO-LUMO energy level diagram of **2**, **6**, **10**, **12**, **14** and their electrochemical parameters

To correlate the structure with biological activity, studying some 3D-descriptors, such as HOMO and LUMO frontier molecular orbitals, was interesting. The chemical reactivity was reflected through the energy band gap (E_LUMO_-E_HOMO_) as an important stability index and influences the biological activities of the molecules, global hardness (*η*), global softness (*S*), electronegativity (*χ*), global electrophilicity index (*ω*), and nucleophilicity index (*ε*), that were calculated from energies of frontier molecular orbitals (E_HOMO_, E_LUMO_), by Equations (1-6) as follow Fig.8, 9:^52–54^

1 $$E_{gap} = \, E_{LUMO} - \, E_{HOMO}$$

2 $$\eta \, = \, \raise.5ex\hbox{$\scriptstyle 1$}\kern-.1em/ \kern-.15em\lower.25ex\hbox{$\scriptstyle 2$} \, \left( {E_{LUMO} {-} \, E_{HOMO} } \right)$$

3 $$S = \, 1/ \, \eta$$

4 $$\chi \, = \, - \, \raise.5ex\hbox{$\scriptstyle 1$}\kern-.1em/ \kern-.15em\lower.25ex\hbox{$\scriptstyle 2$} \, \left( {E_{HUMO} + \, E_{LOMO} } \right)$$

5 $$\omega \, = \, \mu^{2} /2\eta$$

6 $$\varepsilon = \, 1/ \, \omega$$

**Fig. 9:**
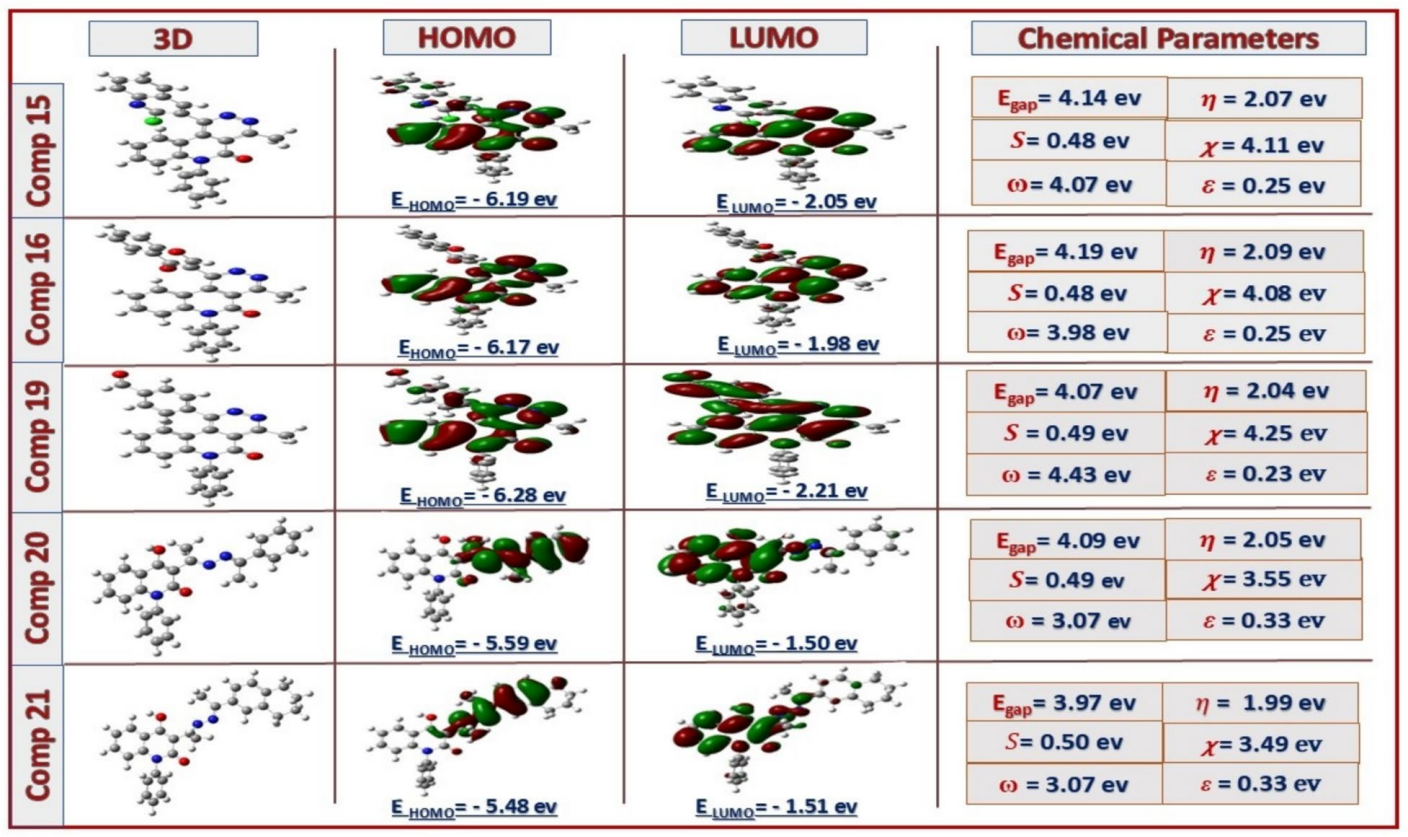
HOMO-LUMO energy level diagram of **15**, **16**, **19**- **21** and their electrochemical parameters

Compounds **14**, **15**, **16**, and **19** showed high potency in both tested biological applications with closely comparable softness values (0.48-0.54). Based on the electronic descriptors, compound **10** exhibits a lower energy gap (3.37 eV) and a high softness value. Theoretically, the presence of binary pyrazole functionality will impart a strong activity, yet it showed moderate activity compared to other tested scaffolds in the practical part. This notification may be attributed to the solubility of the compound and its lipophilicity and permeability across the lipid bilayer membrane of the cell. Whereby, the formyl, lactamic functions plus the two adjacent nitrogenous atoms in the pyrazole ring led to increasing the polar surface area (PSA); reducing the lipophilicity and thus its capability to passively diffuse over the non-polar cell membrane in contrast to the targeted drug-like profile (low water solubility and high lipophilicity)^55-57^

## Insight into structure activity relationships (SAR) against A549 and H37Rv

**Fig. 10:**
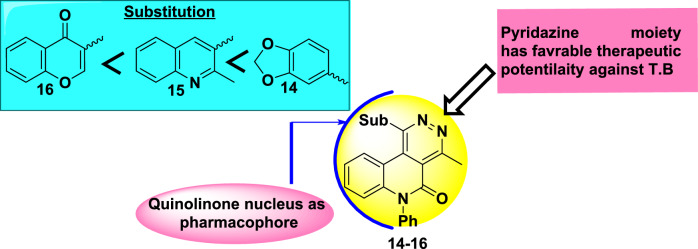
SAR estimation of fused pyridazino[4,5-*c*]quinolinones for anti-tuberculosis activity.

SAR studies show different features for the synthesized targets according to chemical modifications of active molecules that often lead to dramatically altered biological responses^58^. According to practical and molecular simulation studies, in the case of A549 cell line, hybrids **15**, **16** and **19** showed excellent potency which enhanced by the introduction of pyridazine ring fused by quinoline skeletons, resulting enhanced π-π stack interactions and established polar hydrogen interactions with the free oxygen atoms^59^ with residues of CYS773 and GLY772 for compound **16**, and ALA719, LEU 764 and THR 766 for skeleton **19**. Also, pyridazine derivatives have displayed high efficacy in patients with NSCLC as targeting the anaplastic lymphoma kinase targeting the anaplastic lymphoma kinase^60^ Whereby, benzo-1,3-dioxole, quinoline, and chromone substitutions attached to pyridazine amplified the activity due to their role in inhibition of signaling pathways involved in tumor progression and growth, including TNF-α signaling, as crucially relevant in lung cancer biology^61,62^ For Mycobacterium tuberculosis H37Rv, quinoline derivatives represent a common substructure of numerous marketed antitubercular drugs, e.g., mefloquine, bedaquiline, and fluoroquinolones. Also, quinoline hybrids are pharmacophores due to metabolic liabilities, cytotoxicity, and improving pharmacokinetic properties,^63^ where there is a certain influence of the binary benzoheterocycles substituent attached to fused pyridazino[4,5-c]quinolinone systems, the activity of these compounds increases in order benzo[*d*][1,3]dioxole> quinoline > chromone Fig. 10.

Whereas, the physicochemical properties and drug-likeness of the derivatives were calculated by the Swiss ADME program (Table 7). The derivatives presented n-octanol and water partition coefficient (MLogP) values in the range 3.37 to 4.73 (MLogP ≤ 5), the number of rotatable bonds ≤10, the number of hydrogen bond acceptors and donors in the derivatives were in the acceptable range (HBA ≤10 and HBD ≤5), and the molecular weight of all compounds was less than 500, which indicated that the derivatives met all criteria of Lipinski’s rule for five. Topological polar surface areas (TPSA) were found in the range of 39.82 to 77.99 Å^2^, and the number of rotatable bonds was less than 10, which accorded with Veber’s rule. Water solubility (cLogS) values ranged between -5.01 and -6.58, indicating soluble to moderately soluble, and the percentage oral absorption (%ABS) values of all compounds ranged from 82.09% to 95.26%, indicating that these derivatives would have good membrane permeability. As a result of this study, these compounds can be used as a new leading template in drug discovery.

**Table 7:** In-silico ADME properties of the bioactive synthesized compounds.

No.	M.wt	nRB	HBA	HBD	TPSA(Å^2^)	MLogP	cLogS	ABS%	nVs
**2**	384.43	4	4	1	63.82	3.37	-5.13	86.98	0
**6**	421.49	5	3	2	70.38	3.58	-5.56	84.71	0
**10**	425.87	4	3	0	56.89	4.16	-5.71	89.37	1
**12**	351.40	2	2	0	39.82	4.73	-5.48	95.26	1
**14**	407.42	2	5	0	66.24	3.61	-5.37	86.15	0
**15**	448.90	2	4	0	60.67	4.73	-6.58	88.07	1
**16**	431.44	2	5	0	77.99	3.38	-5.46	82.09	0
**19**	391.42	3	4	0	64.85	3.69	-5.01	86.63	0
**20**	395.45	4	4	1	66.95	3.69	-5.01	85.90	0
**21**	449.54	4	4	1	66.95	3.69	-5.01	85.90	0

M.wt molecular weight; nRB number of rotatable bonds; HBA number of hydrogen bond acceptors; HBD number of hydrogen bond donors; TPSA topological polar surface area; MLogP calculated octanol/water partition coefficient; cLogS solubility parameter; %ABS percentage of oral absorption; nVs number of violations from Lipinski’s rule of five ≤1.

**Fig. 11:**
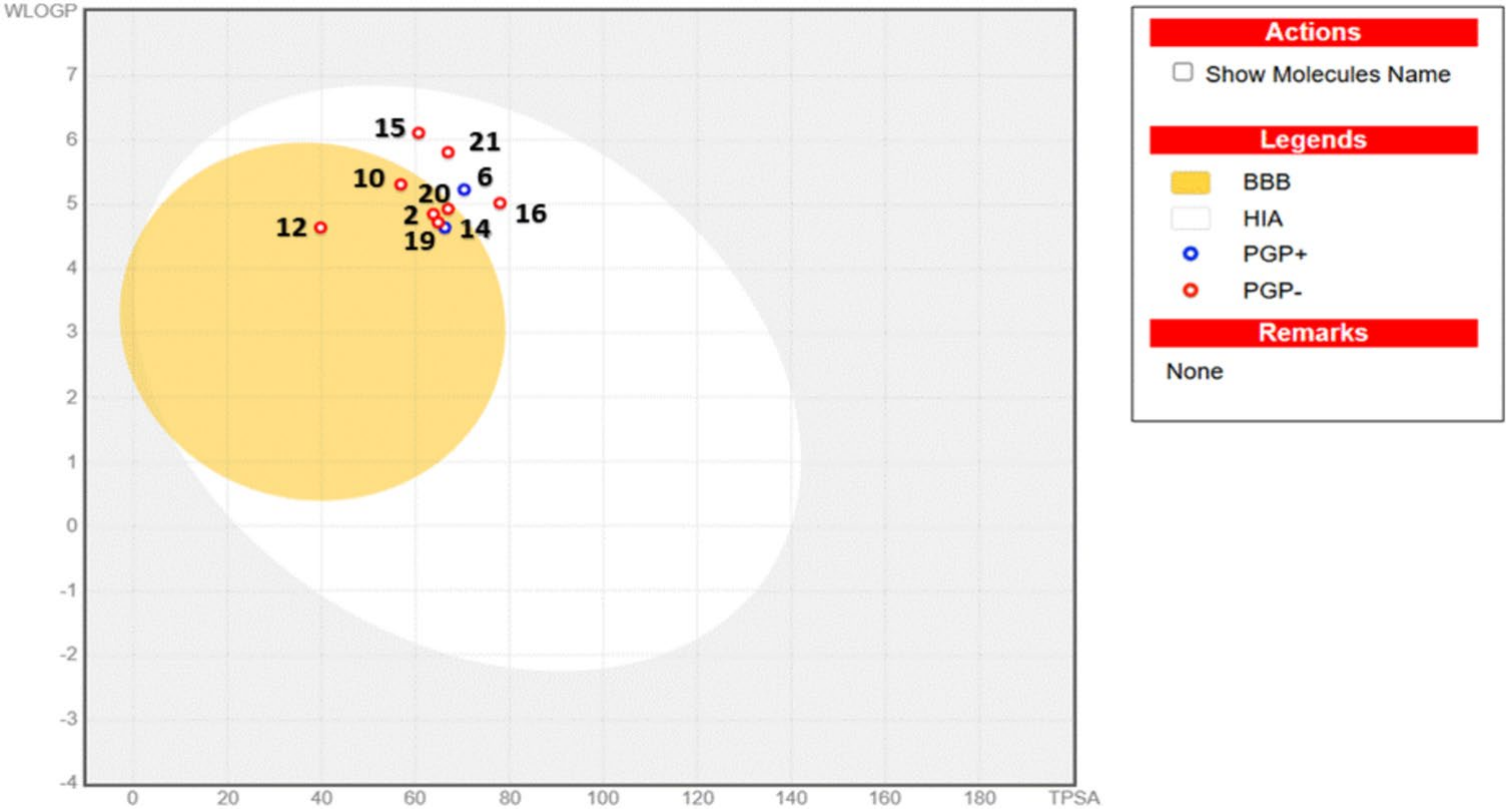
The BOILED-Egg plot of the most bioactive compounds.

ADME parameters GI (gastrointestinal absorption) and BBB (blood-brain barrier) were estimated in the BOILED-Egg graph given in Fig. 11. In this illustration, the yellow region contains areas for possible BBB permeability, and the white region contains areas for possible GI absorption. In addition, blue dots (PGP+) indicate that P-gp is an active substrate, and red dots (PGP-) indicate that P-gp is not a substrate.^64^ The presence of the investigated compounds in the outer gray region means that they have low absorption and brain permeability, and a red dot (PGP-) means that they are not predicted to be a substrate of P-gp. Compounds **2**, **10**, **12**, **15**, **16**, **19**, **20** and **21** are represented by red dots and are not PGP substrates. As a result, these derivatives met the pharmacokinetic criterion for drug-like chemical behavior, indicating that they had high oral bioavailability and could be classified as CNS medicines.

## Experimental

### General method for compounds 2, 4, and 5

Refluxing a mixture of AHQ **1** (0.279 g, 1 mmol) and the appropriate amine (1 mmol) involving *m*-anisidine or OPD, or PPD in EtOH (25 ml) for 4-6 h, the products were obtained after cooling and filtered off, then recrystallized from EtOH

#### (*E*)-4-hydroxy-3-(1-((3-methoxyphenyl)imino)ethyl)-1-phenylquinolin-2(1*H*)-one (2).

Yield, (0.299 g, 78%); off white crystals; m.p= 160-162°C; [EtOH]; R_f_: 0.64 [pet ether: ethyl acetate (4:1)]; IR (KBr) ν_max_, cm^–1^: 3420 (br, OH), 2992 (CH _Aliphatic_), 1640 (C=O, lactamic); ^1^H NMR (500 M *Hz,* DMSO-*d*_6_): *δ* (ppm) = 2.64 (s, 3H, CH_3_), 3.78 (s, 3H, OCH_3_), 6.35 (d, *J* = 8.0 *Hz*, 1H), 6.94-6.98 ( m, 3H), 7.16 (t, *J* = 7.5 *Hz*, 1H), 7.30 (d, *J* = 8.0 *Hz*, 2H), 7.39-7.43 (m, 2H), 7.51 (t, *J* = 7.5 *Hz*, 1H), 7.59 (t, *J* = 7.**7** *Hz*, 2H), 8.14 (d, *J*=8 *Hz*, 1H), 16.53 (s, 1H, OH, exchangeable D_2_O); ^13^C NMR (125 M*Hz*, DMSO-*d*_6_): *δ* (ppm) = 160.0, 137.4, 133.1, 130.3 (2C), 130.0, 129.9 (3C), 129.6 (3C), 129.3, 128.3, 126.2, 121.6, 117.6 (2C), 115.3, 113.7, 111.1 (2C), 55.4, 20.7; (EIMS) *m/z* (%): 384.61 (M^+^, 28.48), 383.46 (base peak), 338.36 (57.54), 69.64 (48.28), 67.25 (44.71), 55.20 (46.77), 45.15 (50.32), 44.07 (72.55), 40.23 (86.63); Anal. Calcd. For Chemical C_24_H_20_N_2_O_3_ (384.44): C, 74.98; H, 5.24; N, 7.29%; Found: C, 74.97; H, 5.22; N, 7.30%.

#### (*E*)-3-(1-((2-aminophenyl)imino)ethyl)-4-hydroxy-1-phenylquinolin-2(1*H*)-one (4).

Yield, (0.247 g, 67%); yellow ppt; m.p= 178-180 ^o^C; [EtOH]; R_f_ = 0.48 [pet ether: ethyl acetate (2:1)]; IR ν_max_, cm^-1^: 3421 and 3336 (NH_2_), 1634 (C=O, lactamic); ^1^H NMR (400 M *Hz,* DMSO-*d*_6_): *δ* (ppm)= 2.55 (s, 3H, CH_3_), 5.31 (s, 2H, NH_2_), 6.38 (d,* J*= 8 *Hz,* 1H), 6.65 (t,* J*= 8 *Hz,* 1H), 6.85 (d, *J* = 8 *Hz*, 1H), 7.07 (d, *J* = 8 *Hz*, 1H), 7.13 (t, *J* = 6 *Hz*, 1H), 7.19 (t, *J* = 6 *Hz*, 1H), 7.31 (d, *J* = 4 *Hz*, 2H), 7.44 (t, *J* = 8 *Hz*, 1H), 7.54 (t, *J* = 8 *Hz*, 1H), 7.63 (t, *J* = 6 *Hz*, 2H), 8.19 (d, *J*=8 *Hz*, 1H), 15.82 (s, 1H, OH);^13^C NMR (100 M *Hz,* DMSO-*d*_6_): *δ* (ppm) = 144.2 (2C), 141.9, 138.8, 133.3, 130.5, 130.4 (3C), 130.1 (3C), 129.7, 129.4, 128.7, 127.2, 126.7, 121.9, 121.5, 116.7, 116.3, 115.7, 20.6; (EIMS) *m/z* (%): 369.53 (M^+^, 17.85), 346.42 (68.89), 167.20 (56.33), 155.21 (58.13), 85.36 (base peak), 81.07 (94.74), 80.27 (99.11), 45.43 (67.46); Anal. Calcd. For Chemical C_23_H_19_N_3_O_2_ (369.42): C, 74.78; H, 5.18; N, 11.37%; Found: C, 74.77; H, 5.19; N, 11.34%.

#### (*E*)-3-(1-((4-aminophenyl)imino)ethyl)-4-hydroxy-1-phenylquinolin-2(1*H*)-one (5).

Yield, (0.269 g, 73%); mint green ppt; m.p> 300°C; [EtOH]; R_f_ = 0.47 [pet ether: ethyl acetate (2:1)]; ^1^H NMR (400 M *Hz*, DMSO-*d*_6_): *δ* (ppm)= 2.63 (s, 3H, CH_3_), 5.45 (s, 2H, NH_2_), 6.36 (d,* J*= 8.4 *Hz,* 1H), 6.65 (d, *J* = 8.4 *Hz*, 2H), 7.04 (d, *J* = 8.4 *Hz*, 2H), 7.17 (t, *J* = 7.2 *Hz*, 1H), 7.31 (d, *J* = 7.2 *Hz*, 2H), 7.40-7.44 (m, 1H), 7.53 (t, *J* = 7.4 *Hz*, 1H), 7.61 (t, *J* = 7.6 *Hz*, 2H), 8.16 (d, *J*=8 *Hz*, 1H), 15.84 (s, 1H, OH); (EIMS) *m/z* (%): 369.14 (M^+^, 24.48), 353.41 (80.90), 236.71 (83.67), 195.27 (94.34), 113.98 (90.57), 98.16 (86.64), 93.43 (93.34), 74.07 (base peak); Anal. Calcd. For Chemical C_23_H_19_N_3_O_2_ (369.42): C, 74.78; H, 5.18; N, 11.37%; Found: C, 74.75; H, 5.17; N, 11.40%.

#### (*E*)-3-(1-((2-(1*H*-indol-3-yl)ethyl)imino)ethyl)-4-hydroxy-1-phenylquinolin-2(1*H*)-one (6).

To a solution of tryptamine (1 g, 0.005 mol) in a pH 4.7 acetate buffer (50 ml) was added AHQ **1** (0.279 g, 1 mmol). The resulting solution was allowed to stand in the dark at 25 °C for 3 days. The reaction mixture was basified with 40% ammonia hydroxide, and the crystalline precipitate was filtered off and crystallized from 50% aqueous ethanol to give the final product.

Yield, (0.261 g, 62%); off white ppt; m.p= 198-200°C; [EtOH]; R_f_ = 0.44 [pet ether: ethyl acetate (2:1)]; IR ν_max_, cm^-1^: 3374 (NH), 3057 (CH_Aromatic_), 2989 (CH_Aliphatic_), 1627 (C=O, lactamic); ^1^H NMR (500 M *Hz*, CHCl_3_): *δ* (ppm)= 2.65 (s, 3H), 3.22 (br s, 2H), 3.84 (br, 2H), 6.45 (d, *J* = 8.5 *Hz*, 1H), 7.12-7.15 (m, 3H), 7.21 (t, *J* = 6.7 *Hz*, 1H), 7.28-7.31 (m, 3H), 7.37 (d, *J*=8.5 *Hz*, 1H), 7.48 (t,* J*= 7.7 *Hz,* 1H), 7.55-7.60 (m, 3H), 8.16 (s, 1H), 8.18 (d, *J* = 8 *Hz*, 1H), 15.06 (s, 1H); ^13^C NMR (125 M *Hz*, CHCl_3_): *δ* (ppm) = 136.2, 132.3, 130.0 (3C), 129.4 (3C), 128.3, 126.8, 126.5, 122.6, 122.3 (2C), 121.5, 119.6 (2C), 118.2 (2C), 115.6 (2C), 111.5 (1C), 111.4 (2C), 44.6, 25.4, 18.7; (EIMS) *m/z* (%): 421.74 (M^+^, 30.05), 369.17 (63.87), 368.18 (90.90), 355.44 (81.16), 342.36 (58.69), 211.16 (base peak), 121.06 (69.56), 84.47 (76.19); Anal. Calcd. For Chemical C_27_H_23_N_3_O_2_ (421.50): C, 76.94; H, 5.50; N, 9.97%; Found: C, 76.96; H, 5.51; N, 9.94%.

#### (*E*)-3-(1-hydrazineylideneethyl)-4-hydroxy-1-phenylquinolin-2(1*H*)-one (8).

A mixture of AHQ **1** (0.279 g, 1 mmol) and hydrazine hydrate (1 mmol) was stirred in CH_2_Cl_2_ at ambient temperature for 1h, then CH_2_Cl_2_ was evaporated, affording the product.

Yield, (0.255 g, 87%); off white sheets; m.p >300°C; [EtOH]; R_f_ = 0.53 [pet ether: ethyl acetate (2:1)]; IR ν_max_, cm^–1^: 3326 and 3238 (NH_2_), 1644 (C=O, lactamic), 1622 (C=N); ^1^H NMR (500 M*Hz*, DMSO-*d*_*6*_) *δ* 2.62 (s, 3H, CH_3_), 6.11 (s, 2H, NH_2_), 6.31 (d, *J* = 9 *Hz*, 1H), 7.11 (t, *J* = 7.7 *Hz*, 1H), 7.24 (d, *J* = 7.5 *Hz*, 2H), 7.33 (t, *J* = 7.7 *Hz*, 1H), 7.48 (t, *J*= 7.5 *Hz*, 1H), 7.56 (t, *J*=7.5 *Hz*, 2H), 8.09 (d, *J*=8.0 *Hz*, 1H), 16.25 (s, 1H, OH); ^13^C-NMR (125 M*Hz*, DMSO-*d*_*6*_): *δ* (ppm) =176.2 (1C, C-OH), 165.2 (C=O_amidic_), 163.4 (C=N), 141.3, 139.1, 132.2, 130.2 (4C), 128.6, 125.9, 121.5, 120.7, 115.4, 99.2, 16.1 (CH_3_); (EIMS) m/z (%): 293.28 (M^+^, 17.12), 246.15 (19.56), 227.67 (20.07), 193.55 (19.77), 157.22 (19.04), 117.55 (base peak), 105.29 (19.54), 65.68 (53.10); Anal. Calcd. For C_17_H_15_N_3_O_2_ (293.33): C, 69.61; H, 5.15; N, 14.33; Found: C, 69.64; H, 5.18; N, 14.32%.

### General methodology for the synthesis of 9a-9c:

After stirring mixtures of AHQ **1** (0.279 g, 1 mmol) with the appropriate hydrazine (1 mmol) involving 1-methyl-1-phenylhydrazine or 2,4,6-trichlorophenylhydrazine in EtOH (25 ml) at ambient temperature for 1 h, the obtained products were filtered off, then recrystallized from EtOH.

#### *E*)-4-hydroxy-1-phenyl-3-(1-(2-phenylhydrazineylidene)ethyl)quinolin-2(1*H*)-one (9a).

Yield, (0.277 g, 75%); yellow powder; m.p = 249-250°C; [EtOH]; lit. m.p: 249-251 °C;^41^ R_f_ = 0.53 [pet ether: ethyl acetate (2:1)]

#### (*E*)-4-Hydroxy-3-(1-(2-methyl-2-phenylhydrazineylidene)ethyl)-1-phenylquinolin-2(1*H*)-one (9b).

Yield, (0.249 g, 65%); off white crystals; m.p= 175-176°C; [EtOH]; R_f_ = 0.69 [pet ether: ethyl acetate (2:1)]; IR (reflectance), *v*_max_, cm^–1^: 1647 (C=O, lactamic); ^1^H NMR (400 M*Hz*, DMSO-*d*_6_)* δ* (ppm) = 2.82 (s, 3H, CH_3_), 3.25 (s, 3H, N-CH_3_), 6.37 (d, *J* =8.4 *Hz*,1H), 6.96-7.02 (m, 3H), 7.18 (t, *J* = 7.4 *Hz*, 1H), 7.32-3.37 (m, 4H), 7.42-7.46 (m, 1H), 7.53 (t, *J* = 7.4 *Hz*, 1H), 7.61 (t, *J* = 7.4 *Hz*, 2H), 8.14 (d, *J* = 6.4 *Hz*, 1H), 15.42 (s, 1H, OH); ^13^C NMR (125 M*Hz* , DMSO-*d*_6_): *δ* (ppm) =179.4, 177.4, 163.5, 148.6, 141.5, 138.2, 133.1, 129.9 (2C), 129.6 (2C), 129.3 (2C), 128.3, 126.3, 121.5, 121.3, 120.5, 115.3, 114.2 (2C), 99.7, 41.7, 17.0; (EIMS) *m/z* (%): 383.54 (M^+^, 20.88), 318.36 (41.97), 256.21 (42.73), 168.20 (50.42), 165.99 (base peak), 165.42 (45.95), 144.91 (41.78), 61.43 (41.52); Anal. Calcd. For Chemical C_24_H_21_N_3_O_2_ (383.45): C, 75.18; H, 5.52; N, 10.96%; Found: C, 75.21; H,5.53; N,10.92%.

#### (*E*)-4-Hydroxy-1-phenyl-3-(1-(2-(2,4,6-trichlorophenyl)hydrazineylidene)ethyl)quinolin-2(1*H*)-one (9c).

Yield, (0.359 g, 76%); off white crystals; m.p= 247-248°C; [EtOH]; R_f_ = 0.49 [pet ether: ethyl acetate (2:1)]; IR (reflectance), *v*_max_, cm^–1^: 3241 (NH), 3058 (CH_Aromatic_), 1640 (C=O, lactamic); ^1^H NMR (400 M*Hz*, DMSO-*d*_6_)* δ* (ppm)= 2.80 (s, 3H, CH_3_), 6.39 (d, *J* =8.4 *Hz*,1H), 7.19 (t, *J* = 7.4 *Hz*, 1H), 7.32 (d, *J* = 7.6 *Hz*, 2H), 7.43 (t, *J* = 7 *Hz*, 1H), 7.53 (t,* J* = 7.8 *Hz*, 1H), 7.61 (t, *J* = 7.6 *Hz*, 2H), 7.76 (s, 2H), 8.06 (d, *J* = 8 *Hz*, 1H), 8.44 (s, 1H, NH), 15.58 (s, 1H, OH); ^13^C NMR (100 M*Hz* DMSO-*d*_6_): *δ* (ppm) = 141.3, 140.6, 138.6, 138.2, 132.9, 130.6, 130.5, 130.3 (2C), 130.0 (2C), 129.9 (2C), 129.8, 129.5 (2C), 128.9 (2C), 125.9, 125.8, 122.2, 115.6, 17.8; (EIMS) *m/z* (%): 472.51 (M^+^, 24.80), 469.52 (72.96), 404.13 (72.66), 365.77 (64.90), 361.98 (59.60), 354.81 (58.99), 238.74 (68.23), 121.12 (base peak); Anal. Calcd. For Chemical C_23_H_16_Cl_3_N_3_O_2_ (472.75): C, 58.44; H, 3.41; N, 8.89 %; Found: C, 58.42; H, 3.45; N, 8.93%.

#### 3-(4-Chloro-2-oxo-1-phenyl-1,2-dihydroquinolin-3-yl)-1-phenyl-1*H*-pyrazole-4-carbaldehyde (10).

A mixture of AHQ** 1** (0.279 g, 1 mmol) and phenyl hydrazine (0.09 ml, 1 mmol) was stirred in EtOH for 3h at room temperature, affording hydrazone derivative **9a** as a yellow precipitate, after filtration and kept to dry well. Then the resulting precipitate (0.37 g, 1 mmol) was dissolved in 1.5 ml DMF and injected into a Vilsmeier reaction by adding 1 ml of POCl_3_ portion-wise. Then the mixture was stirred for 2h at room temperature, then refluxed on the steam bath for another 2 h, and poured onto crushed ice (150 g) after cooling. Then, the mixture was neutralized by Na_2_CO_3_ solution till the effervescence ceased. Eventually, the obtained precipitate was collected by filtration.

Yield, (0.229 g, 62%); buff ppt; m.p = 248-249 °C; [EtOH:DMF (1:0.5)]; R_f_ = 0.49 [ pet ether: ethyl acetate (2:1)]; IR (KBr) ν_max_, cm^–1^: 3060 (CH_Aromatic_), 1682 (C=O, formyl), 1642 (C=O, lactamic); ^1^H NMR (DMSO-*d*_6_, 500 M *Hz*): *δ* (ppm) 6.66 (d, *J* = 9 *Hz*, 1H), 7.40-7.42 (m, 4H), 7.55-7.65 (m, 6H), 7.95 (d, *J* = 7 *Hz*, 2H), 8.12 (d, *J* = 8.0 *Hz*, 1H), 9.34 (s, 1H, HC5_pyrazole_), 9.83 (s, 1H, CHO); ^13^C NMR (DMSO-*d*_6_, 125 M*Hz*): *δ* (ppm) = 184.9, 159.1, 146.9, 143.8, 140.3, 138.6, 137.1, 132.6, 132.5, 130.2, 129.8, 129.5, 129.1, 128.9, 127.9, 127.7, 126.1, 123.8, 123.6, 123.3, 119.1, 118.3, 117.9, 115.9, 110.3; (EIMS) *m/z* (%): 427.12 (M+2, 27.68), 425.14 (M^+^, 37.97), 402.42 (86.88), 378.15 (base peak), 363.23 (89.36), 362.41 (66.37), 320.46 (70.82), 318.41 (94.00); Anal. Calcd. For C_25_H_16_ClN_3_O_2_ (425.87): C, 70.51; H, 3.79; N, 9.87%; Found: C, 70.53; H, 3.78; N, 9.86 %.

#### 3-Methyl-1,9-diphenyl-1,9-dihydro-9a*H*-pyrazolo[3,4-*b*]quinoline-4,9a-diol (11).

A mixture of AHQ **1** (0.279 g, 1 mmol) and phenyl hydrazine (0.10 ml, 1 mmol) was stirred in CH_2_Cl_2_ for 2 h at ambient temperature, the formed precipitate was filtered and recrystallized. Yield, (0.277 g, 75%); pall yellow powder; m.p = 234- 236 °C; [EtOH]; R_f_ = 0.11 [pet ether: ethyl acetate (4:1)]; IR (KBr) ν_max_, cm^–1^: 3442 (OH), 3285 (OH), 3027 (CH_Aromatic_), 2949 (CH_Aliphatic_); ^1^H NMR (500 M*Hz*, DMSO-*d*_6_)* δ* (ppm) = 2.64 (s, 3H, CH_3_), 6.39 (d, *J* = 8.5 *Hz*, 1H), 6.87 (t, *J* = 7.5 *Hz*, 1H), 7.04 (d, *J* = 7.5 *Hz*, 2H), 7.19 (t, *J* = 7.5 *Hz*, 1H), 7.28-7.31 (m, 4H), 7.40 (t, *J* = 7 *Hz*, 1H), 7.51 (t, *J* = 7.5 *Hz*, 1H), 7.59 (t, *J* = 7.5 *Hz*, 2H), 8.10 (d, *J* = 8.0 *Hz*, 1H), 9.55 (s, 1H, OH), 16.23 (s, 1H, OH); ^13^C NMR (125 M*Hz*, DMSO-*d*_6_): *δ* (ppm) = 168.4, 161.9, 145.1, 140.4, 138.2, 132.1, 129.9 (2C), 129.5 (2C), 129.4 (2C), 129.3 (2C), 128.4, 124.7, 121.7, 120.3, 115.2, 112.7 (2C), 102.3, 16.8; (EIMS) *m/z* (%): 369.46 (M^+^, 24.22), 346.40 (50.34), 320.53 (76.87), 314.10 (base peak), 312.79 (55.97), 281.27 (49.58), 264.18 (57.09), 167.02 (49.71); Anal. Calcd. For Chemical C_23_H_19_N_3_O_2_ (369.42): C, 74.78; H, 5.18; N, 11.37%; Found: C, 74.76; H, 5.21; N, 11.33%.

#### 3-Methyl-1,5-diphenyl-1*H*-pyrazolo[4,3-*c*]quinolin-4(5*H*)-one (12).

A mixture of AHQ **1** (0.279 g, 1 mmol) and phenyl hydrazine hydrochloride (0.144 g, 1 mmol) was heated at 135°C for 5h in a mixed solvent system of AcOH and conc. HCl (4:1). Then the reaction mixture was left to cool at room temperature, then poured dropwise onto crushed ice (150 g). The formed precipitate was collected by filtration and recrystallized from EtOH. Yield, (0.309 g, 88%); brown powder; m.p = 260-262°C; lit. m.p: 277-278 °C;^33b^ [EtOH]; R_f_ = 0.55 [pet ether: ethyl acetate (2:1)]; IR (reflectance), cm^–1^: 1667 (C=O, lactamic), 1614 (C=N); ^1^H NMR (500 M*Hz*, DMSO-*d*_6_)* δ* (ppm) = 2.56 (s, 3H, CH_3_), 6.55 (d,* J* = 8.5 *Hz*, 1H), 7.00 (t, *J* = 7.7 *Hz*, 1H), 7.08 (d, *J* = 8 *Hz*, 1H), 7.31-7.35 (m, 3H), 7.56 (t, *J* = 7.2 *Hz*, 1H), 7.61-7.64 (m, 2H), 7.65-7.69 (m, 5H); ^13^C NMR (125 M*Hz*, DMSO-*d*_6_): *δ* (ppm)= 158.3, 147.8, 140.5, 140.2, 140.0, 137.8, 130.0 (2C), 129.9 (2C), 129.8, 129.5 (2C), 128.7, 127.3 (2C), 122.1, 121.8, 117.1 (2C), 111.1, 110.4, 12.8; (EIMS) *m/z* (%): 352.27 (M^+^+1, 26.97) 351.61 (M^+^, 35.43), 322.36 (67.95), 309.54 (64.57), 270.16 (73.77), 236.90 (89.17), 228.74 (100, base peak), 129.94 (65.69).

#### (*E*)-*N*'-(1-(4-Hydroxy-2-oxo-1-phenyl-1,2-dihydroquinolin-3yl)ethylidene)isonicotinohydra zide (13).

A mixture of AHQ **1** (0.279 g, 1 mmol) and nicotinic hydrazide (INH) (0.137 g, 1 mmol) was refluxed in ethanol (25 ml) for 3h; the formed precipitate was collected by filtration and recrystallized from ethanol.

Yield, (0.211 g, 53%); buff powder; m.p = 234- 236°C; [EtOH]; R_f_ = 0.49 [pet ether: ethyl acetate (4:1)]; IR (KBr), *v*_max_, cm^–1^: 3440 (OH), 3201 (NH), 3033 (CH_Aromatic_), 1688 (C=O, amidic), 1615 (C=O, lactamic); ^1^H NMR (500 M*Hz*, DMSO-*d*_6_)* δ* (ppm) = 2.72 (s, 3H, CH_3_), 6.38 (d, *J* =9 *Hz*,1H), 7.19 (t, *J* = 7.5 *Hz*, 1H), 7.32 (d, *J* = 7 *Hz*, 2H), 7.43 (t, *J* = 7.2 *Hz*, 1H), 7.51 (t,* J* = 7.2 *Hz*, 1H), 7.59 (t, *J* = 7.5 *Hz*, 2H), 7.86 (d, *J* = 5.5 *Hz*, 2H), 8.14 (d, *J* = 8 *Hz*, 1H), 8.80 (d, *J* = 5.5 *Hz*, 2H), 11.96 (s, 1H, NH), 16.88 (s, 1H, OH); ^13^C NMR (125 M*Hz*, DMSO-*d*_6_): *δ* (ppm) = 163.2, 150.2 (2C), 141.1, 139.5, 138.1, 132.9, 130.1, 129.9 (3C), 129.6 (3C), 129.3, 128.4, 125.6, 121.8 (3C), 121.7, 115.3, 17.6; (EIMS) *m/z* (%): 398.02 (M^+^, 44.39), 394.33 (87.66), 364.27 (57.55), 341.07 (base peak, 100), 320.08 (51.48), 217.93 (85.13), 128.31 (73.68), 119.38 (60.94); Anal. Calcd. For Chemical C_23_H_18_N_4_O_3_ (398.42): C, 69.34; H, 4.55; N, 14.06%; Found: C, 69.36; H,4.61; N,7.90%.

### General method for compounds 14-22

A mixture of hydrazone derivative **8** (0.293 g, 1 mmol) and the appropriate aldehyde or ketone (1 mmol) was refluxed in EtOH (25 ml) for 1h, the formed precipitate was filtered, then recrystallized, affording the final product.

#### 1-(Benzo[*d*][1,3]dioxol-5-yl)-4-methyl-6-phenylpyridazino[4,5-*c*]quinolin-5(6*H*)-one (14).

Yield, (0.211 g, 83%); yellow powder; m.p = 276-277 °C; [EtOH:CHCl_3_ (1:1)]; R_f_ = 0.54 [pet ether: ethyl acetate (2:1)]; IR ν_max_, cm^–1^:1643 (C=O, lactamic); ^1^H NMR (500 M*Hz*, CHCl_3_) *δ* (ppm)= 3.10 (s, 3H, CH_3_), 6.04 (s, 2H, CH_2_), 6.48 (d,* J*= 8.5 *Hz,* 1H), 6.86 (d, *J* = 7.5 *Hz*, 1H), 7.11 (d, *J* = 7.5 *Hz*, 1H), 7.16 (t, *J* = 7.2 *Hz*, 1H), 7.30 (d,* J*= 8.5 *Hz,* 2H), 7.34 (d, *J*= 7.5 *Hz,* 1H), 7.38 (s, 1H, CH), 7.49-7.52 (m, 1H), 7.59 (t, *J*= 7.5 *Hz,*2H), 8.25-8.28 (m, 1H); ^13^C NMR (125 M*Hz*, CHCl_3_): *δ* (ppm) =153.5, 150.9, 148.5, 141.6, 138.2, 132.7, 130.1 (3C), 129.4 (3C), 128.5, 127.4, 126.5, 125.5, 121.8, 115.7, 108.5 (2C), 106.0 (2C), 101.7 (2C), 17.7; (EIMS) *m/z* (%): 407.93 (M^+^, 18.39), 318.67 (72.18), 302.00 (54.27), 298.15 (80.87), 254.81 (base peak), 230.21 (47.44), 209.86 (46.35), 87.06 (61.87); Anal. Calcd. For C_25_H_17_N_3_O_3_ (407.43): C, 73.70; H, 4.21; N, 10.31, Found: C, 73.73; H, 4.19; N, 10.37%.

#### 1-(2-Chloroquinolin-3-yl)-4-methyl-6-phenylpyridazino[4,5-*c*]quinolin-5(6*H*)-one (15).

Yield, (0.323 g, 72%); brown powder; m.p >300 °C; [EtOH:CHCl_3_ (1:1)]; R_f_ = 0.63 [pet ether: ethyl acetate (2:1)]; IR ν_max_, cm^–1^: 1643 (C=O, lactamic); ^1^H NMR (400 M*Hz*, CHCl_3_) *δ* (ppm)= 3.24 (s, 3H, CH_3_), 6.52 (d,* J*= 8 *Hz,* 1H), 7.22 (t, *J* = 6.6 *Hz*, 1H), 7.34-7.41 (m, 2H), 7.56 (d, *J* = 6.4 *Hz*, 1H), 7.64-7.66 (m, 3H), 7.84 (t,* J*= 6.2 *Hz,* 1H), 7.97-8.12 (m, 2H), 8.33 (d, *J*= 6.8 *Hz,*1H), 8.79-8.92 (m, 2H); (EIMS) m/z (%): 450.13 (M^+^+2, 6.22), 448.11 (M^+^, 22.72), 371.12 (87.72), 344.09 (54.73), 338.16 (37.84), 332.10 (base peak), 139.26 (45.09), 128.07 (37.59); Anal. Calcd. For C_27_H_17_ClN_4_O (448.91):C, 72.24; H, 3.82; N, 12.48; Found: C, 72.27; H, 3.81; N, 12.46%.

#### 4-Methyl-1-(4-oxo-4*H*-chromen-3-yl)-6-phenylpyridazino[4,5-*c*]quinolin-5(6*H*)-one (16).

Yield, (0.384 g, 89%); yellow powder; m.p = 260-262 °C; [EtOH:CHCl_3_ (1:1)]; R_f_ = 0.38 [pet ether: ethyl acetate (2:1)]; IR ν_max_, cm^–1^: 1650 (*α,β*-C=O, ketonic), 1625 (C=O, lactamic); ^1^H NMR (400 M*Hz*, DMSO-*d*_*6*_) *δ* (ppm)= 3.00 (s, 3H, CH_3_), 6.36 (d,* J*= 10.5 *Hz,* 1H), 7.19 (t, *J* = 8.7 *Hz*, 1H), 7.34 (d, *J* = 9.5 *Hz*, 2H), 7.43 (t, *J* = 9 *Hz*, 1H), 7.54 (t, *J* = 9.2 *Hz*, 1H), 7.59 (t,* J*= 8 *Hz,* 1H), 7.63 (d, *J* = 9 *Hz*, 1H), 7.76 (d,* J*= 10.5 *Hz,* 1H), 7.89 (m, 1H), 8.15 (t,* J*= 7.3 *Hz,* 2H), 8.60 (s, 1H), 9.07 (s, 1H); ^13^C-NMR (100 M*Hz*, DMSO-*d*_*6*_): *δ* (ppm) =174.8, 158.0, 156.0, 138.8, 135.4, 133.7, 130.4 (3C), 130.1 (3C), 128.8 (2C), 126.9 (2C), 126.7 (2C), 125.8 (2C), 123.9, 122.1, 119.3 (2C), 117.9, 115.8, 17.9 ; (EIMS) m/z (%): 431.05 (M^+^, 13.11), 383.99 (80.43), 264.02 (87.91), 262.55 (85.94), 130.23 (68.81), 105.06 (55.98), 101.75 (base peak, 100), 76.05 (79.54); Anal. Calcd. For C_27_H_17_N_3_O_3_ (431.13): C, 75.16; H, 3.97; N, 9.74, Found: C, 75.21; H, 3.94; N, 9.70%.

#### 1-Ferroceneyl-4-methyl-6-phenylpyridazino[4,5-*c*]quinolin-5(6*H*)-one (17).

Yield, (0.297 g, 63%); burgundy powder; m.p =274-276°C; [EtOH:DMSO (1:0.5)]; R_f_ = 0.64 [pet ether: ethyl acetate (2:1)]; IR ν_max_, cm^–1^: 1638 (C=O, lactamic); ^1^H NMR (500 M*Hz*, DMSO-*d*_*6*_) *δ* (ppm)= 2.89 (s, 3H, CH_3_), 4.29 (s, 5H), 4.58 (s, 2H), 4.75 (s, 2H), 6.34 (s, 1H), 7.16-7.59 (m, 6H), 8.12 (s, 1H), 8.54 (s, 1H); (EIMS) *m/z* (%): 471.92 (M^+^, 27.34), 216.19 (base peak), 180.91 (70.05), 149.31 (61.08), 107.43 (62.51), 80.08 (58.26), 55.26 (79.91), 41.76 (57.88); Anal. Calcd. For C_28_H_21_FeN_3_O (471.34): C, 71.35; H, 4.49; N, 8.92; Found: C, 75.27; H, 4.57; N, 8.96%.

#### 4'-Dimethyl-6,6^\^-diphenyl-[1,1^\^-bipyridazino[4,5-*c*]quinoline]-5,5^\^(6*H*,6^\^*H*)-dione (18).

Yield, (0.435 g, 76%); orange powder; m.p > 300°C; [EtOH:CHCl_3_ (1:1)]; R_f_ = 0.58 [pet ether: ethyl acetate (2:1)]; IR ν_max_, cm^–1^: 1644 (C=O, lactamic); ^1^H NMR (500 M*Hz*, CHCl_3_) *δ* 3.03 (s, 6H, 2CH_3_), 6.47 (d,* J*= 8.5 *Hz,* 2H), 7.17 (t, *J* = 7.2 *Hz*, 2H), 7.29 (d, *J* = 8 *Hz*, 4H), 7.36 (t, *J* = 7.7 *Hz*, 2H), 7.53 (m, 2H), 7.60 (d,* J*= 8.5 *Hz,* 4H), 8.25 (m, 2H); ^13^C-NMR (125 M*Hz*, CHCl_3_): *δ* (ppm) = 181.4, 174.6, 167.0, 163.6, 150.2 (2C), 148.5, 142.2, 138.1, 133.5, 130.3 (3C), 130.2 (2C), 129.3 (3C), 129.1 (2C),129.0, 128.6 (2C),127.2, 126.7 (2C), 121.9 (2C), 120.0, 115.9 (2C), 115.8 (2C), 102.4, 17.6, 17.4; (EIMS) *m/z* (%): 572.00 (M^+^, 14.50), 236.72 (67.76), 208.24 (68.50), 201.04 (base peak), 171.22 (74.13), 110.63 (89.43), 104.45 (70.11), 92.50 (76.13); Anal. Calcd. For C_36_H_24_N_6_O_2_ (572.63): C, 75.51; H, 4.22; N, 14.68, Found: C, 75.54; H, 4.20; N, 14.62%.

#### 4-(4-Methyl-5-oxo-6-phenyl-5,6-dihydropyridazino[4,5-*c*]quinolin-1-yl)benzaldehyde (19).

Yield, (0.282 g, 72%); yellow powder; m.p =286-288°C; [EtOH:CHCl_3_ (1:1)]; R_f_ = 0.68 [pet ether: ethyl acetate (2:1)]; IR ν_max_, cm^–1^: 1696 (C=O, formyl), 1641 (C=O, lactamic); ^1^H NMR (500 M*Hz*, CHCl_3_) *δ* (ppm)= 3.15 (s, 3H, CH_3_), 6.49 (d,* J*= 8 *Hz,* 1H), 7.17 (t, *J* = 7.2 *Hz*, 1H), 7.31 (d, *J* = 7.5 *Hz*, 2H), 7.35 (t, *J* = 7.5 *Hz*, 1H), 7.52 (t,* J*= 7.2 *Hz,* 1H), 7.60 (t,* J*= 7.5 *Hz,* 2H), 7.93 (d, *J*= 8.5 *Hz,* 2H), 7.96 (d, *J*= 7.5 *Hz,* 2H), 8.27 (d, *J*= 7.5 *Hz,*1H), 10.07 (s*,* 1H, CH_Formyl_); ^13^C-NMR (125 M*Hz*, CHCl_3_): *δ* (ppm) = 191.4, 180.9, 174.2, 163.7, 152.3, 142.1, 138.4, 138.2, 137.9, 133.2, 132.7, 130.18, 130.12, 129.3, 129.2, 128.9, 128.6, 128.5, 128.4, 127.1, 126.5, 121.8, 115.8, 101.7, 17.6; (EIMS) *m/z* (%): 391.65 (M^+^, 27.61), 189.08 (39.97), 175.75 (39.40), 106.48 (78.26), 97.96 (63.33), 68.25 (84.78), 63.20 (base peak), 51.53 (54.34); Anal. Calcd. For C_25_H_17_N_3_O_2_ (391.43): C, 76.71; H, 4.38; N, 10.74, Found: C, 76.73; H, 4.37; N, 10.73%.

#### 4-Hydroxy-1-phenyl-3-((*E*)-1-(((*E*)-1-phenylethylidene)hydrazineylidene)ethyl)quinolin-2(1*H*)-one (20).

Yield, (0.269 g, 68%); yellow powder; m.p = 288-289 °C; [EtOH:CHCl_3_ (1:1)]; R_f_ = 0.63 [pet ether: ethyl acetate (2:1)]; IR ν_max_, cm^–1^: 2973 (CH_Aliphatic_), 1642 (C=O, lactamic); ^1^H NMR (500 M*Hz*, CHCl_3_)* δ* (ppm)= 2.63 (s, 3H, CH_3_), 3.14 (s, 3H, CH_3_), 6.50 (d,* J*= 9 *Hz,* 1H), 7.16 (t, *J* = 7.2 *Hz*, 1H), 7.30-7.34 (m, 4H), 7.44-7.48 (m, 3H), 7.50 (d,* J*= 7.5 *Hz,* 1H), 7.59 (t,* J*= 7.5 *Hz,* 2H), 7.92 (d, *J*= 8 *Hz,* 2H), 8.31 (d, *J*= 8 *Hz,* 1H), 15.72 (s, 1H, OH); ^13^C-NMR (125 M*Hz*, CHCl_3_):* δ* (ppm) = 180.8, 173.3, 164.0, 158.6, 132.8, 130.6, 130.1 (4C), 129.4 (3C), 128.6 (3C), 128.4, 127.0 (2C), 126.5, 115.7 (2C), 101.8, 17.5, 15.5; (EIMS) *m/z* (%): 395.10 (M^+^, 26.02), 303.97 (31.60), 219.15 (86.97), 213.95 (48.51), 206.23 (45.67), 176.18 (43.33), 156.43 (base peak), 155.09 (34.86); Anal. Calcd. For C_25_H_21_N_3_O_2_ (395.46): C, 75.93; H, 5.35; N, 10.63, Found: C, 75.95; H, 5.36; N, 10.57%.

#### 4-Hydroxy-1-phenyl-3-((*E*)-1-(((*E*)-1-(5,6,7,8-tetrahydronaphthalen-2-yl)ethylidene) hydrazineylidene)ethyl)quinolin-2(1*H*)-one (21).

Yield, (0.251 g, 56%); yellow powder; m.p >300 °C; [EtOH:CHCl_3_ (1:1)]; R_f_ = 0.64 [pet ether: ethyl acetate (2:1)]; IR (reflactance) ν_max_, cm^–1^: 1638 (C=O, lactamic); ^1^H NMR (500 M*Hz*, DMSO-*d*_*6*_) *δ* (ppm)= 1.75 (t, *J* = 3 *Hz*, 4H, 2CH_2_), 2.75-2.78 (m, 4H, 2CH_2_), 3.01 (s, 3H,CH_3_), 3.16 (s, 3H, CH_3_), 6.37 (d,* J*= 8 *Hz*, 1H), 7.15 (t, *J* = 8 *Hz*, 2H), 7.30 (d, *J*= 7 *Hz*, 2H), 7.38-7.42 (m, 1H), 7.51 (t,* J*= 7.7 *Hz*, 1H), 7.59 (t,* J*= 8 *Hz*, 3H), 7.65 (d, *J*= 8 *Hz*, 1H), 8.17 (d, *J*= 7 *Hz*, 1H), 17.17 (s, 1H, OH); (EIMS) *m/z* (%): 449.28 (M^+^, 16.18), 338.07 (75.36), 336.11 (base peak), 320.58 (68.72), 259.00 (70.54), 142.10 (62.46), 140.88 (79.86), 137.45 (82.18); Anal. Calcd. For C_29_H_27_N_3_O_2_ (449.55): C, 77.48; H, 6.05; N, 9.35, Found: C, 77.45; H, 6.06; N, 9.37%.

#### 4-Hydroxy-3-((*E*)-1-(((*E*)-1-(4-hydroxy-6-methyl-2-oxo-2*H*-pyran-3-yl)ethylidene) hydrazine ylidene)ethyl)-1-phenylquinolin-2(1*H*)-one (22).

Yield, (0.279 g, 63%); yellow powder; m.p =295 °C; [EtOH:CHCl_3_ (1:1)]; R_f_ = 0.64 [pet ether: ethyl acetate (2:1)]; IR ν_max_, cm^–1^: 1717 (C=O, lactonic), 1644 (C=O, lactamic); ^1^H-NMR (500 M*Hz*, CHCl_3_) *δ* (ppm)= 2.22 (s, 3H, CH_3_), 2.88 (s, 3H, CH_3_), 2.95 (s, 3H, CH_3_), 5.58 (s, 1H, CH_Pyranone_), 6.58 (d,* J*= 8 *Hz,* 1H), 7.22-7.29 (m, 3H), 7.39-7.43 (m, 1H), 7.52 (t,* J*= 7.7 *Hz,* 1H), 7.60 (t,* J*= 7.5 *Hz,* 2H), 8.23 (d, *J*= 8 *Hz,* 1H), 16.07 (s, 1H, OH), 17.30 (s, 1H, OH); ^13^C-NMR (125 M*Hz*, CHCl_3_): *δ* (ppm) = 182.3, 170.0, 169.1, 167.6, 164.4, 162.8, 141.3, 133.0, 130.2 (3C), 129.0 (2C), 128.9 (3C), 125.4, 122.3, 116.2, 115.8, 105.4, 103.4, 20.0, 18.0, 17.0; (EIMS) *m/z* (%): 443.39 (M^+^, 51.07), 393.64 (49.01), 367.05 (54.64), 341.23 (base peak), 326.18 (59.36), 228.77 (74.08), 210.61 (62.82), 189.60 (79.82); Anal. Calcd. For C_25_H_21_N_3_O_5_ (443.46): C, 67.71; H, 4.77; N, 9.48, Found: C, 67.73; H, 4.76; N, 9.47%.

## Conclusion

This approach aims to introduce novel scaffolds of quinolinone-based Schiff base, hydrazone, dihydrazone, and fused pyridazine under mild reaction conditions, high step efficiency, and substrate breadth. After that, the synthesized hybrids were evaluated as anticancer and antituberculosis candidates, showing promising anticipated results. The study’s outcomes include substantial progress in understanding the potential of synthesized quinolinone scaffolds as TB and cancer therapies by employing a multidimensional strategy that includes synthetic organic chemistry, theoretical studies, and biological evaluation. After the biological evaluations were screened, it was found that compound **15** is the most potent toward lung cancer (A549 cell line) with an IC50 value of 10.38 µM and Mtb (H37Rv) with an MIC value of 6.25 µM. Additionally, the theoretical studies, employing DFT calculations, ADME and molecular docking studies, support the biological result through offering light on the basic traits that govern their biological interactions, such as hydrogen bonding interaction with active sites of protein, and that with the help of analysis of HOMO-LUMO distributions and chemical reactivity parameters. Finally, all these descriptors present valuable guidance for drug design targeting candidates against both lung cancer and TB *via* grafting of the quinolinone scaffold with various nitrogenous moieties.

## Supplementary Information

Supplementary Information.

## Data Availability

‘Yes’ The authors declare that the data supporting the findings of this study are available within the paper and its [Supplementary Information](https:/www.nature.com/articles/s41557-022-01049-1) files. Should any raw data files be needed in another format they are available from the corresponding author upon reasonable request. [Source data](https:/www.nature.com/articles/s41557-022-01049-1) are provided with this paper.

## References

[1] Siegel, R. L., Kratzer, T. B., Giaquinto, A. N., Sung, H. & Jemal, A. Cancer statistics. *Ca.*10.3322/caac.21871 (2025). 39817679 10.3322/caac.21871 PMC11745215

[2] Lu, J. X. et al. Synthesis, anticancer activity and molecular docking of quinoline-based dihydrazone derivatives. *RSC Adv.* **15**(1), 231–243. 10.1039/d4ra06954d (2025). 39758910 10.1039/d4ra06954d PMC11694625

[3] Rogers, I. et al. The effect of comorbidities on diagnostic interval for lung cancer in England: a cohort study using electronic health record data. *Br. J. Cancer.* **131**(7), 1147–1157. 10.1038/s41416-024-02824-2 (2024). 39179794 10.1038/s41416-024-02824-2 PMC11442666

[4] Schabath, M. B. & Cote, M. L. Cancer progress and priorities: lung cancer. *Cancer Epidemiol. Biomarkers Prevention* **28**(10), 1563–1579. 10.1158/1055-9965.EPI-19-0221 (2019). 10.1158/1055-9965.EPI-19-0221 PMC6777859 31575553

[5] Nicholson, A. G. et al. The 2021 WHO classification of lung tumors: impact of advances since 2015. *J. Thorac. Oncol.* **17**(3), 362–387. 10.1016/j.jtho.2021.11.003 (2022). 34808341 10.1016/j.jtho.2021.11.003

[6] Liu, H. et al. Radiosensitization effect of quinoline-indole-Schiff base derivative 10E on non-small cell lung cancer cells *in vitro* and in tumor xenografts. *IND.* **42**(4), 405–417. 10.1007/s10637-024-01451-1 (2024). 10.1007/s10637-024-01451-1 38880855

[7] Kumbham, S. et al. A Comprehensive review of current approaches in bladder cancer treatment. *ACS Pharmacol. Transl. Sci.* **8**(2), 286–307. 10.1021/acsptsci.4c00663 (2025). 39974639 10.1021/acsptsci.4c00663 PMC11833730

[8] Cheng, J., An, Y., Wang, Q., Chen, Z. & Tong, Y. Visual detection of Mycobacterium tuberculosis in exhaled breath using N95 enrichment respirator, RPA, and lateral flow assay. *Talanta* **286**, 127490. 10.1016/j.talanta.2024.127490 (2025). 39755079 10.1016/j.talanta.2024.127490

[9] Berida, T. & Lindsley, C. W. Move over COVID, tuberculosis is once again the leading cause of death from a single infectious disease. *J. Med. Chem.* **67**(24), 21633–21640. 10.1021/acs.jmedchem.4c02876 (2024). 39652566 10.1021/acs.jmedchem.4c02876

[10] Chitale, P. et al. A comprehensive update to the M ycobacterium tuberculosis H37Rv reference genome. *Nat. Commun.* **13**(1), 7068. 10.1038/s41467-022-34853-x (2022). 36400796 10.1038/s41467-022-34853-x PMC9673877

[11] Perveen, S., Pal, S. & Sharma, R. Breaking the energy chain: importance of ATP synthase in Mycobacterium tuberculosis and its potential as a drug target. *RSC Med. Chem.* **16**, 1476–1498. 10.1039/D4MD00829D (2025). 10.1039/d4md00829d PMC11707528 39790127

[12] Verma, A., Singh, V., Chouhan, A. P. S. & Dutt, P. K. Tuberculosis: Symptoms, diagnosis, and the fight against drug resistance. *Biochem. Cellular Archives* **24**, 3337. 10.5147/bca.2024.24.1-S.3337 (2024).

[13] Qin, Y. et al. The relationship between previous pulmonary tuberculosis and risk of lung cancer in the future. *Infect. Agents Cancer.*10.1186/s13027-022-00434-2 (2022). 10.1186/s13027-022-00434-2 PMC9078090 35525982

[14] Hwang, S. Y. et al. Pulmonary tuberculosis and risk of lung cancer: a systematic review and meta-analysis. *J. Clin. Med.* **11**(3), 765. 10.3390/jcm11030765 (2022). 35160218 10.3390/jcm11030765 PMC8836400

[15] Molina-Romero, C., Arrieta, O. & Hernández-Pando, R. Tuberculosis and lung cancer. *Salud pública Méx.* **61**(3), 286–291. 10.21149/10090 (2019). 31276345 10.21149/10090

[16] Cabrera-Sanchez, J., Cuba, V., Vega, V., Van der Stuyft, P. & Otero, L. Lung cancer occurrence after an episode of tuberculosis: a systematic review and meta-analysis. *Eur. Respir. Rev.* **31**(165), 220025. 10.1183/16000617.0025-2022 (2022). 35896272 10.1183/16000617.0025-2022 PMC9724897

[17] Keikha, M. & Esfahani, B. N. The relationship between tuberculosis and lung cancer. *Adv. Biomed. Res.* **7**(1), 58. 10.4103/abr.abr_182_17 (2018). 29657943 10.4103/abr.abr_182_17 PMC5887688

[18] Odeh, D. M., Odeh, M. M., Hafez, T. S. & Hassan, A. S. Bioactive fused pyrazoles inspired by the adaptability of 5-aminopyrazole derivatives: recent review. *Molecules* **30**(2), 366. 10.3390/molecules30020366 (2025). 39860235 10.3390/molecules30020366 PMC11767260

[19] Zukić, S. et al. Data-driven modelling of substituted pyrimidine and uracil-based derivatives validated with newly synthesized and antiproliferative evaluated compounds. *Int. J. Mol. Sci.* **25**(17), 9390. 10.3390/ijms25179390 (2024). 39273338 10.3390/ijms25179390 PMC11395534

[20] Zhang, C. et al. Insights into the fluorescence and bio-activity of 2-quinolone derivatives against SHP2 from simulated and experimental aspects. *J. Mol. Struct.* **1327**, 141217. 10.1016/j.molstruc.2024.141217 (2025).

[21] Bouone, Y. O. et al. Investigation of the anticancer activity of modified 4-hydroxyquinolone analogues: *in vitro* and *in silico* studies. *RSC adv.* **15**(5), 3704–3720. 10.1039/D5RA00252D (2025). 39911545 10.1039/d5ra00252d PMC11796557

[22] Elnaggar, N. N., Hamama, W. S. & Ghaith, E. A. Vistas in the domain of 3-acetyl-4-hydroxy-2-quinolinone derivatives (AHQ) and their applications. *RSC Adv.* **15**(23), 18034–18088. 10.1039/D5RA02813B (2025). 40469234 10.1039/d5ra02813b PMC12134898

[23] Kim, M., Hong, S., Jeong, J. & Hong, S. Visible-light-active coumarin-and quinolinone-based photocatalysts and their applications in chemical transformations. *Chem. Rec.* **23**(7), e202200267. 10.1002/tcr.202200267 (2023). 36627191 10.1002/tcr.202200267

[24] Pakhariya, R. P., Bhatnagar, A. & Pemawat, G. Quinoline analogs: multifaceted heterocyclic compounds with varied synthetic strategies and potent antitubercular properties. *RSC Adv.* **15**(5), 3646–3663. 10.1039/D4RA08362H (2025). 39911544 10.1039/d4ra08362h PMC11795169

[25] Aly, A. A. et al. Catalytic syntheses of Pyrano[3,2-c]quinolone and quinoline derivatives and their potential therapeutic agents. *Current Org. Chem.* **29**(3), 181–212. 10.2174/0113852728331472240826071320 (2025).

[26] Kostopoulou, I. et al. Novel multi-target agents based on the privileged structure of 4-Hydroxy-2-quinolinone. *Molecules* **29**(1), 190. 10.3390/molecules29010190 (2024). 10.3390/molecules29010190 PMC10780633 38202773

[27] Elnaggar, N. N., Hamama, W. S., Abd El Salam, M. & Ghaith, E. A. Chemoselective synthesis of tunable poly-functionalized binary pyrazolyl and annulated pyrazolo/pyrido anchored on quinolinone: insecticidal and antioxidant studies. *RSC Adv.* **15**, 6050–6067. 10.1039/D4RA08834D (2025). 39995463 10.1039/d4ra08834d PMC11848716

[28] Dessai, P. G. et al. Design, synthesis, graph theoretical analysis and molecular modelling studies of novel substituted quinoline analogues as promising anti-breast cancer agents. *Mol. Divers.* **27**(4), 1567–1586. 10.1007/s11030-022-10512-7 (2023). 35976550 10.1007/s11030-022-10512-7

[29] He, Z. X., Gong, Y. P., Zhang, X., Ma, L. Y. & Zhao, W. Pyridazine as a privileged structure: An updated review on anticancer activity of pyridazine containing bioactive molecules. *Eur. J. Med. Chem.* **209**, 112946. 10.1016/j.ejmech.2020.112946 (2021). 33129590 10.1016/j.ejmech.2020.112946

[30] Csókás, D. et al. 2, 3-Dihydroimidazo[1,2-b]ferroceno [d] pyridazines and a 3,4-dihydro-2H-pyrimido[1,2-b] ferroceno[d]pyridazine: Synthesis, structure and *in vitro* antiproliferation activity on selected human cancer cell lines. *J. Organomet. Chem.* **750**, 41–48. 10.1016/j.jorganchem.2013.10.057 (2014).

[31] Jaballah, M. Y., Serya, R. T. & Abouzid, K. Pyridazine based scaffolds as privileged structures in anti-cancer therapy. *Drug Res.* **67**(03), 138–148. 10.1055/s-0042-119992 (2017). 10.1055/s-0042-119992 28073115

[32] Khan, F. et al. Synthesis, anticancer, α-glucosidase inhibition, molecular docking and dynamics studies of hydrazone-Schiff bases bearing polyhydroquinoline scaffold: *In vitro* and *in silico* approaches. *J. Mol. Struct.* **1321**, 139699. 10.1016/j.molstruc.2024.139699 (2025).

[33] Liu, Z. Q. et al. Recent contributions of pyridazine as a privileged scaffold of anticancer agents in medicinal chemistry: An updated review. *Bioorg. Med. Chem.* **111**, 117847. 10.1016/j.bmc.2024.117847 (2024). 39121679 10.1016/j.bmc.2024.117847

[34] Osman, E. O., Khalil, N. A., Magdy, A. & El-Dash, Y. New pyrazole–pyridazine hybrids as selective COX-2 inhibitors: design, synthesis, molecular docking, in silico studies and investigation of their anti-inflammatory potential by evaluation of TNF-α, IL-6, PGE-2 and NO in LPS-induced RAW264 7 macrophages. *RSC Med. Chem.* **15**(8), 2692–2708. 10.1039/D4MD00135D (2024). 39149111 10.1039/d4md00135d PMC11324043

[35] Stöckigt, J. et al. “The Pictet-Spengler reaction in nature and in organic chemistry. *Angew. Chem Int. Ed. Engl.* **50**, 8538–8564. 10.1002/anie.201008071 (2011). 21830283 10.1002/anie.201008071

[36] Stadlbauers, W. & Hojas, G. Ring closure reactions of 3-arylhydrazonoalkylquinolin-2-ones to 1-aryl-pyrazolo [4,3-c]quinolin-2-ones. *J. Heterocycl. Chem.* **41**, 681–690. 10.1002/jhet.5570410505 (2004).

[37] Chahal, M. et al. Unravelling the synthetic and therapeutic aspects of five, six and fused heterocycles using Vilsmeier-Haack reagent. *RSC Adv.* **13**(38), 26604–26629. 10.1039/D3RA04309F (2023). 37674485 10.1039/d3ra04309f PMC10478505

[38] Roschger, P., Fiala, W. & Stadlbauer, W. Nucleophilic substitution and ring closure reactions of 4-chloro-3-nitro-2-quinolones. *J. Heterocycl. Chem.* **29**(1), 225–231. 10.1002/jhet.5570290141 (1992).

[39] Hamama, W. S., Ghaith, E. A., Ibrahim, M., Sawamura, M. & Zoorob, H. H. Synthesis of 4-hydroxy-2-pyridinone derivatives and evaluation of their antioxidant/anticancer activities. *ChemistrySelect* **6**, 1430–1439. 10.1002/slct.202004682 (2021).

[40] Barathan, M., Shivashekaregowda, N. K. H., Hoong, S. M., Vellasamy, K. M. & Vadivelu, J. Anticancer effect of aromatic isoniazid derivatives in human gastric adenocarcinoma cells. *Toxicol. Appl. Pharmacol.* **481**, 116767. 10.1016/j.taap.2023.116767 (2023). 38007073 10.1016/j.taap.2023.116767

[41] Lone, M. S. et al. Isonicotinoyl-butanoic acid hydrazone derivatives as anti-tubercular agents: *In-silico* studies, synthesis, spectral characterization and biological evaluation. *Med. Chem. Res.* **32**(5), 808–826. 10.1007/s00044-023-03039-5 (2023).

[42] Rouzi, K. et al. Novel isoniazid-hydrazone derivatives induce cell growth inhibition, cell cycle arrest and apoptosis *via* mitochondria-dependent caspase activation and PI3K/AKT inhibition. *Bioorg. Chem.* **150**, 107563. 10.1016/j.bioorg.2024.107563 (2024). 38885547 10.1016/j.bioorg.2024.107563

[43] Jung, C. et al. Formal [4+2] combined ionic and radical approach of vinylogous enaminonitriles to access highly substituted sulfonyl pyridazines. *Commun. Chem.* **7**(1), 281. 10.1038/s42004-024-01368-z (2024). 39616260 10.1038/s42004-024-01368-z PMC11608332

[44] Denizot, F. & Lang, R. Rapid colorimetric assay for cell growth and survival: Modifcations to the tetrazolium dye procedure giving improved sensitivity and reliability. *J. Immunol. Methods.* **89**(2), 271–277. 10.1016/0022-1759(86)90368-6 (1986). 3486233 10.1016/0022-1759(86)90368-6

[45] Van de Loosdrecht, A. A., Beelen, R. H. J., Ossenkoppele, G., Broekhoven, M. G. & Langenhuijsen, M. M. A. C. A tetrazolium-based colorimetric MTT assay to quantitate human monocyte mediated cytotoxicity against leukemic cells from cell lines and patients with acute myeloid leukemia. *J. Immunol. Methods.* **174**(1–2), 311–320. 10.1016/0022-1759(94)90034-5 (1994). 8083535 10.1016/0022-1759(94)90034-5

[46] Abad, N. et al. Molecular drug design, theoretical, experimental approaches and new framework of novel oxazol dihydroquinoxaline (ODQ): Efficient synthesis, crystallographic, computational investigation, DFT calculation, ADME analysis and antiangiogenic molecular docking. *J. Mol. Struct.* **1321**, 139762. 10.1016/j.molstruc.2024.139762 (2025).

[47] Ahmed, N. A., Abdelazem, N. S., Mohamed, A. Z., El-Sayed, N. A. & Ahmed, S. A. Design, synthesis, biological evaluation, *in silico* ADME prediction and molecular docking of pyrazole-benzamides as multitargeting protein kinase inhibitors. *J. Mol. Struct.* **1288**, 135753. 10.1016/j.molstruc.2023.135753 (2023).

[48] Patel, C. N. & Narechania, M. B. Targeting epidermal growth factor receptors inhibition in non-small-cell lung cancer: a computational approach. *Mol. Simul.* **44**(17), 1478–1488. 10.1080/08927022.2018.1515484 (2018).

[49] Zhang, N. & Li, Y. Receptor tyrosine kinases: Biological functions and anticancer targeted therapy. *Med. Comm* **4**(6), e446. 10.1002/mco2.446 (2023). 10.1002/mco2.446 PMC10701465 38077251

[50] De Ávila, M. B., Bitencourt-Ferreira, G. & de Azevedo, W. F. Structural basis for inhibition of enoyl-[acyl carrier protein] reductase (InhA) from Mycobacterium tuberculosis. *Curr. Med. Chem.* **27**(5), 745–759. 10.2174/0929867326666181203125229 (2020). 30501592 10.2174/0929867326666181203125229

[51] Chingizova, E. A., Novikova, O. D., Portnyagina, O. Y. & Aminin, D. L. Components of bacterial cell walls as targets for searching for new antibacterial compounds: methods of study. *Mol. Biol.* **59**(3), 293–319. 10.1134/S0026893325700013 (2025). 10.31857/S0026898425030019 40864854

[52] Ghaith, E. A., Zoorob, H. H. & Hamama, W. S. Synthesis, antimicrobial evaluation, DFT, and molecular docking studies of pyrano[4,3-*b*]pyranone and pyrano[2,3-*b*]pyridinone systems. *Chem. Biodivers.* **21**(5), e202400243. 10.1002/cbdv.202400243 (2024). 38462494 10.1002/cbdv.202400243

[53] Ghaith, E. A., Abd El Salam, M. & Said, G. E. Barbiturate and uracil scaffolds as potential insecticidal agents: Synthesis, DFT studies and in vivo Biochemical Susceptibility of the polyphagous pest, cotton leafworm, *Spodoptera littoralis* (*Lepidoptera: Noctuidae*). *J. Mol. Struct.* **1338**, 142302 (2025).

[54] Ghaith, E. A., Zoorob, H. H., Ibrahim, M. E., Sawamura, M. & Hamama, W. S. Convenient synthesis of binary and fused pyrazole ring systems: accredited by molecular modeling and biological evaluation. *ChemistrySelect* **5**, 14917–14923. 10.1002/slct.202004014 (2020).

[55] Ecker, A. K., Levorse, D. A., Victor, D. A. & Mitcheltree, M. J. Bioisostere effects on the EPSA of common permeability-limiting groups. *ACS Med. Chem. Lett.* **13**(6), 964–971. 10.1021/acsmedchemlett.2c00114 (2022). 35707148 10.1021/acsmedchemlett.2c00114 PMC9190035

[56] Li, G. et al. Pyrazole-containing pharmaceuticals: target, pharmacological activity, and their SAR studies. *RSC med. chem.* **13**(11), 1300–1321. 10.1039/D2MD00206J (2022). 36439976 10.1039/d2md00206j PMC9667768

[57] Antiqueira-Santos, P. et al. Synthesis of pyrazoline fatty chain derivatives and its effects on melanoma cells. *Bioorg. Med. Chem. Lett.* **41**, 127988. 10.1016/j.bmcl.2021.127988 (2021). 33775838 10.1016/j.bmcl.2021.127988

[58] Ancajas, C. M. F., Oyedele, A. S., Butt, C. M. & Walker, A. S. Advances, opportunities, and challenges in methods for interrogating the structure-activity relationships of natural products. *Nat. Prod. Rep.* **41**, 1543–1578. 10.1039/D4NP00009A (2024). 38912779 10.1039/d4np00009a PMC11484176

[59] Verma, S., Lal, S. & Narang, R. Expanding potential of quinoline hydrazide/hydrazone derivatives as anticancer agents. *Future Med. Chem.* **16**(13), 1283–1286. 10.1080/17568919.2024.2366150 (2024). 38934366 10.1080/17568919.2024.2366150 PMC11318745

[60] Shaalan, M. M. et al. Novel 3, 6-disubstituted pyridazine derivatives targeting jnk1 pathway: scaffold hopping and hybridization-based design, synthesis, molecular modeling, and *in vitro* and *in vivo* anticancer evaluation. *ACS Omega* **9**(35), 37310–37329. 10.1021/acsomega.4c05250 (2024). 39246493 10.1021/acsomega.4c05250 PMC11375727

[61] Gaspar, A., Matos, M. J., Garrido, J., Uriarte, E. & Borges, F. Chromone: a valid scaffold in medicinal chemistry. *Chem. Rev.* **114**(9), 4960–4992. 10.1021/cr400265z (2014). 24555663 10.1021/cr400265z

[62] Al-Wahaibi, L. H. et al. Design, synthesis, and biological evaluation of novel quinoline-based EGFR/HER-2 dual-target inhibitors as potential anti-tumor agents. *RSC adv.* **14**(45), 32978–32991. 10.1039/D4RA06394E (2024). 39434991 10.1039/d4ra06394e PMC11492426

[63] Pitta, E. et al. Searching for new leads for tuberculosis: design, synthesis, and biological evaluation of novel 2-quinolin-4-yloxyacetamides. *J. Med. Chem.* **59**(14), 6709–6728. 10.1021/acs.jmedchem.6b00245 (2016). 27348630 10.1021/acs.jmedchem.6b00245

[64] Panyatip, P., Nunthaboot, N. & Puthongking, P. *In silico* ADME, metabolism prediction and hydrolysis study of melatonin derivatives. *Int. J. Tryptophan Res.* **13**, 1–7. 10.1177/1178646920978245 (2020). 10.1177/1178646920978245 PMC7745548 33402831

