# Supplementary material for: Rational design, synthesis of some quinolinone-Schiff bases/ pyridazino[4,5-c]quinolinones with potent anti-lung cancer and antituberculosis performance
Source: Sci Rep. 2025 Nov 4;15:38497. doi: 10.1038/s41598-025-22255-0 (PMC12586691; doi:10.1038/s41598-025-22255-0)
Supplement: Supplementary file 1 — Supplementary Information. [file 41598_2025_22255_MOESM1_ESM.docx]

**Rational Design, synthesis of some quinolinone-Schiff bases/ pyridazino[4,5-c]quinolinones with potent anti-lung cancer and antituberculosis performance**

**Eslam A. Ghaith, Nedaa N. Elnaggar, Wafaa S. Hamama,***

*Chemistry Department, Faculty of Science, Mansoura University, 35516, Mansoura, Egypt*

*^*^Corresponding author:* wshamama@mans.edu.eg; [wshamama53@gmail.com](mailto:wshamama53@gmail.com)

**3. Experimental**

**3.1. Chemistry**

**General information**

Melting points (uncorrected) were determined on a Gallenkamp electric melting point apparatus. The IR spectra were recorded (KBr) on a Mattson 5000 FTIR spectrophotometer (ν, cm^-1^) or reflectance Bruker. The ^1^H and ^13^C NMR spectra were recorded on JEOL ECA II (^1^H at 500 M*Hz*, ^13^C at 125 M*Hz*) or Bruker Advance spectrometers (^1^H at 400 M*Hz*, ^13^C at 100 M*Hz*) in CDCl_3_ and DMSO-d_6_. Mass spectra were determined at 70 eV on Varian MAT 311Kratos instrument. Elemental analyses (C, H and N) were carried out, (the experimental results are coincided with the calculated values of the proposed molecular structural formulas). All reactions were monitored using thin layer chromatography (TLC) on aluminum sheets pre-coated with Al_2_O_3_ with fluorescent indicator F254, Merck (Darmstadt, Germany). Detection of reactions were carried out through TLC silica gel GF254 under UV light at 254/365 nm.

**3-acetyl-4-hydroxy-1-phenylquinolin-2(1*H*)-one (1).**

A mixture of equal molar ratio of diphenylamine and diethyl malonate was refluxed in heating mantle for 3h till the calculated amount of EtOH was collected *via* Dean Stark by a short Vigreux column as distillation system afforded the lactone product followed by alkaline hydrolysis [NaOH (2N)] then acidified with HCl (2N) to yield the product **1**, the crude product was obtained after recrystallized with MeOH.

Yield, 86%; beige powder; m.p = 234-236 ^o^C; [EtOH]; R_f_ = 0.53 [pet ether: ethyl acetate (4:1)]; IR (KBr) ν_max_, cm^–1^: 3073 (CH Aromatic), 1887 (C=O, ketonic), 1655 (C=O, lactamic); ^1^H NMR (500 M*Hz*, DMSO-*d_6_* ) *δ* (ppm) = 2.79 (s, 3H), 6.58 (d, 1H, J=9 *Hz*), 7.21-7.26 (m, 1H), 7.27 (d, 2H, *J*=8 Hz), 7.45 (t, 1H, *J*=8 *Hz*), 7.53 (t, 1H, *J*=7 *Hz*), 7.61 (t, 2H, *J*=7.7 *Hz*), 8.24 (d, 1H, *J*=8 *Hz*), 17.16 (s, 1H, OH); ^13^C-NMR (125 M*Hz*, DMSO-*d_6_* ): *δ* (ppm) = 206.9 (1C), 175.0 (1C), 161.9 (1C), 142.5 (1C), 137.4 (1C), 134.4 (1C), 130.3 (2C), 129.0 (2C), 125.8 (1C), 122.3 (1C), 117.7 (1C), 116.0 (1C), 115.2 (1C), 106.0 (1C), 31.4 (1C).


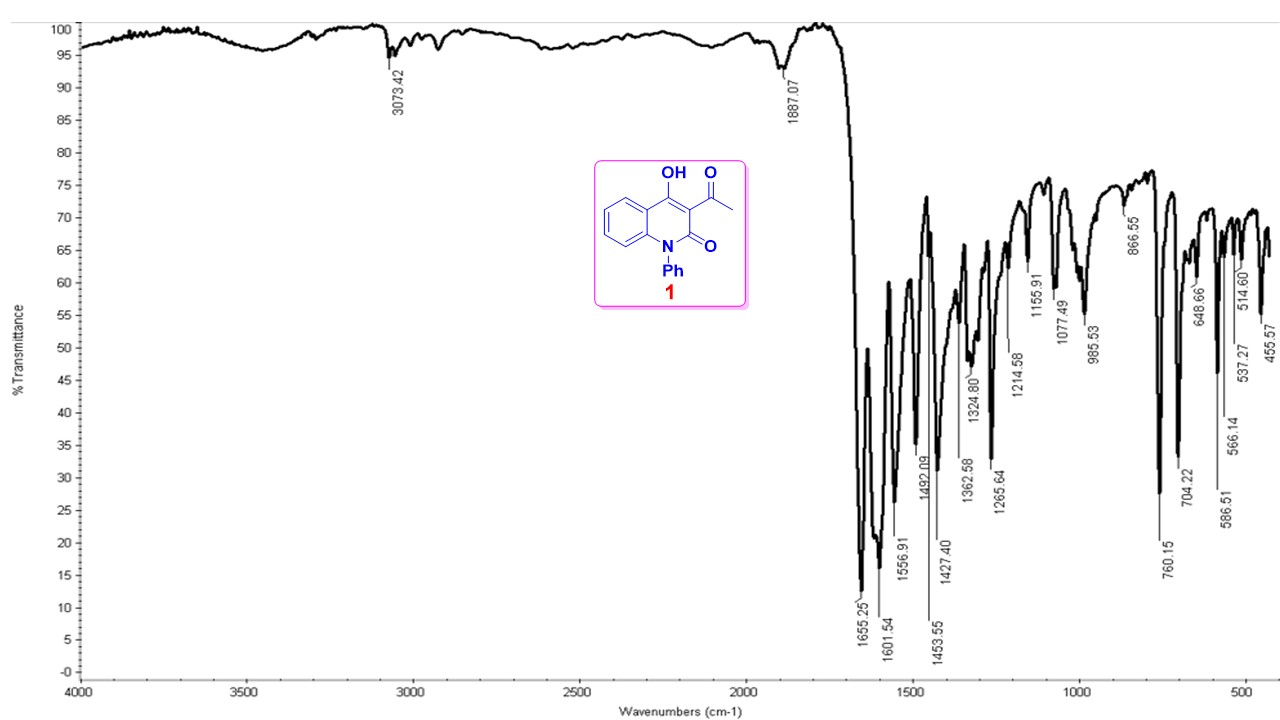


**Figure 1: IR Spectrum of compound 1**

**
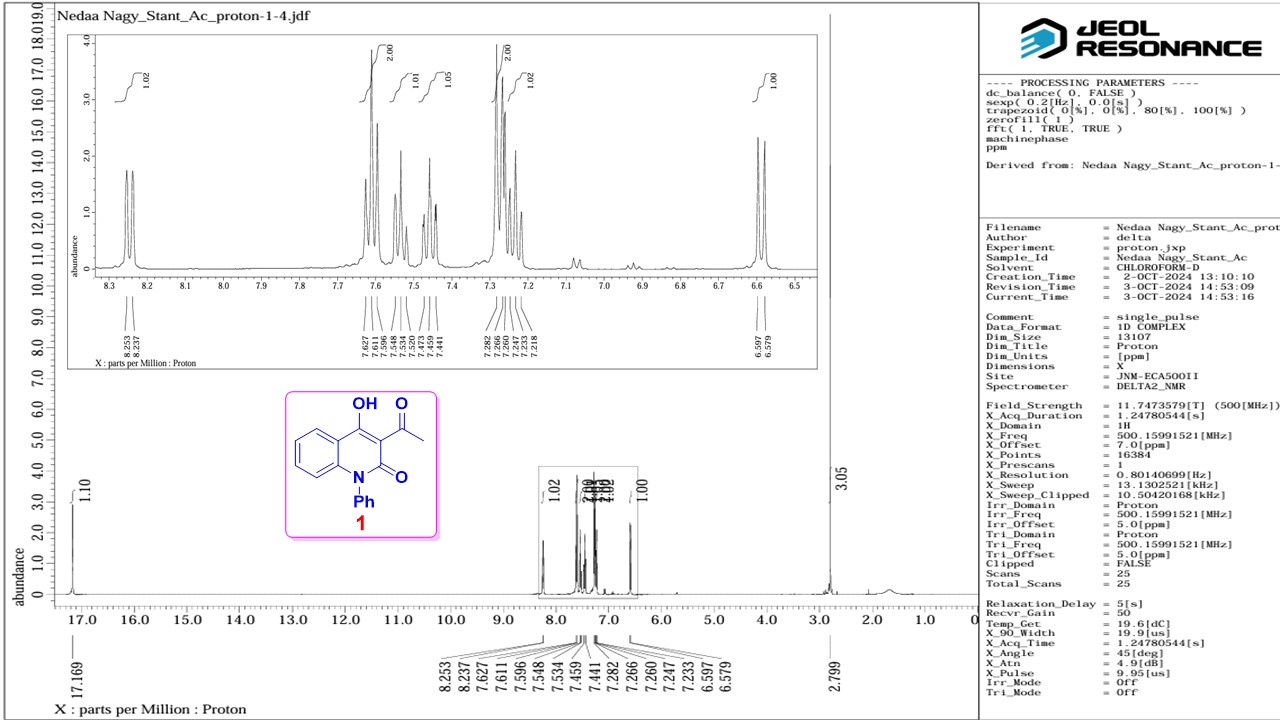
Figure 2: ^1^H NMR Spectrum of compound 1**

**
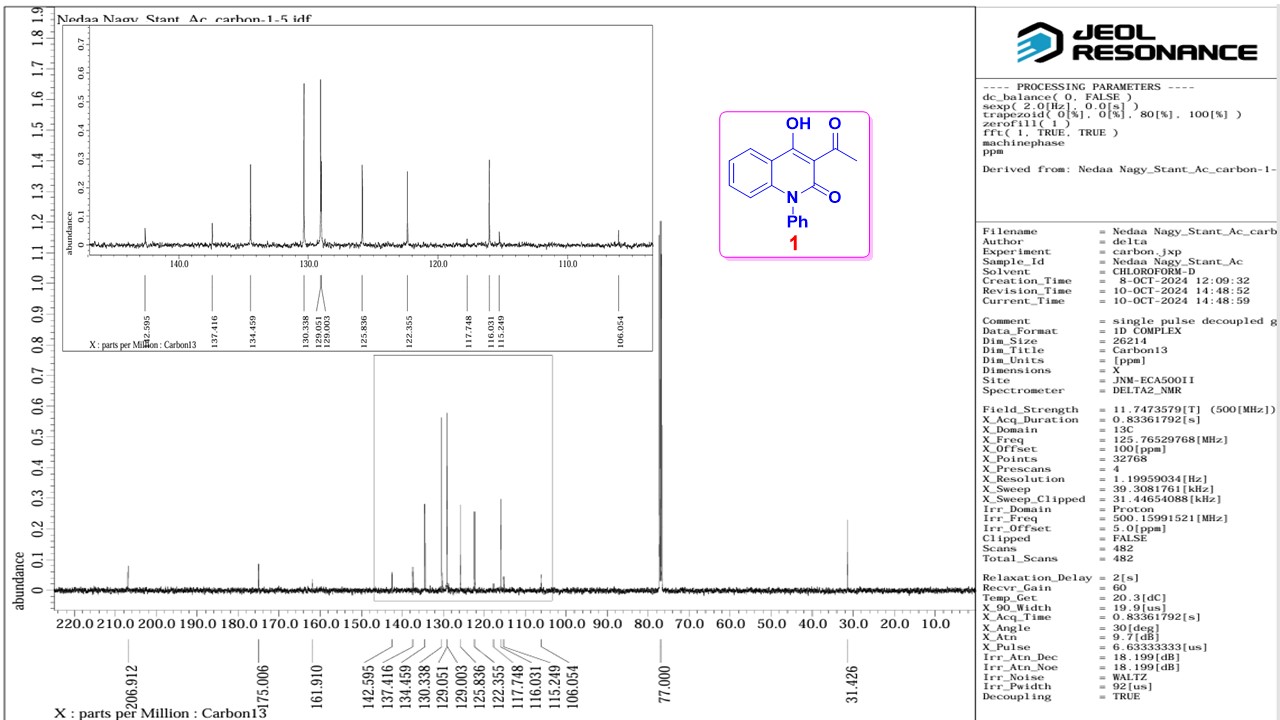
Figure 3: ^13^C NMR Spectrum of compound 1**

**
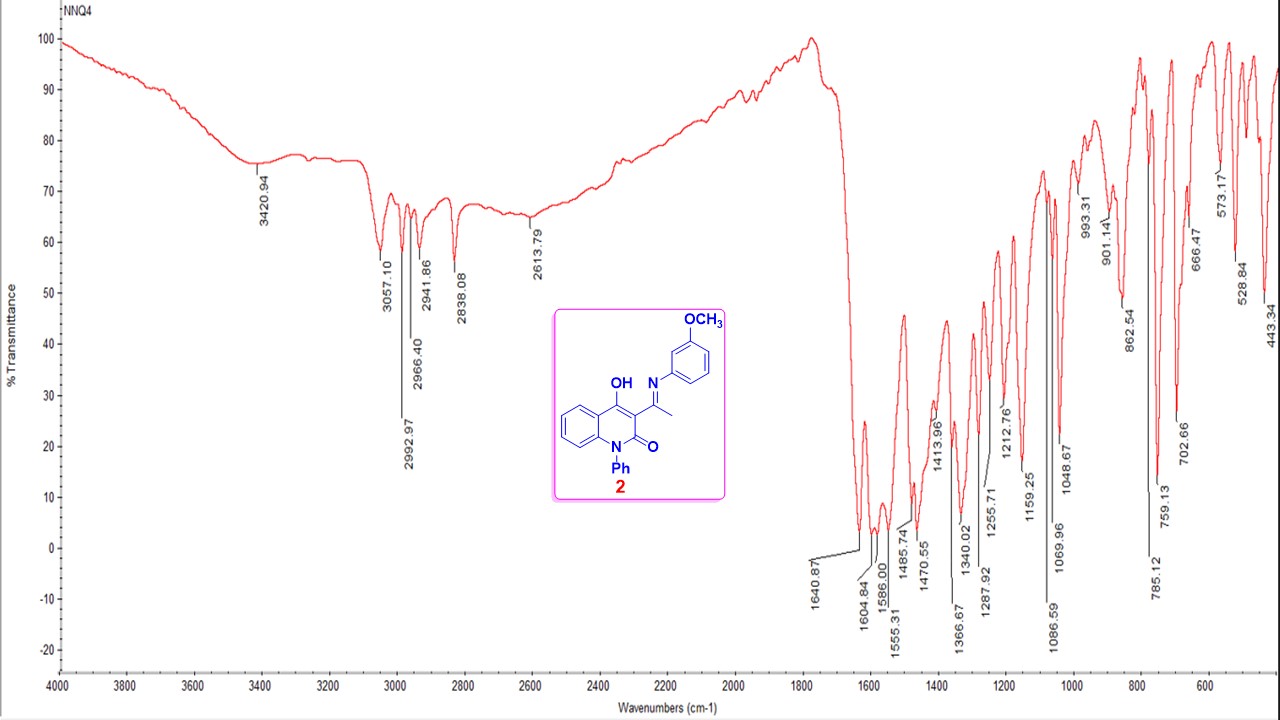
**

**Figure 4: IR Spectrum of compound 2**


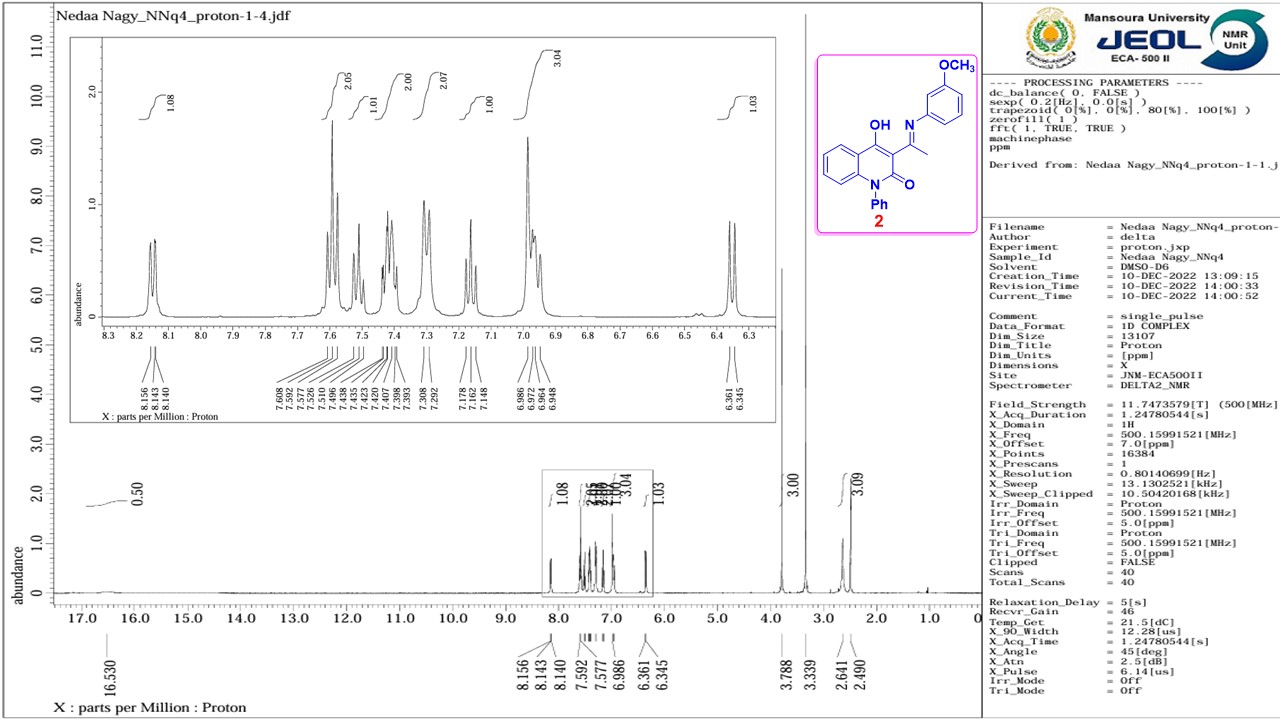
**Figure 5: ^1^H NMR Spectrum of compound 2**

**
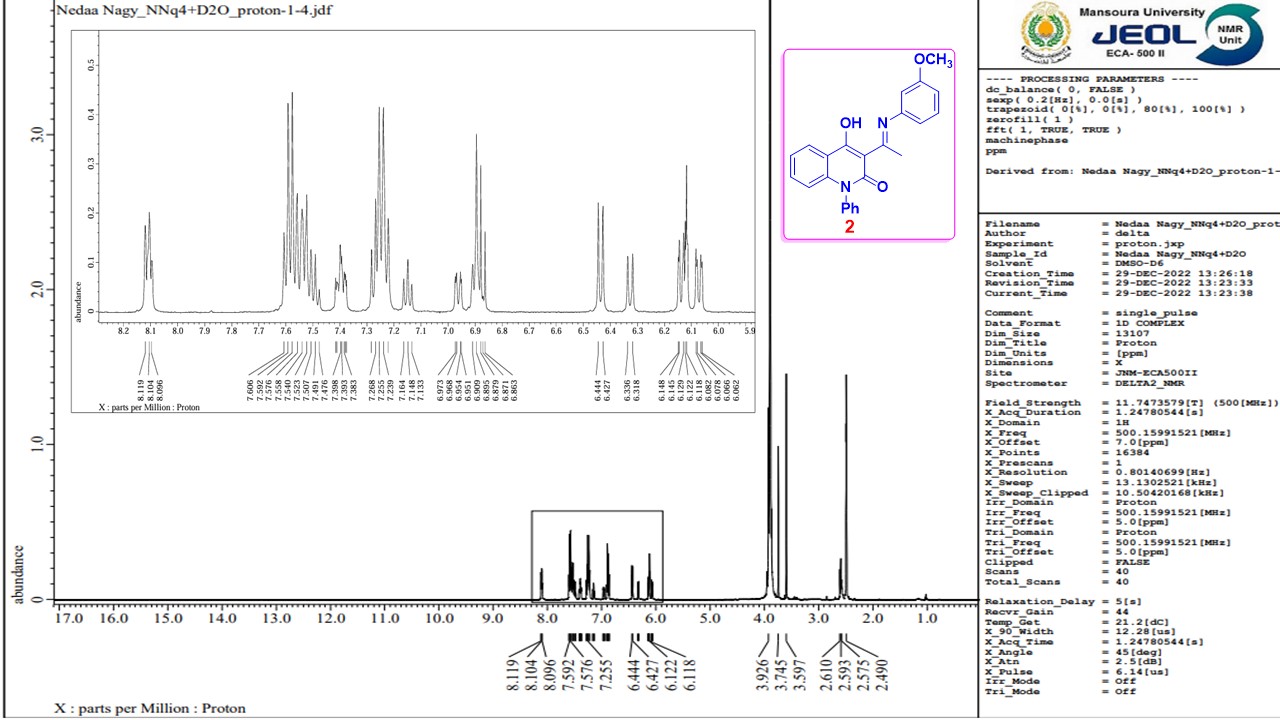
**

**Figure 6: D_2_O NMR Spectrum of compound 2**

**
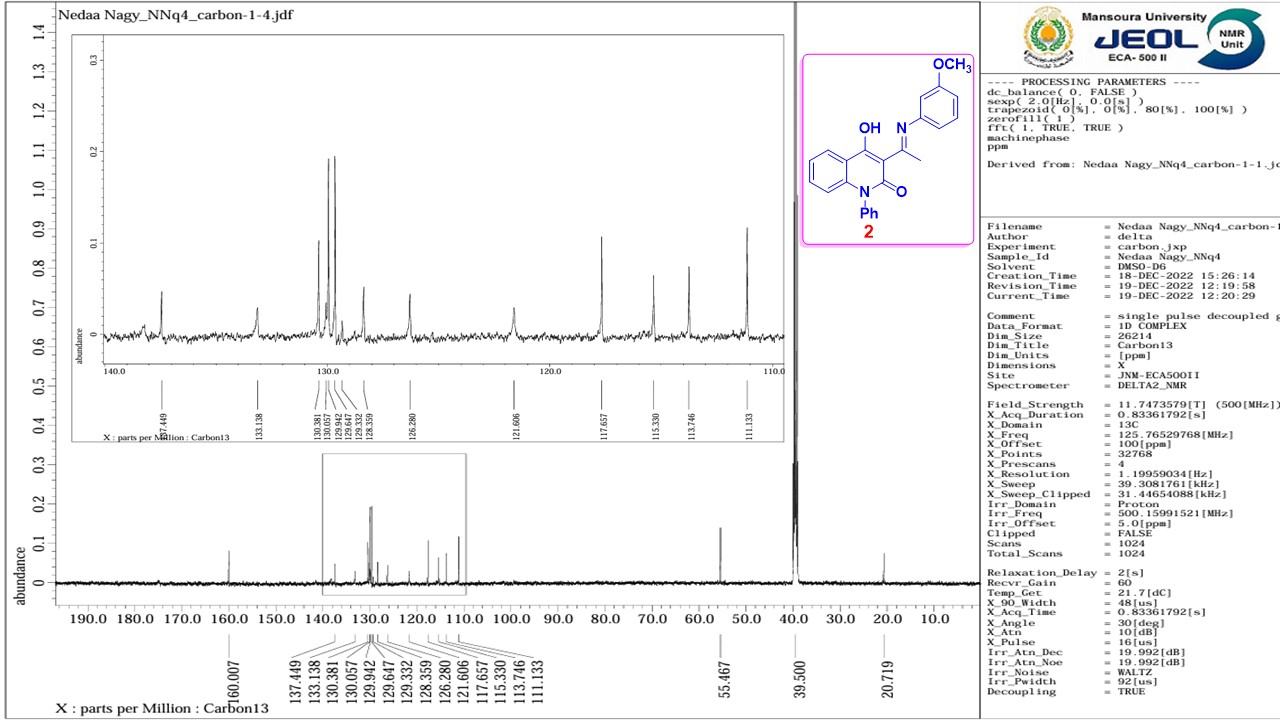
**

**Figure 7: ^13^C NMR Spectrum of compound 2**

**
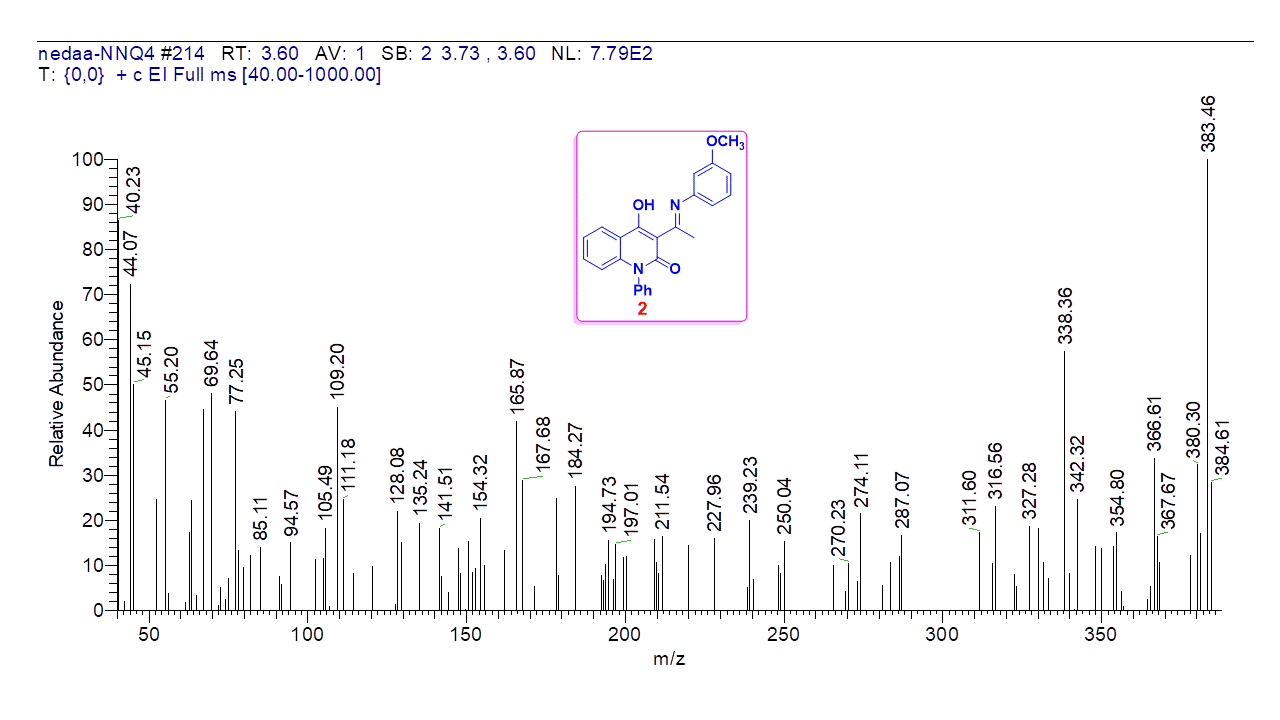
**

**Figure 8: Mass spectrometry of compound 2**

**
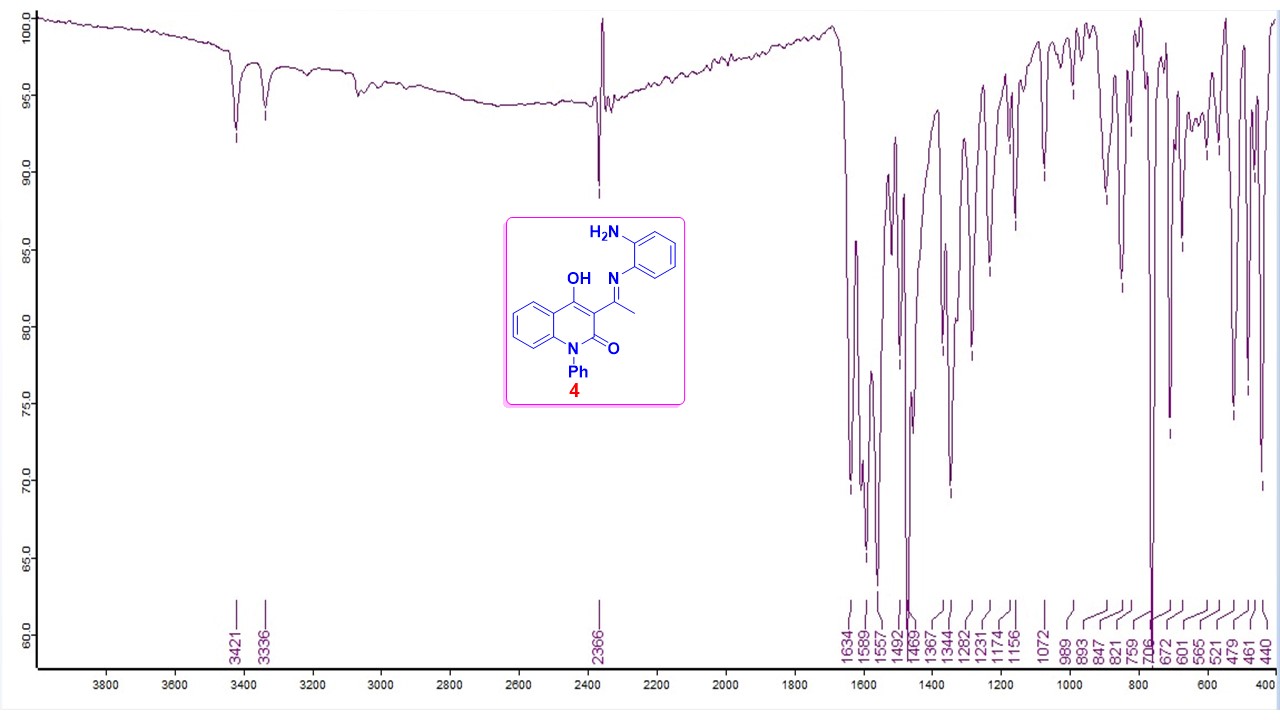
Figure 9: IR Spectrum of compound 4**

**
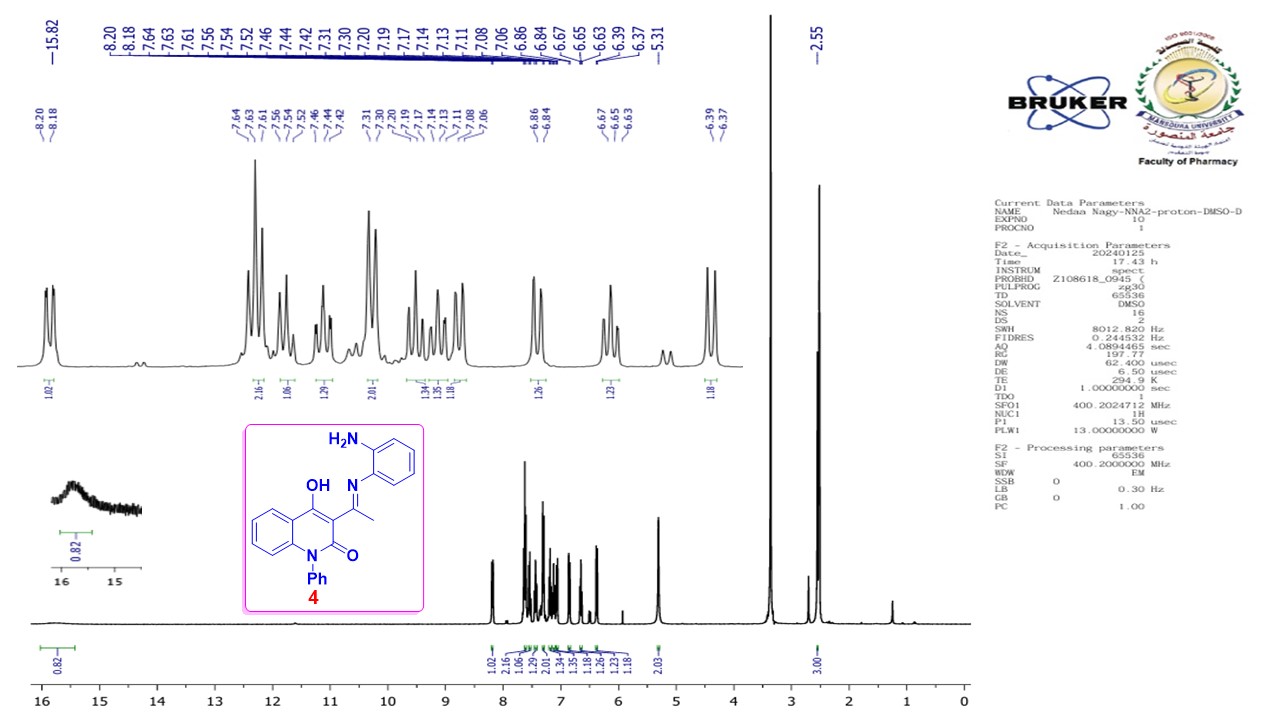
**

**Figure 10: ^1^H NMR Spectrum of compound 4**

**
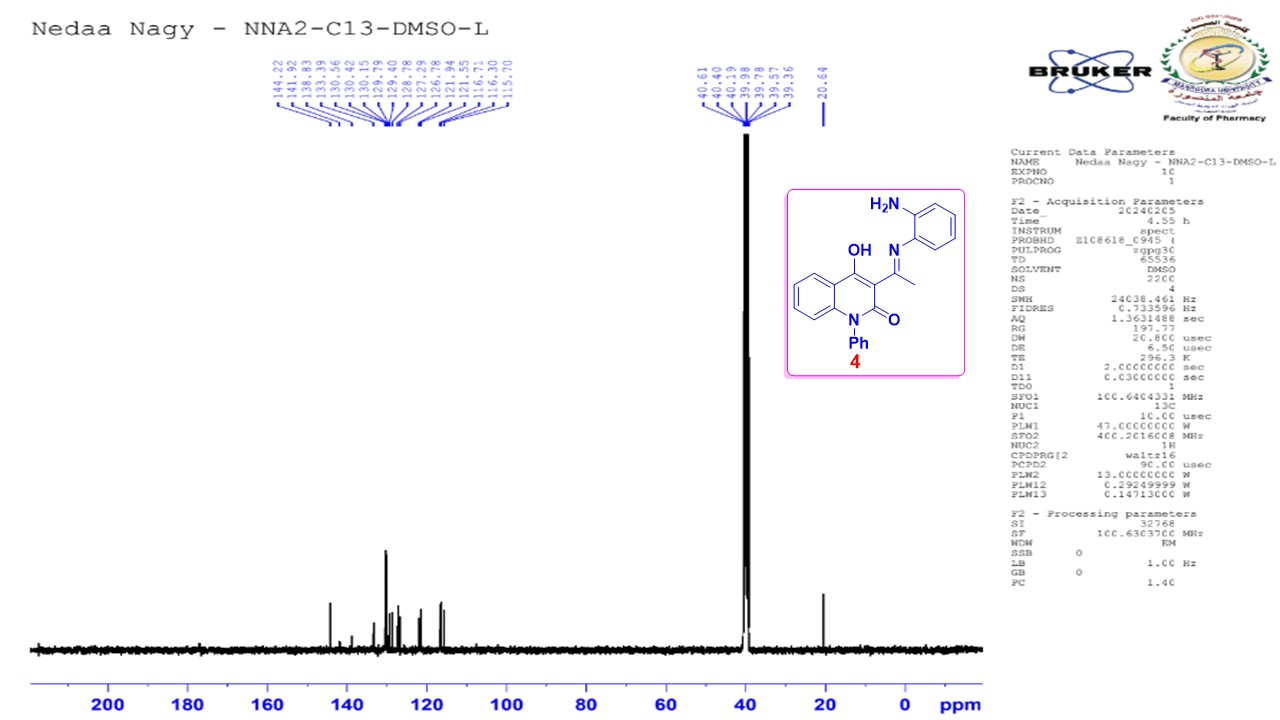
Figure 11: ^13^C NMR Spectrum of compound 4**

**
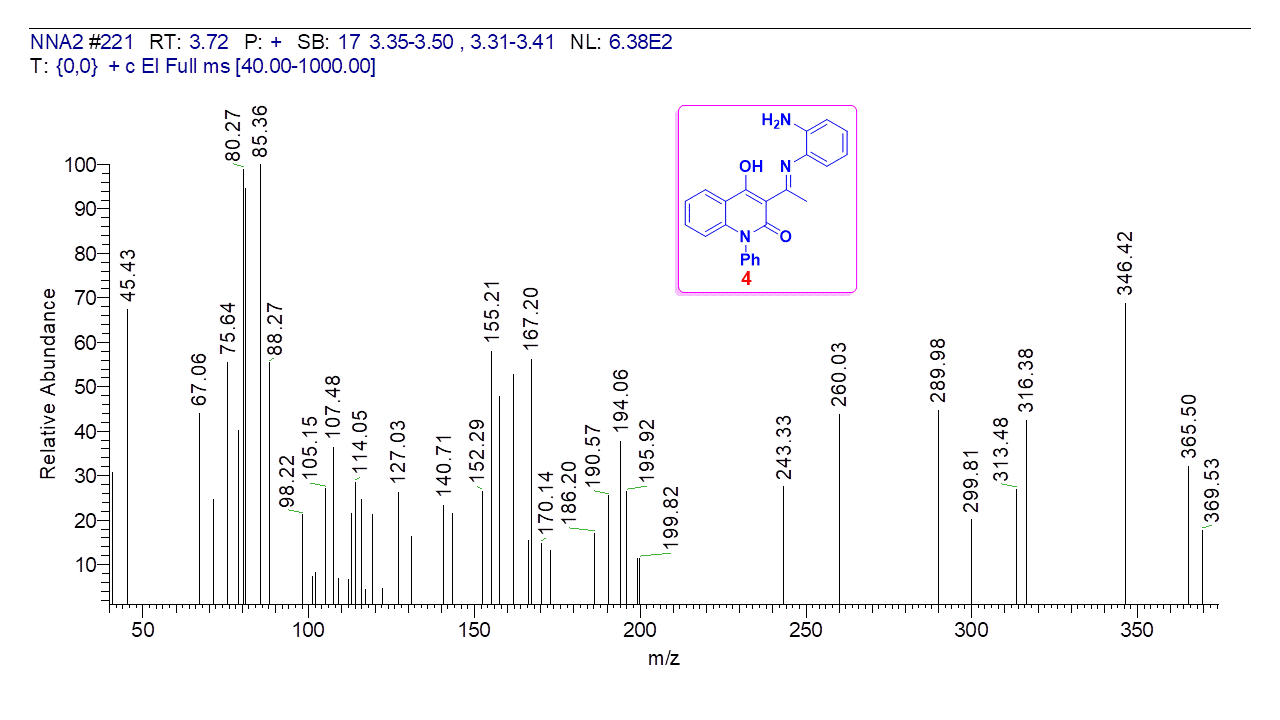
**

**Figure 12: Mass spectrometry of compound 4**

**
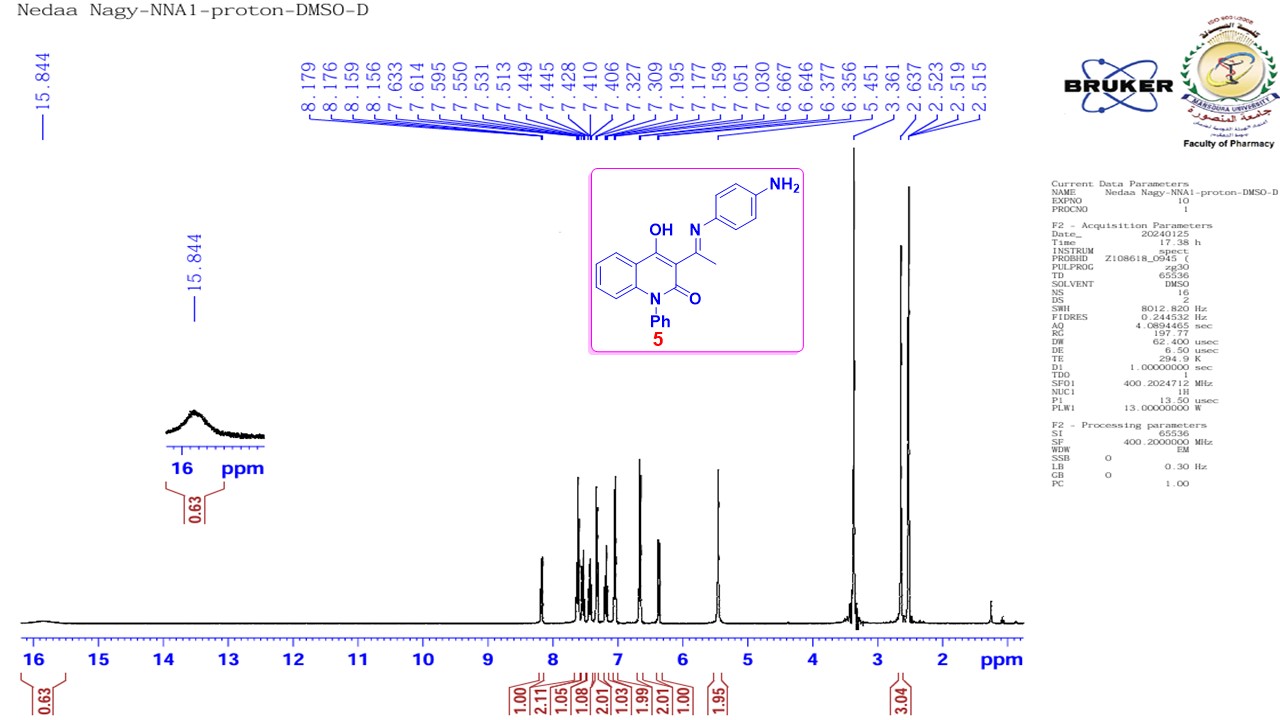
Figure 13: ^1^H NMR Spectrum of compound 5**

**
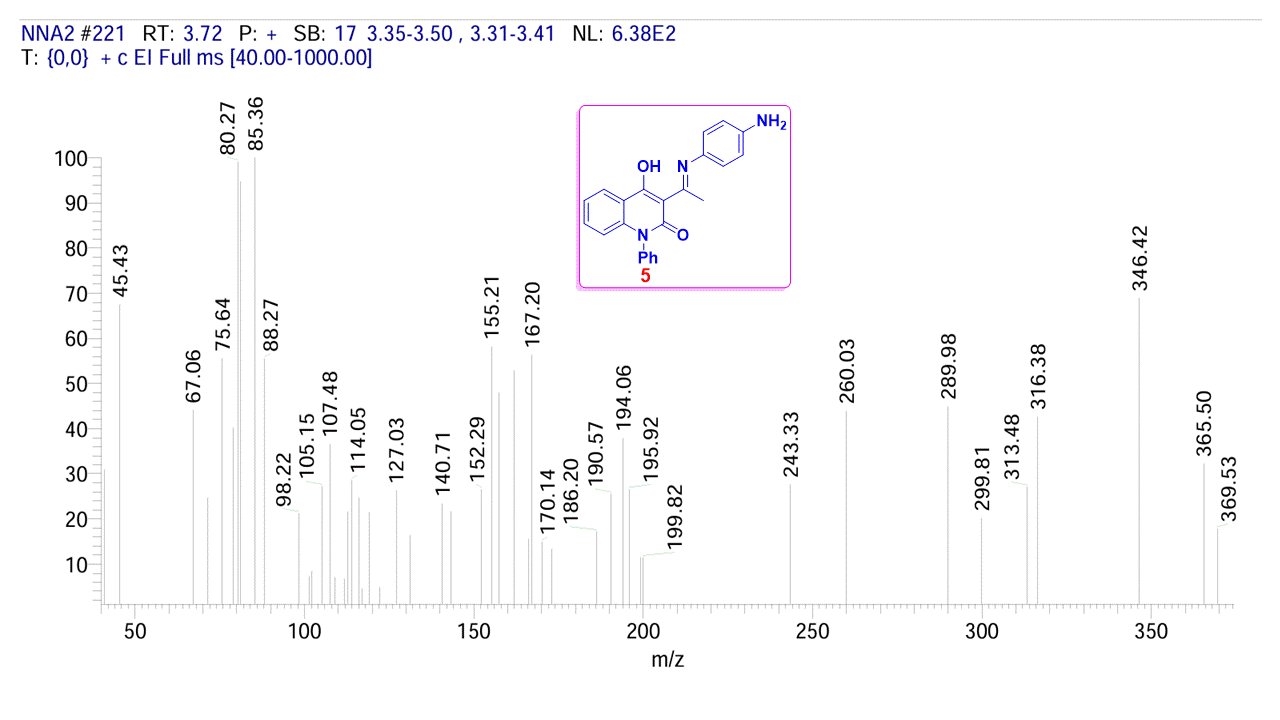
Figure 14: Mass spectrometry of compound 5**

**
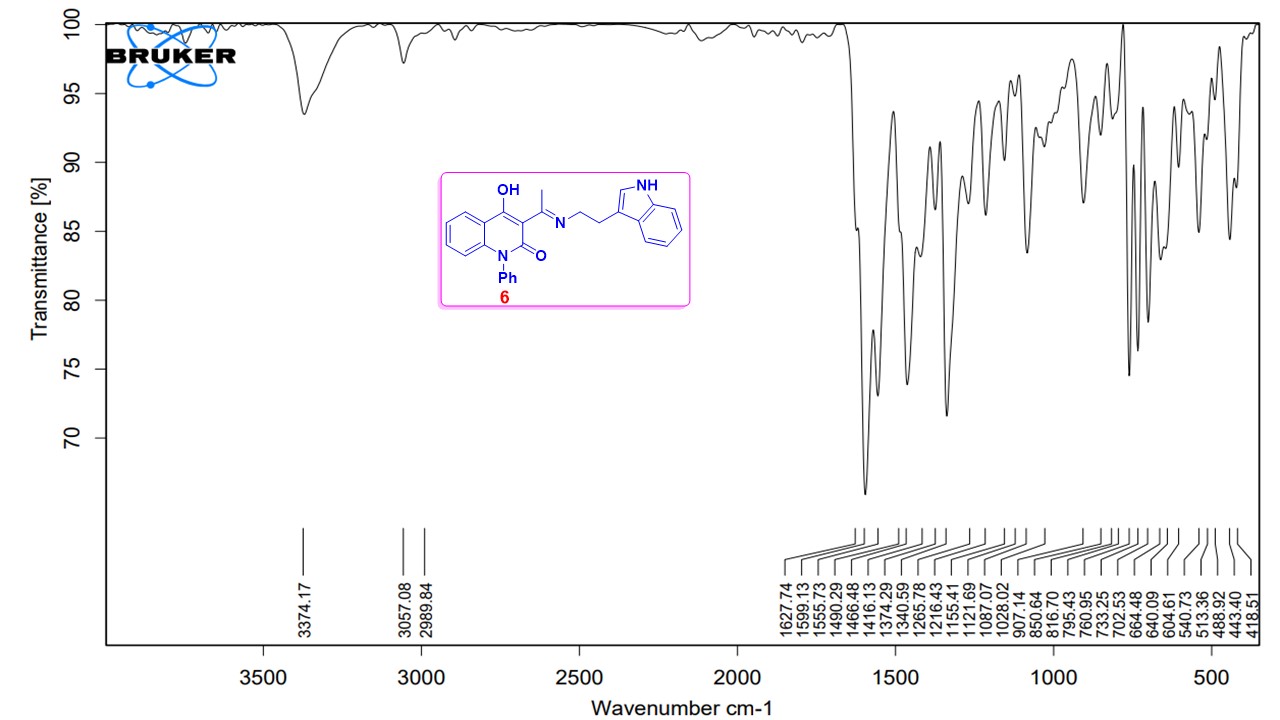
Figure 15: IR Spectrum of compound 6**

**
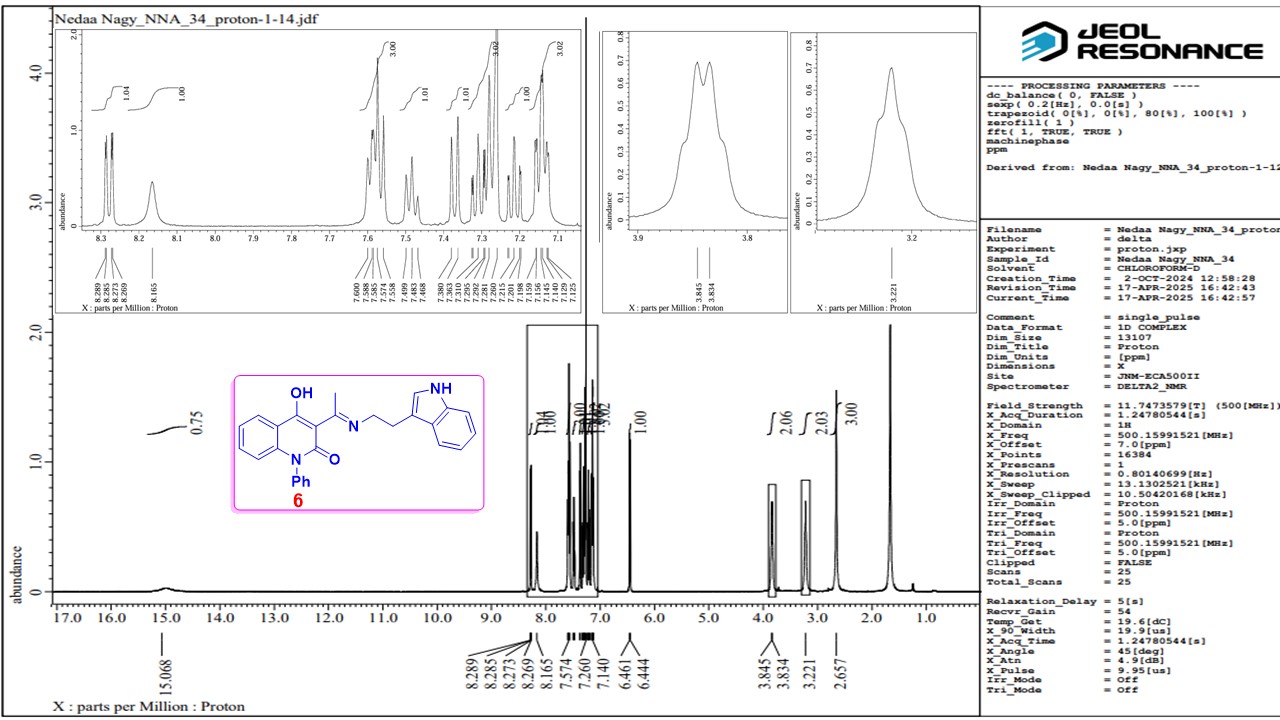
Figure 16: ^1^H NMR Spectrum of compound 6**

**
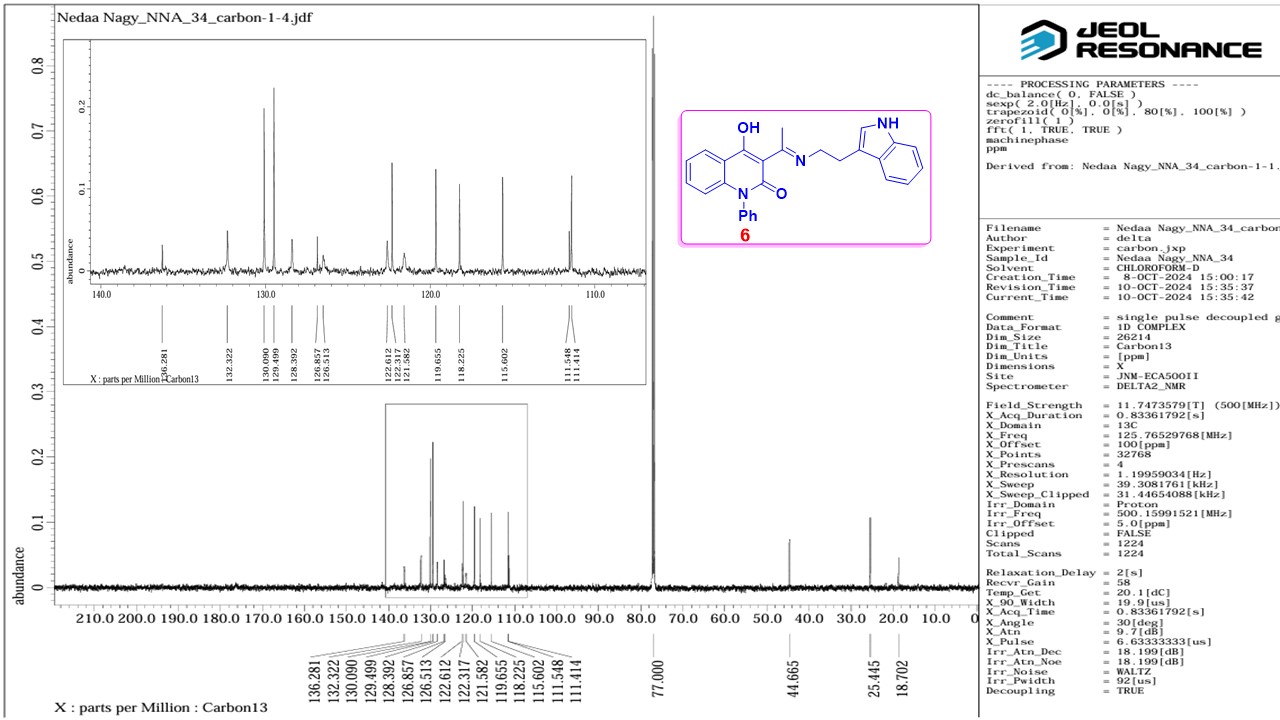
Figure 17: ^13^C NMR Spectrum of compound 6**

**
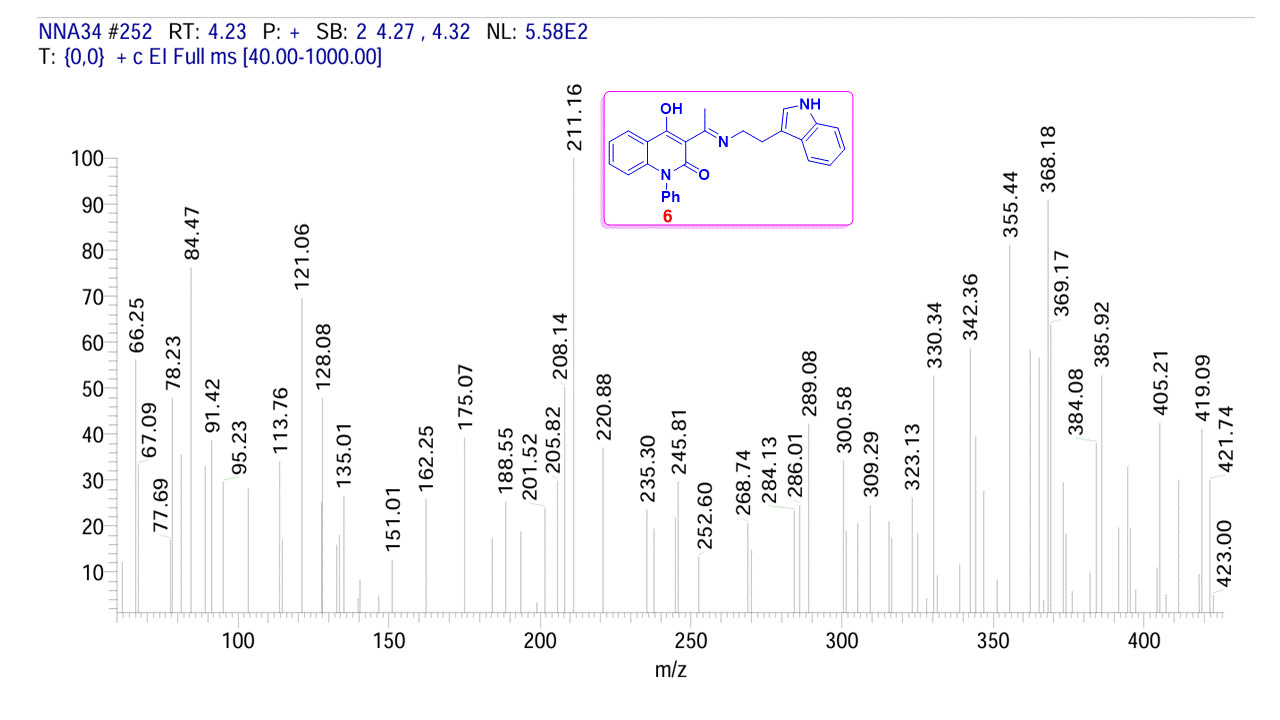
Figure 18: Mass spectrometry of compound 6**

**
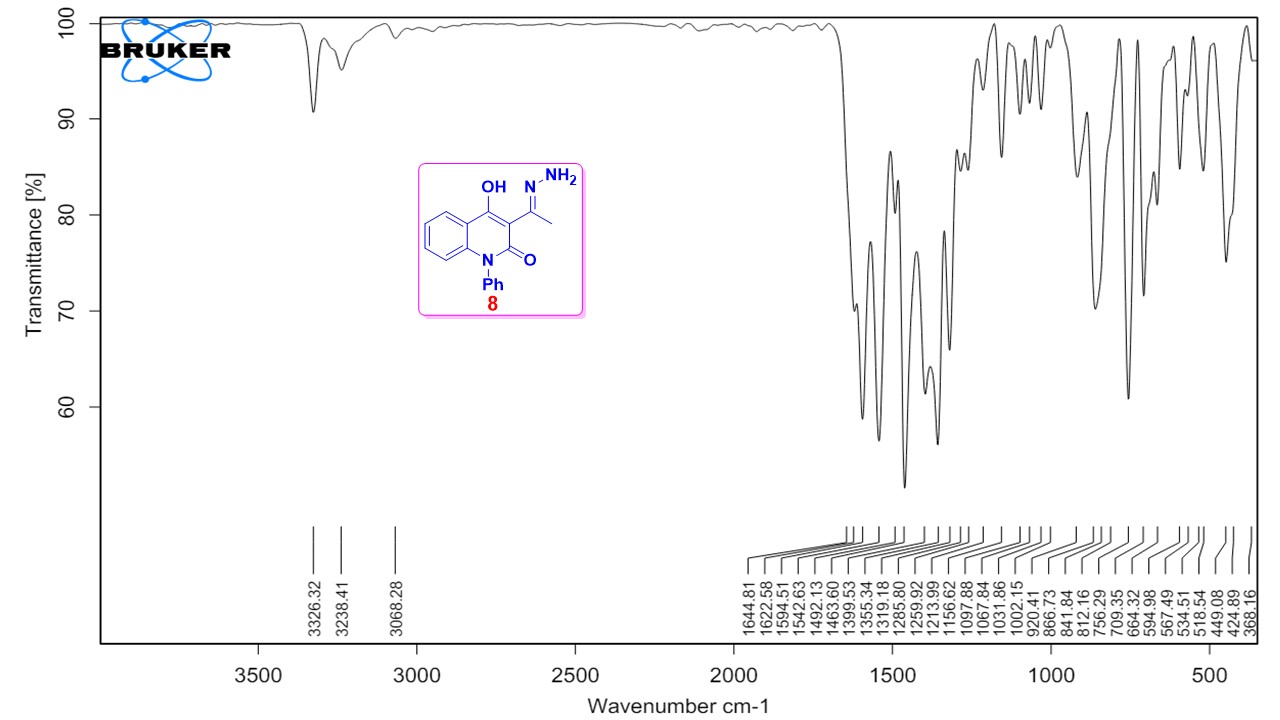
Figure 19: IR Spectrum of compound 8**

**
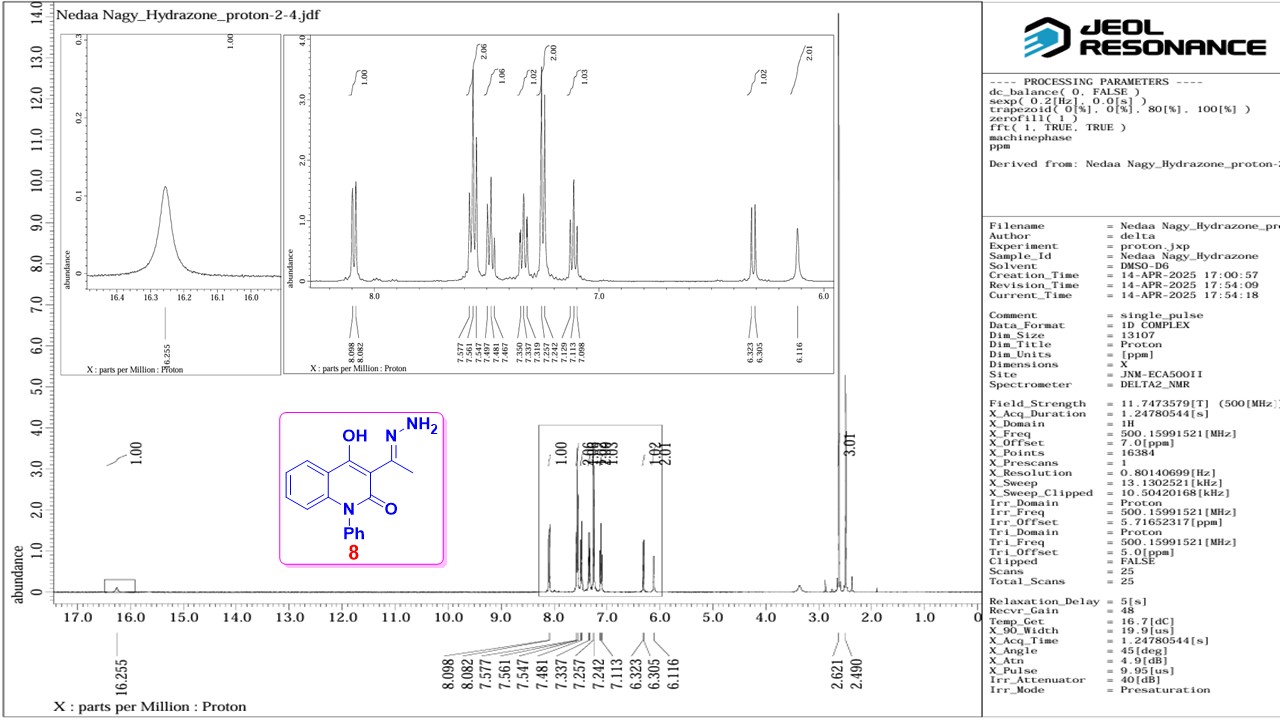
Figure 20: ^1^H NMR Spectrum of compound 8**

**
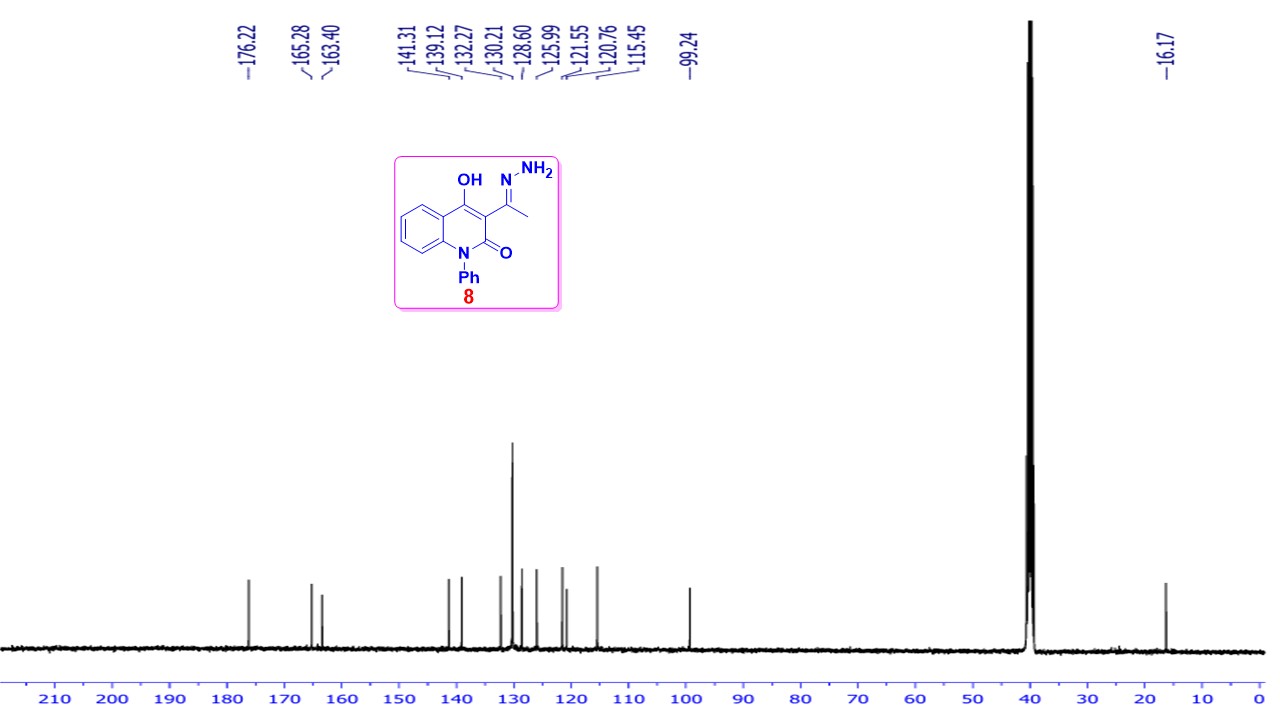
Figure 21: ^13^C NMR Spectrum of compound 8**

**
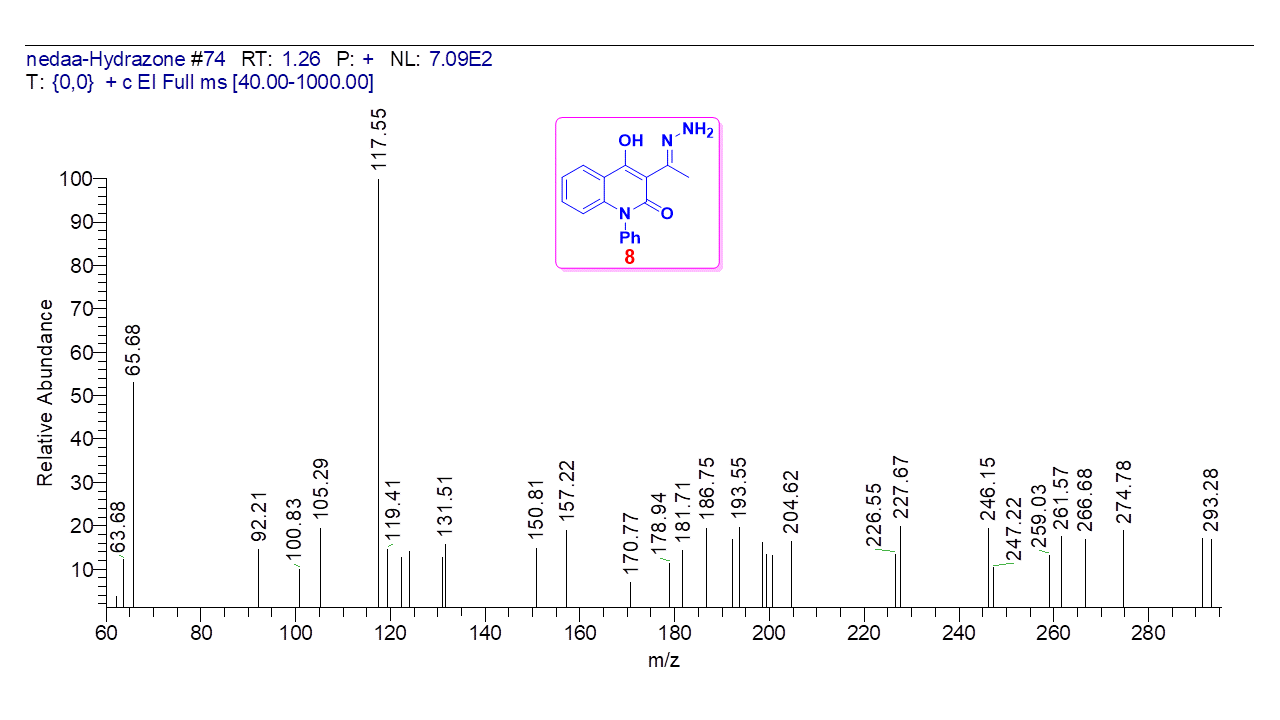
Figure 22: Mass spectrometry of compound 8**

**
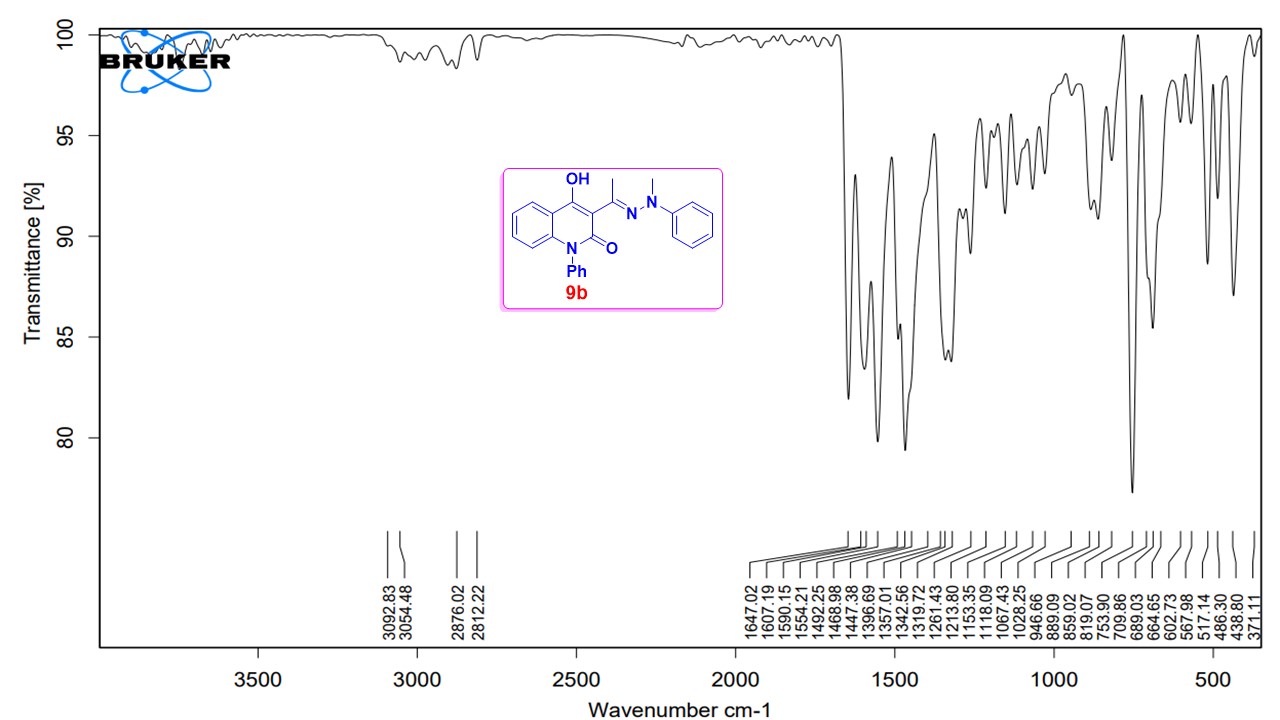
 Figure 23: IR Spectrum of compound 9b**

**
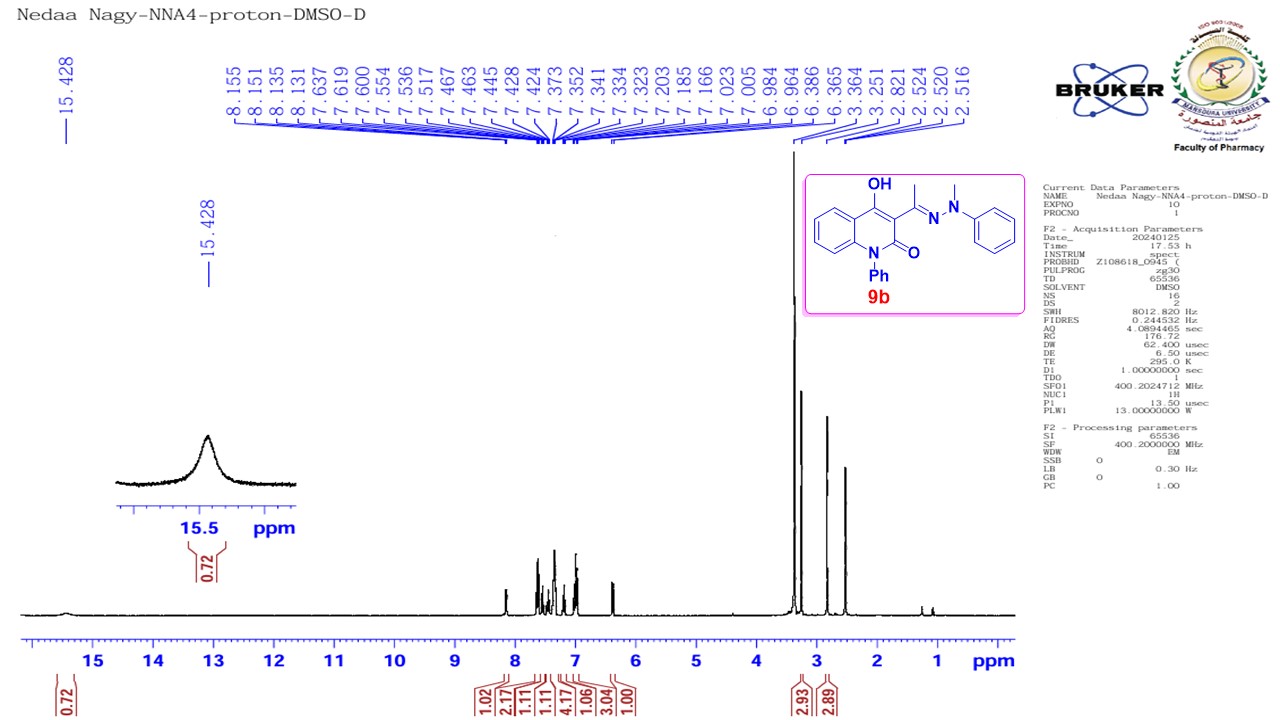
Figure 24: ^1^H NMR Spectrum of compound 9b**

**
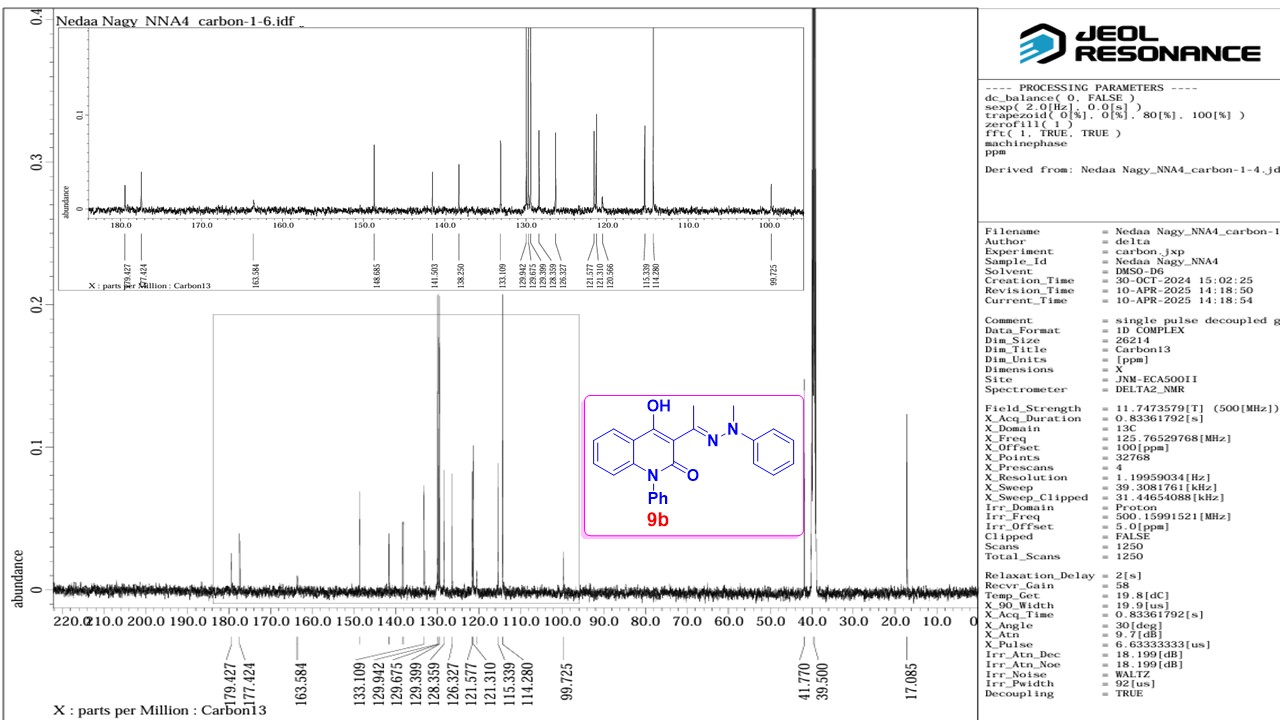
Figure 25: ^13^C NMR Spectrum of compound 9b**

**
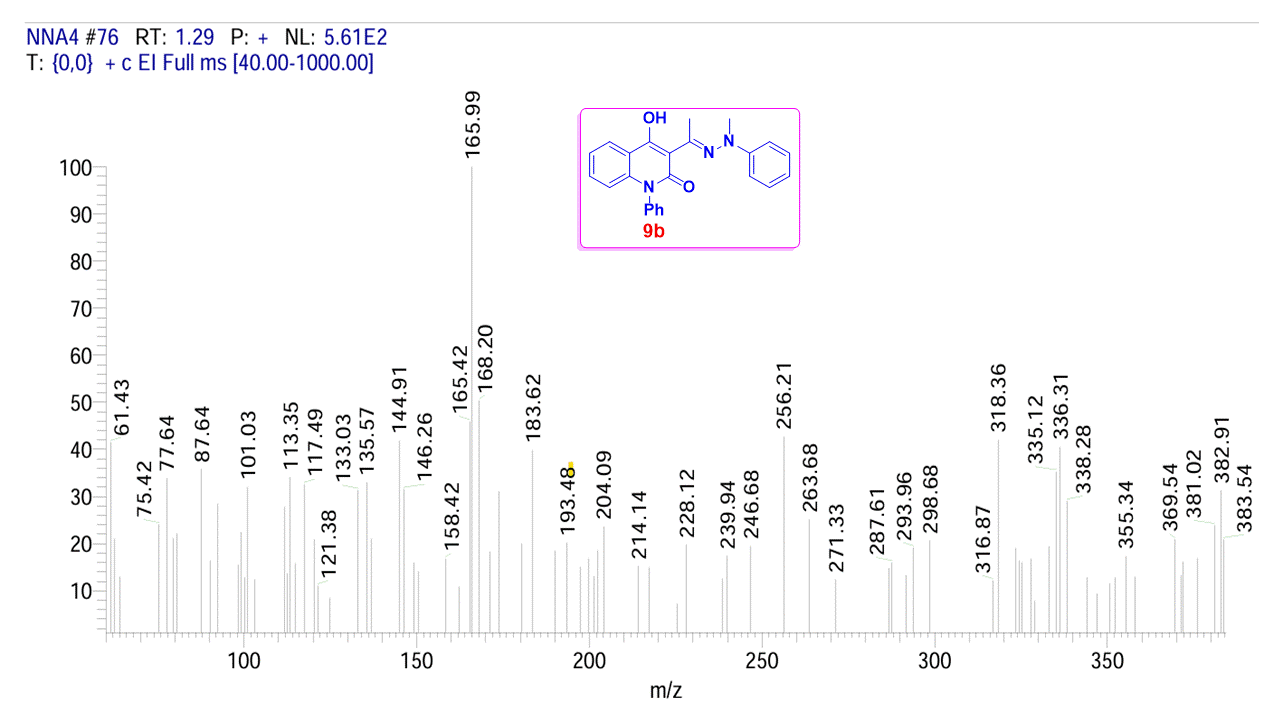
Figure 26: Mass spectrometry of compound 9b**

**
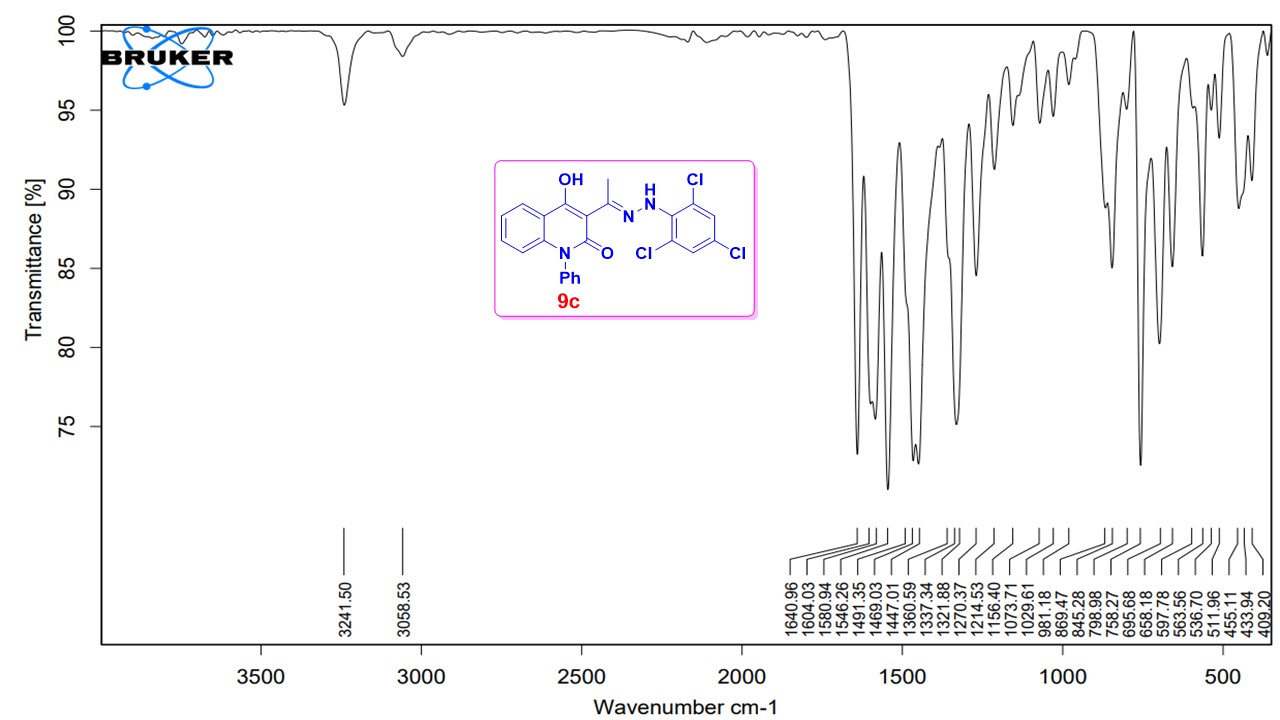
Figure 27: IR Spectrum of compound 9c**

**
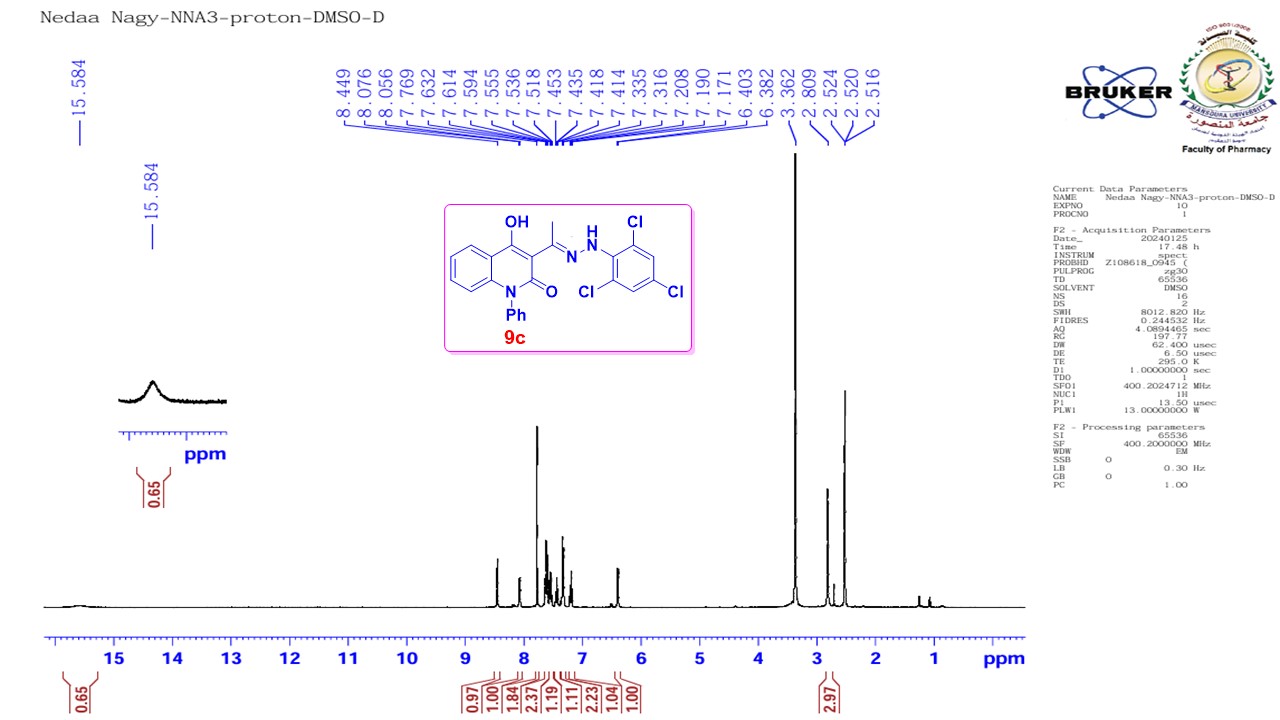
Figure 28: ^1^H NMR Spectrum of compound 9c**

**
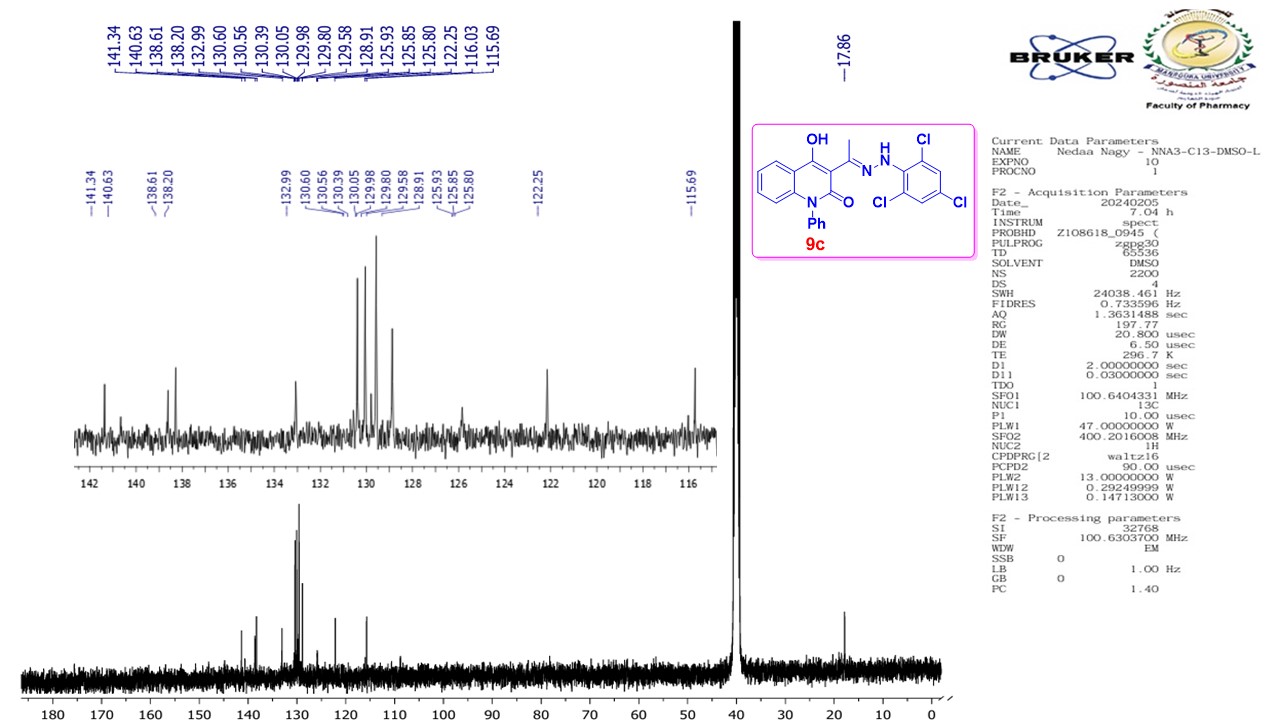
Figure 29: ^13^C NMR Spectrum of compound 9c**

**
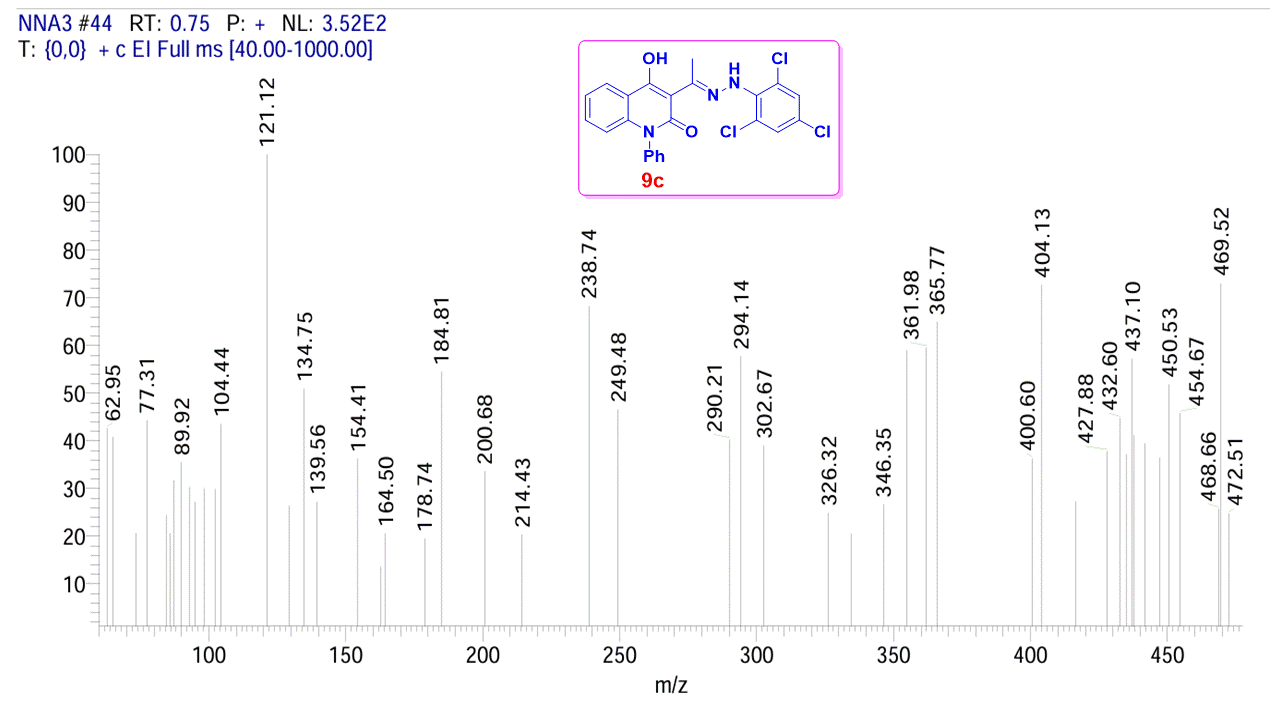
Figure 30: Mass spectrometry of compound 9c**

**
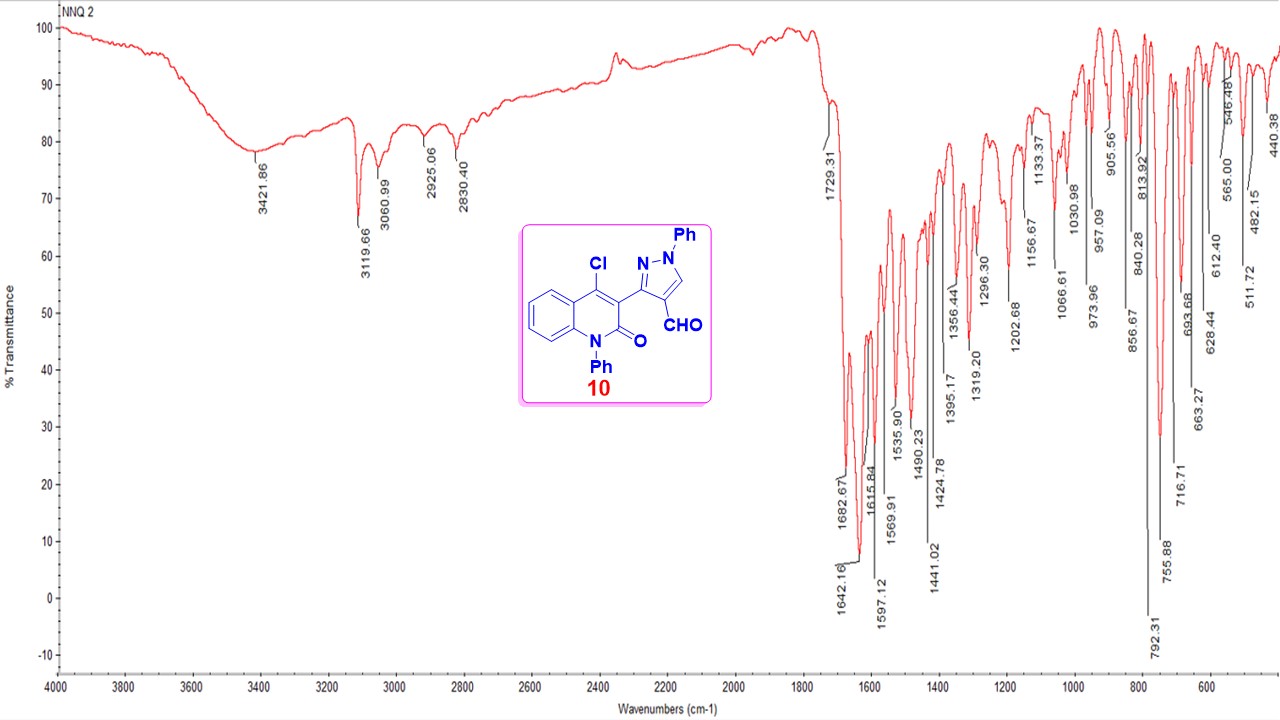
Figure 31: IR Spectrum of compound 10**

**
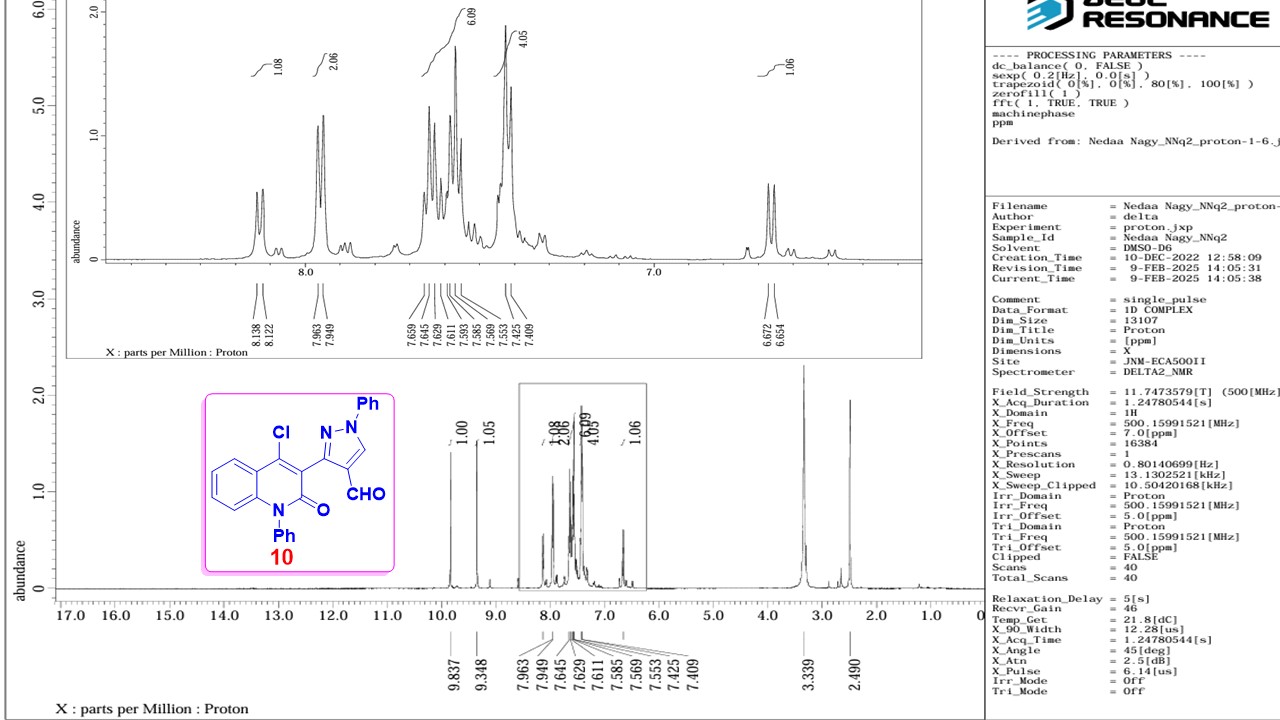
Figure 32: ^1^H NMR Spectrum of compound 10**

**
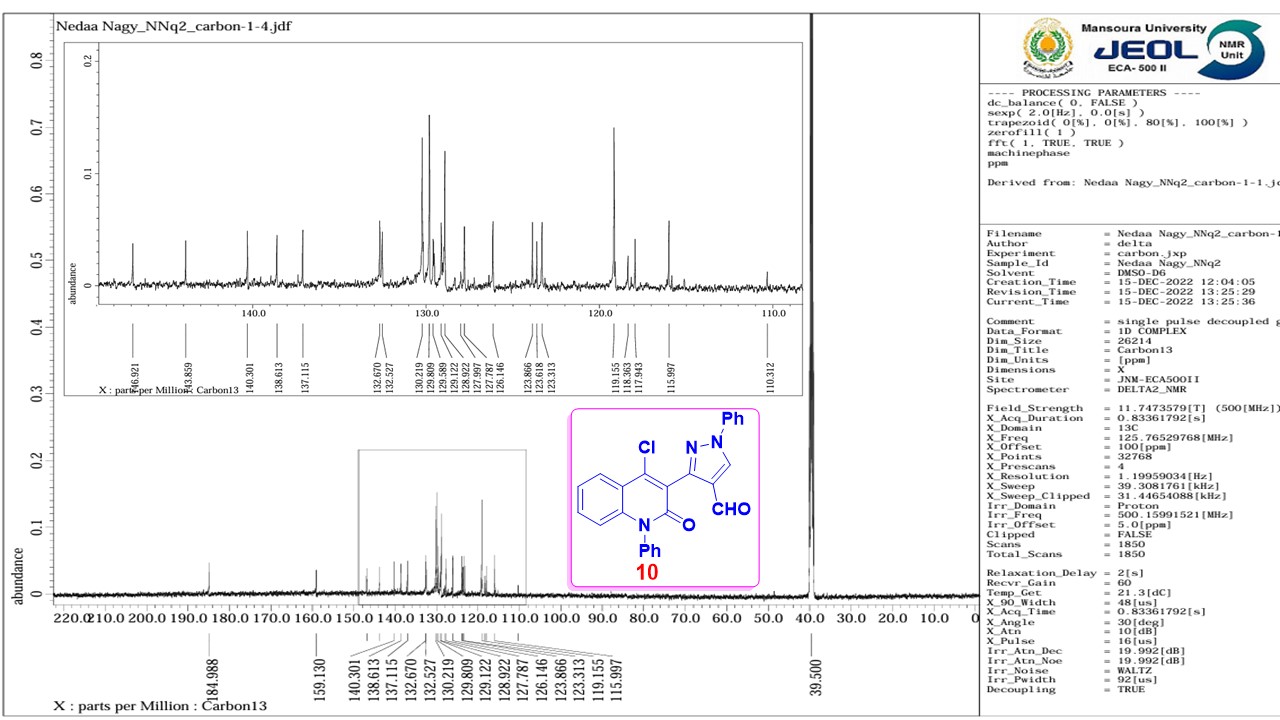
Figure 33: ^13^C NMR Spectrum of compound 10**

**
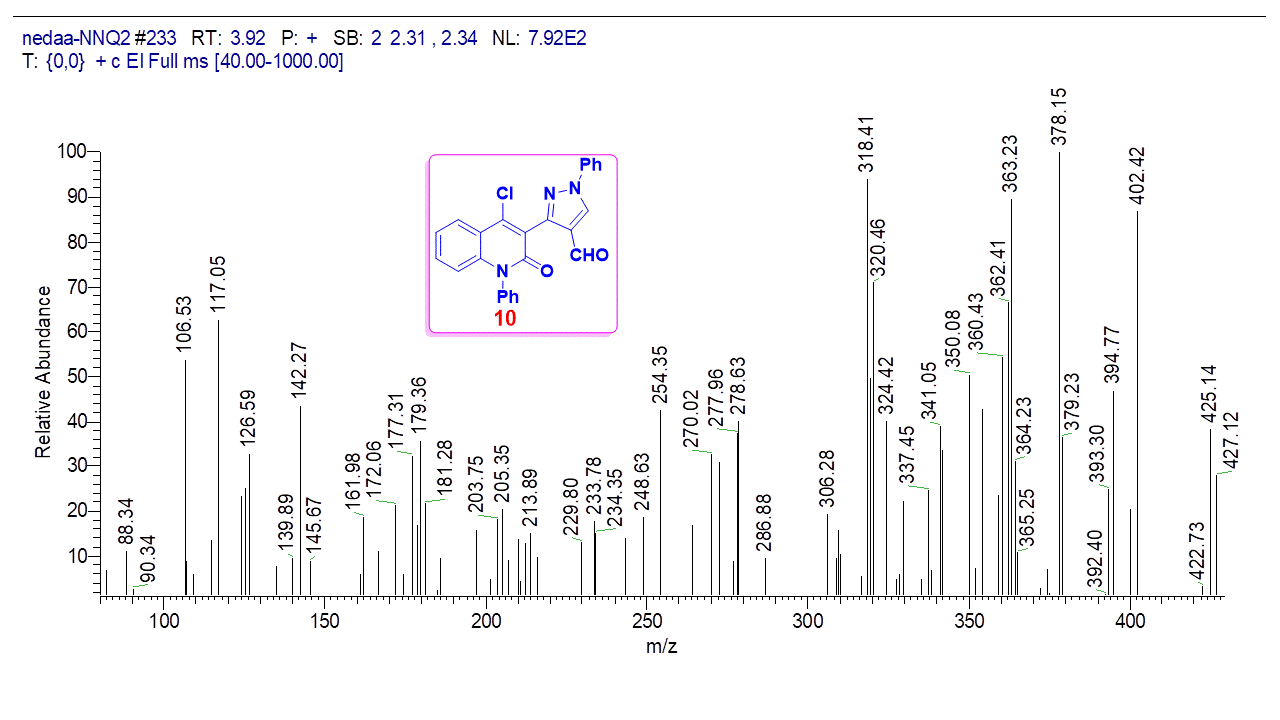
Figure 34: Mass spectrometry of compound 10**

**
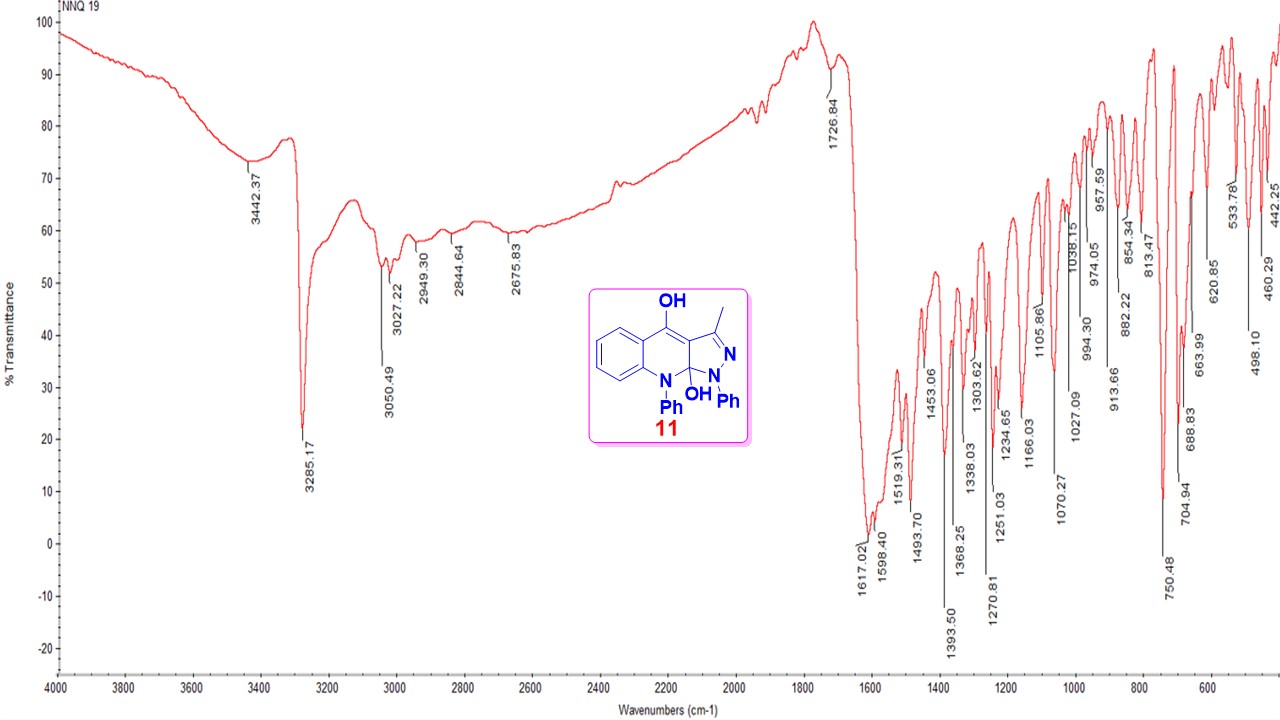
Figure 35: IR Spectrum of compound 11**

**
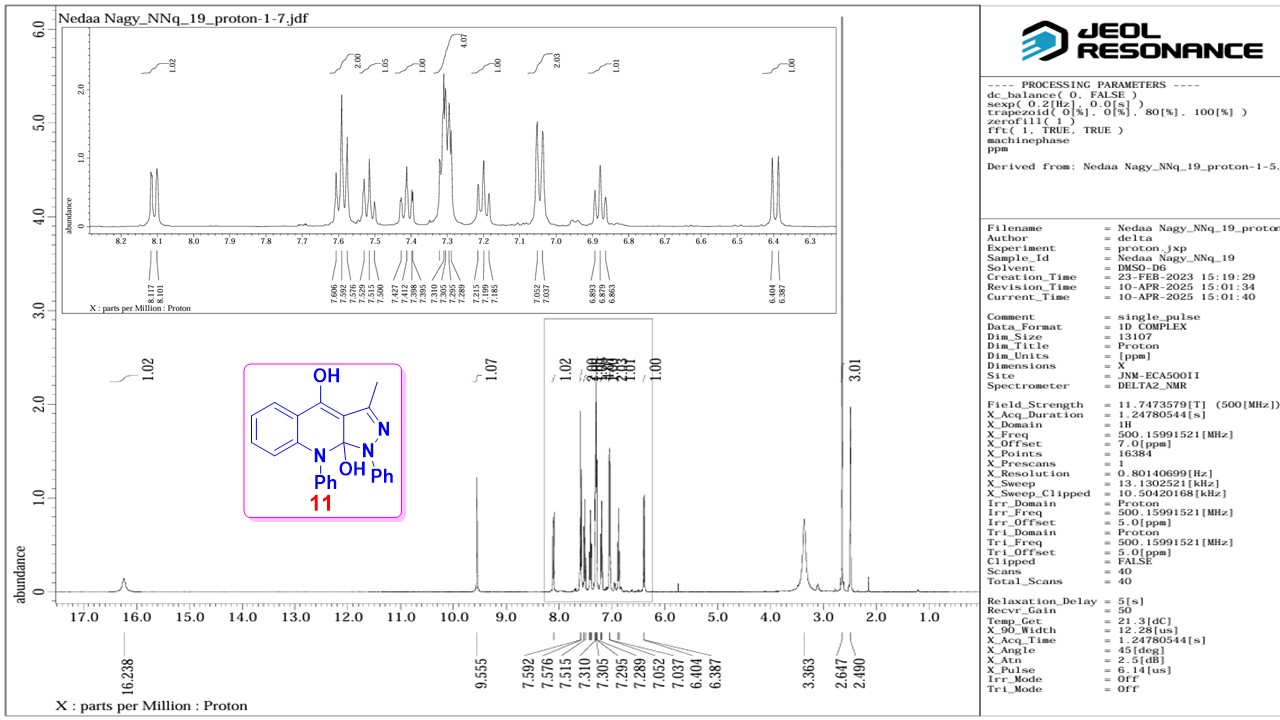
Figure 36: ^1^H NMR Spectrum of compound 11**

**
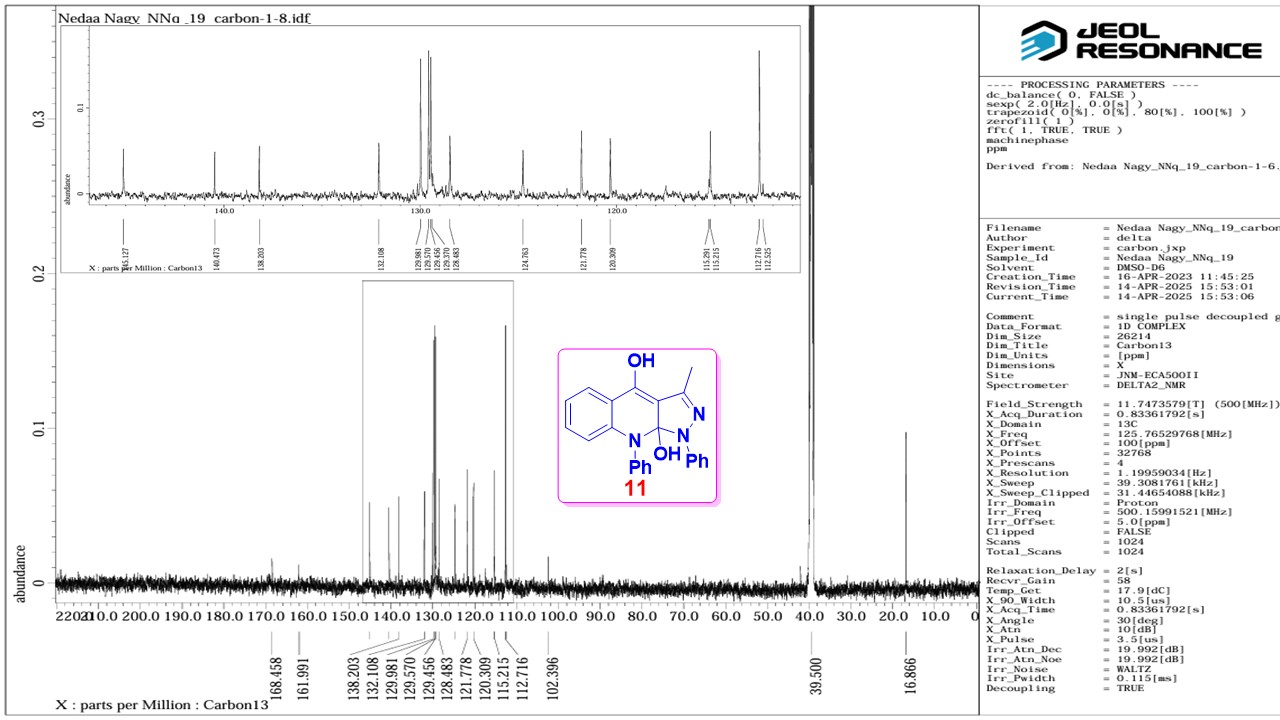
Figure 37: ^13^C NMR Spectrum of compound 11**

**
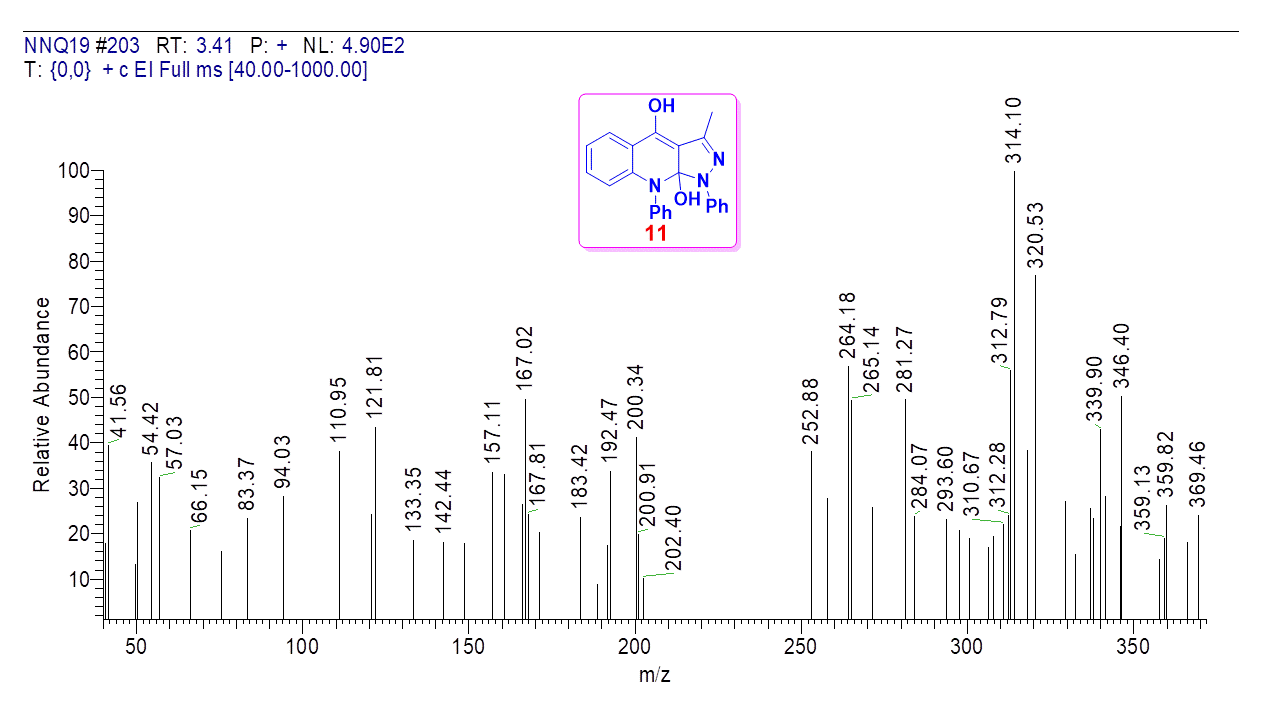
Figure 38: Mass spectrometry of compound 11**

**
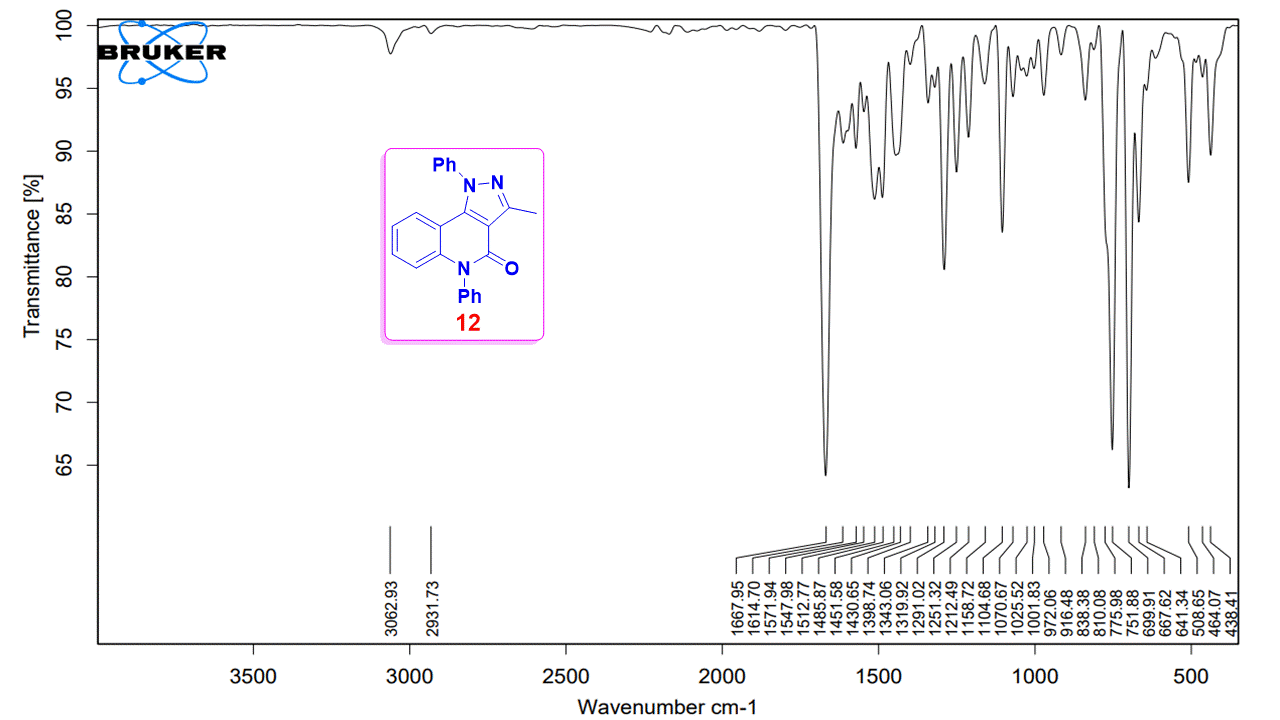
Figure 39: IR Spectrum of compound 12**

**
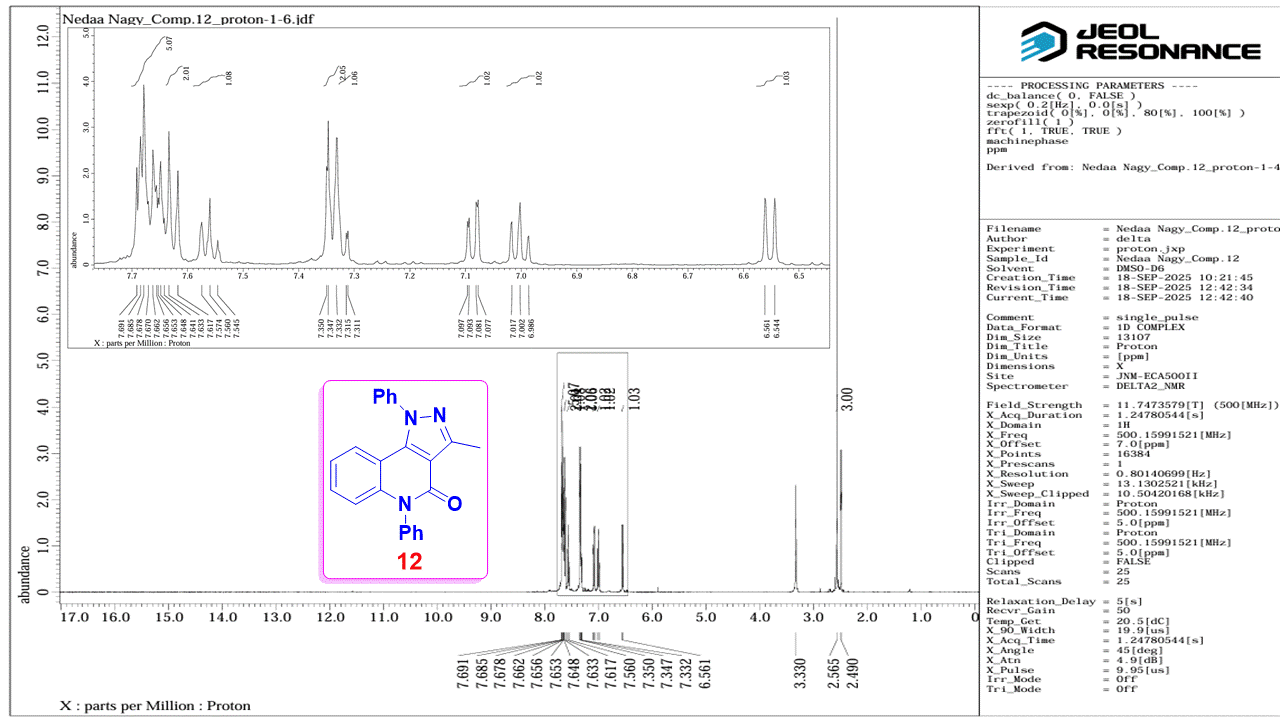
Figure 40: ^1^H NMR Spectrum of compound 12**

**
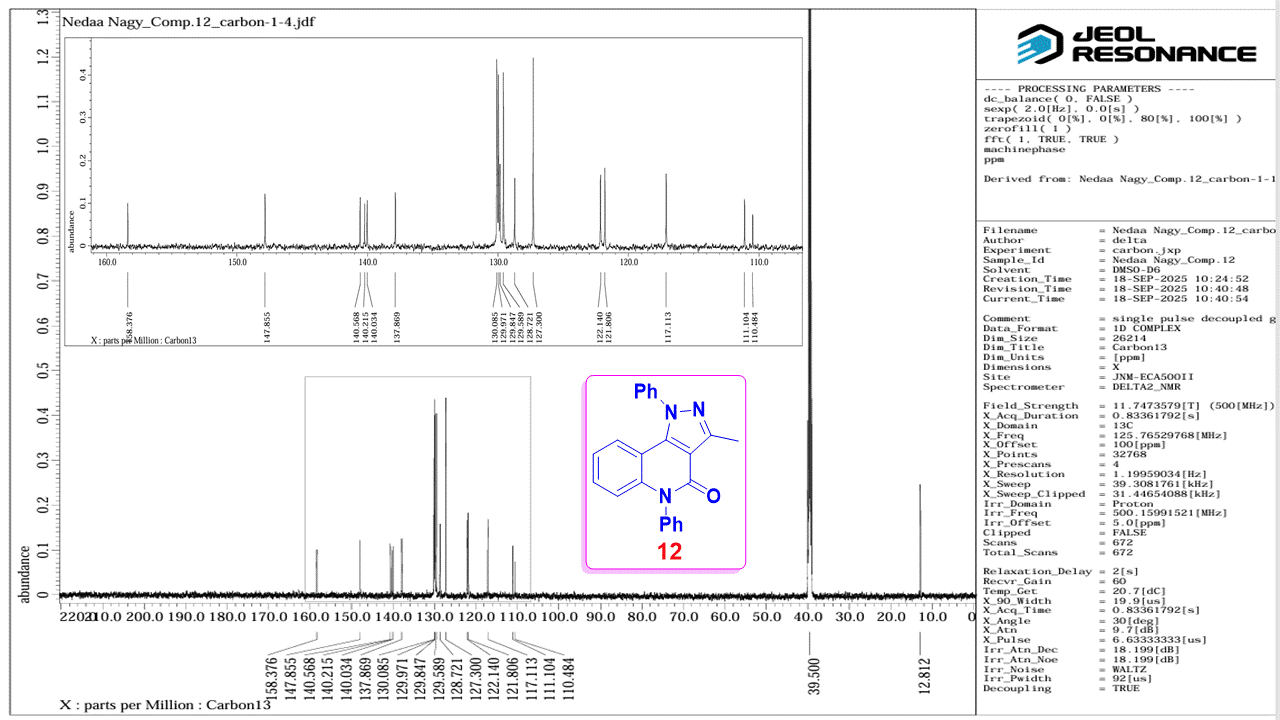
Figure 41: ^13^C NMR Spectrum of compound 12**

**
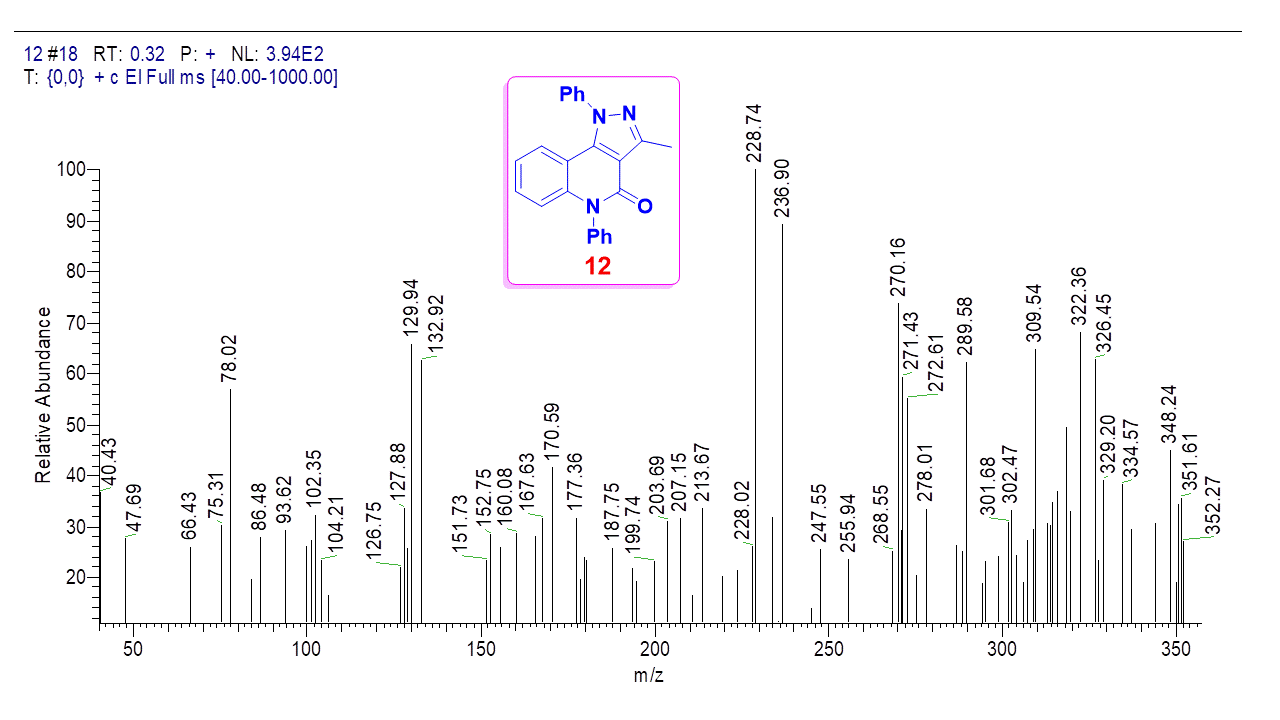
Figure 42: Mass spectrometry of compound 12**

**
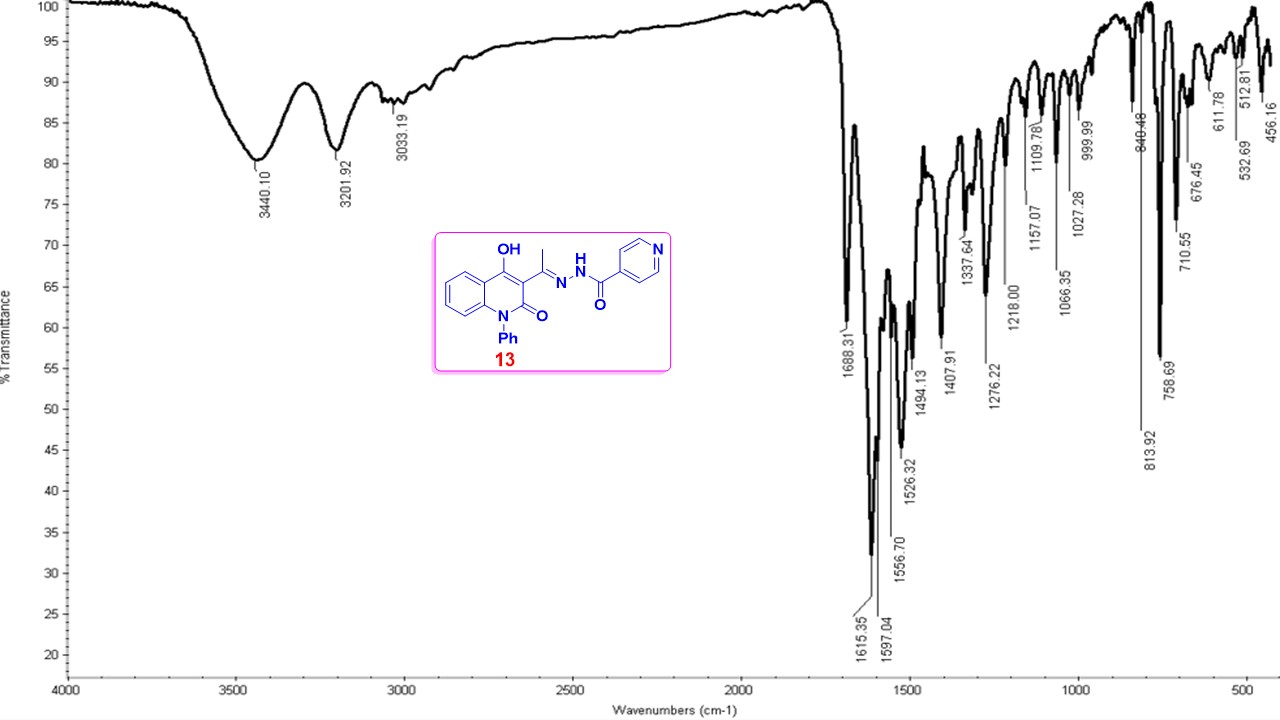
Figure 43: IR Spectrum of compound 13**

**
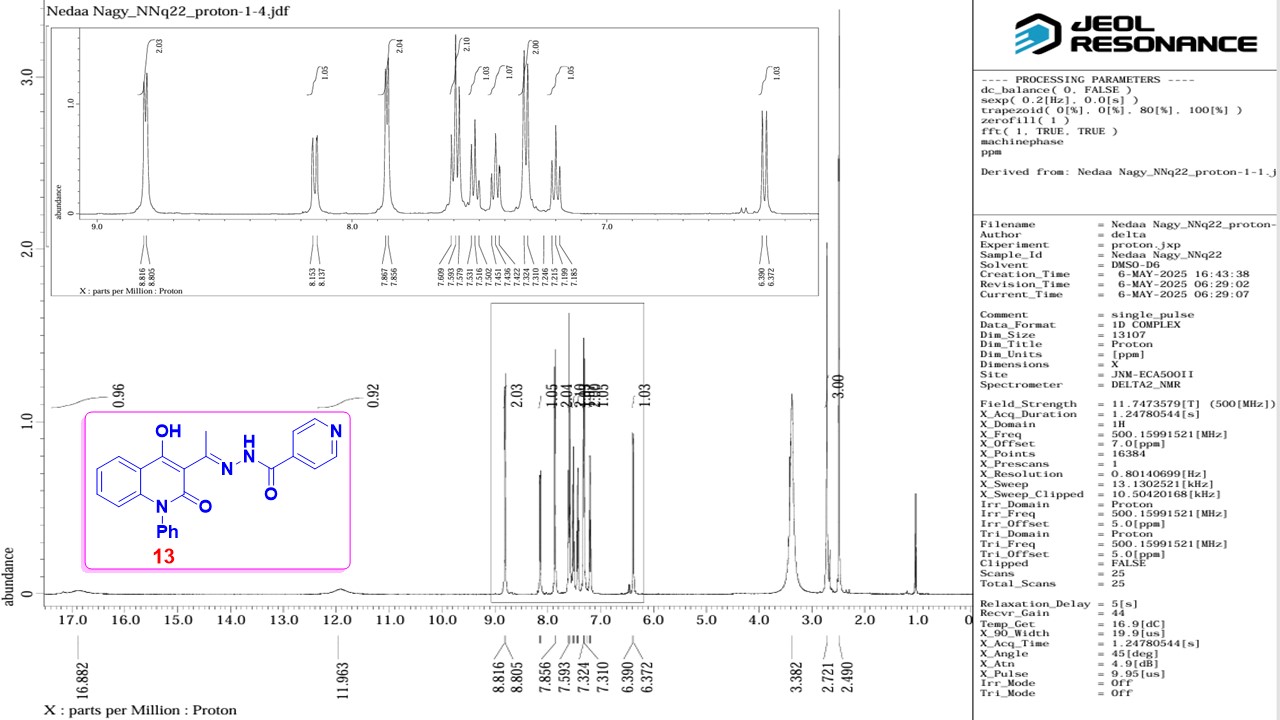
Figure 44: ^1^H NMR Spectrum of compound 13**

**
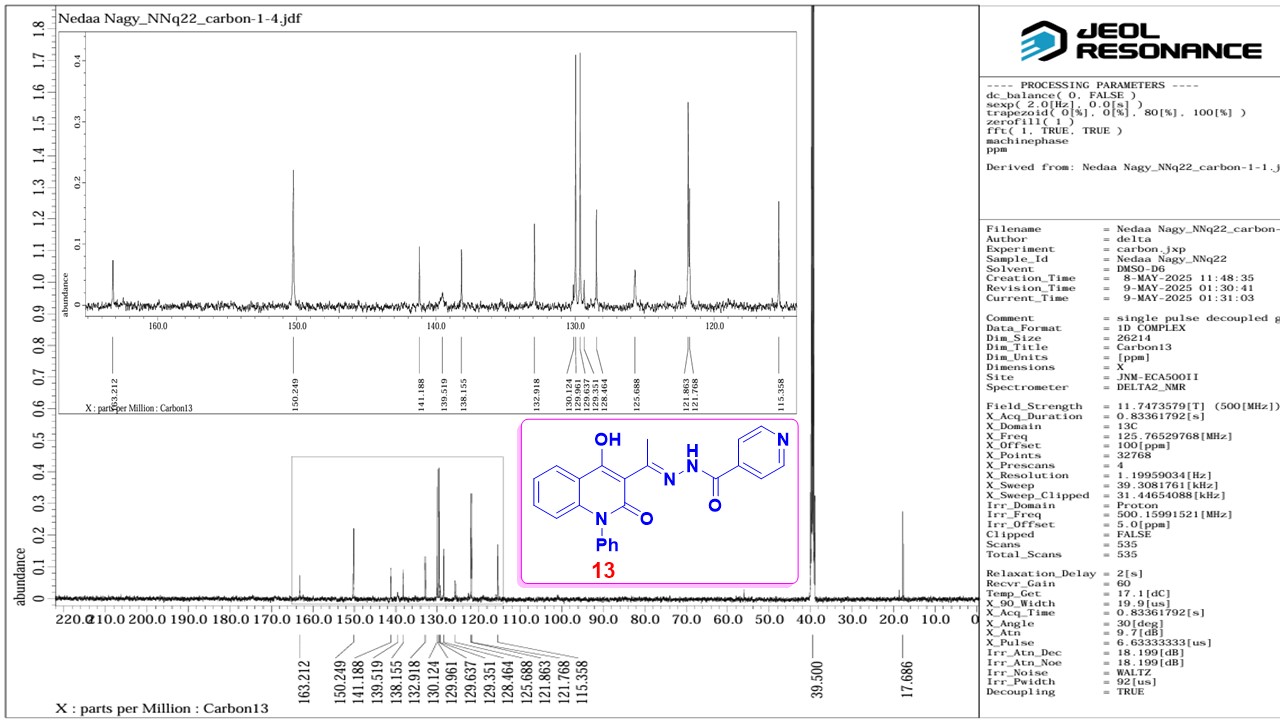
Figure 45: ^13^C NMR Spectrum of compound 13**

**
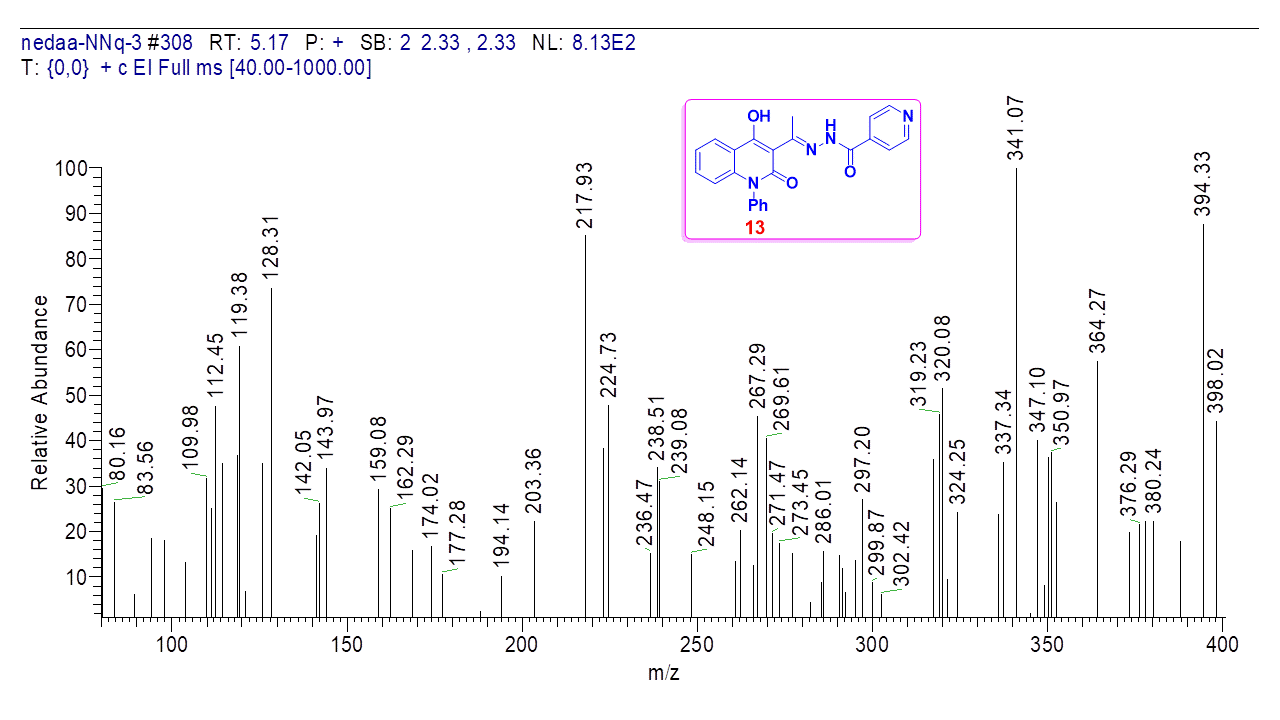
Figure 46: Mass spectrometry of compound 13**

**
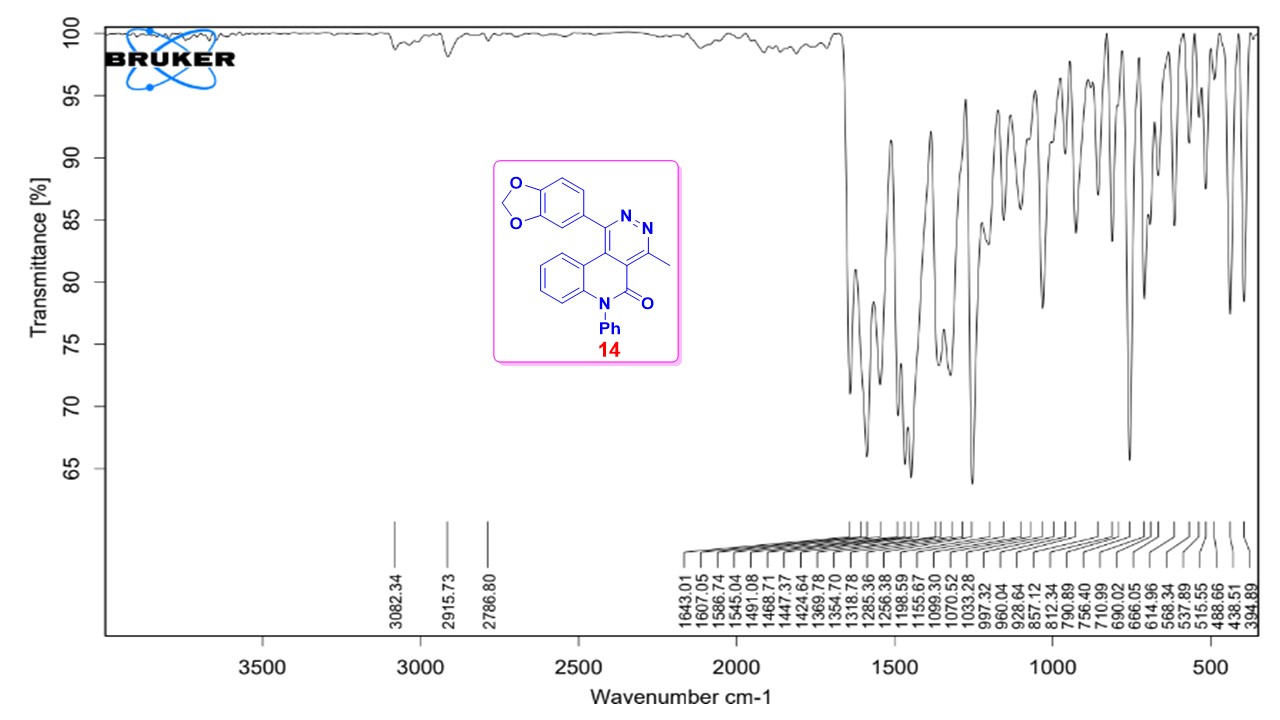
Figure 47: IR Spectrum of compound 14**

**
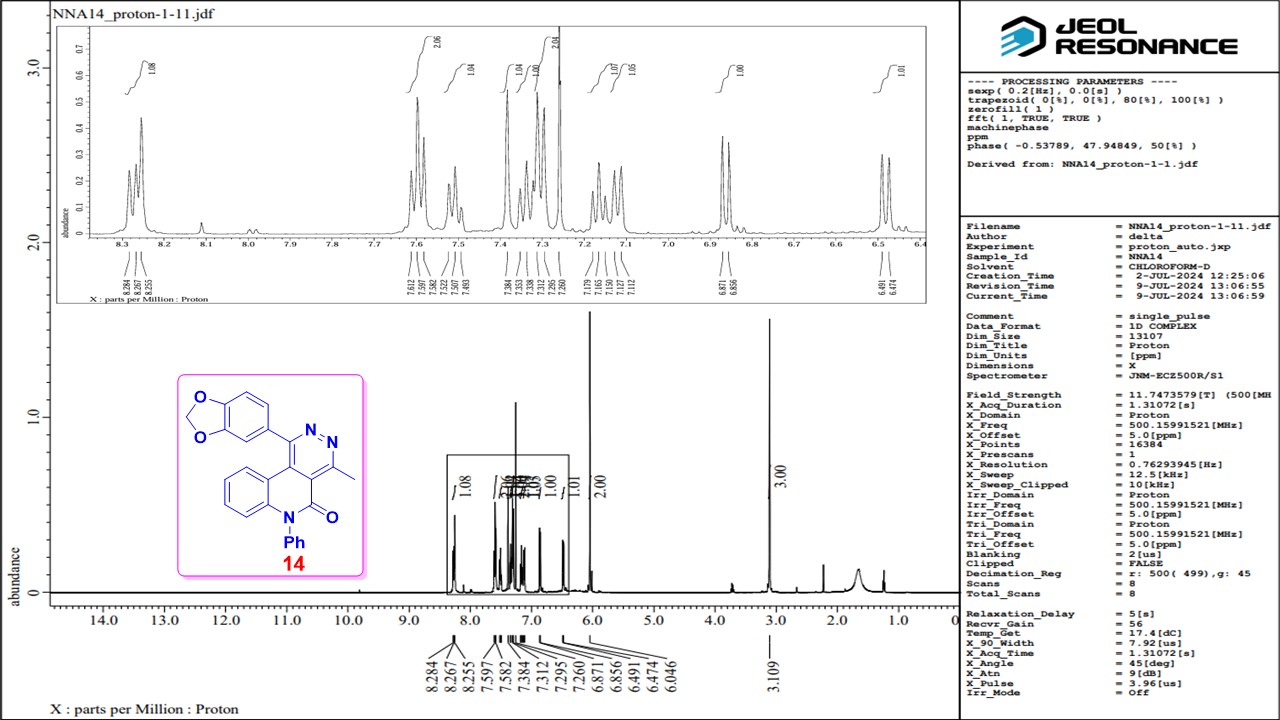
Figure 48: ^1^H NMR Spectrum of compound 14**

**
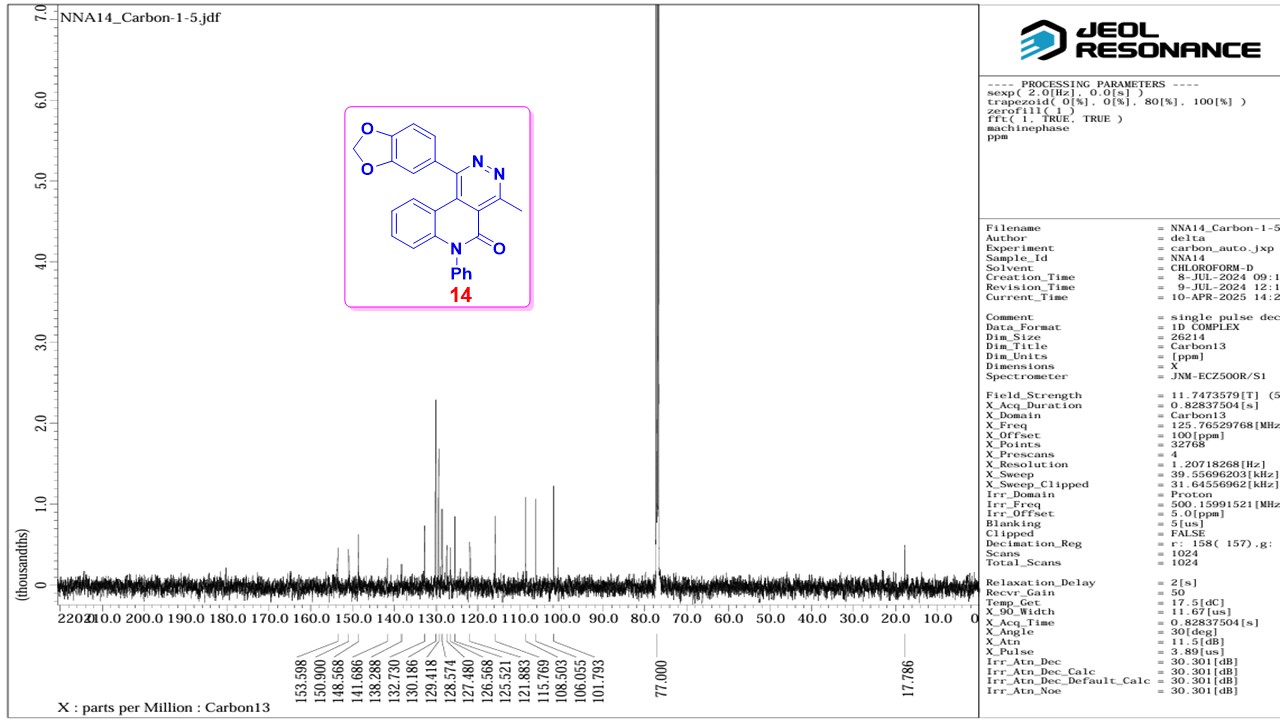
Figure 49: ^13^C NMR Spectrum of compound 14**

**
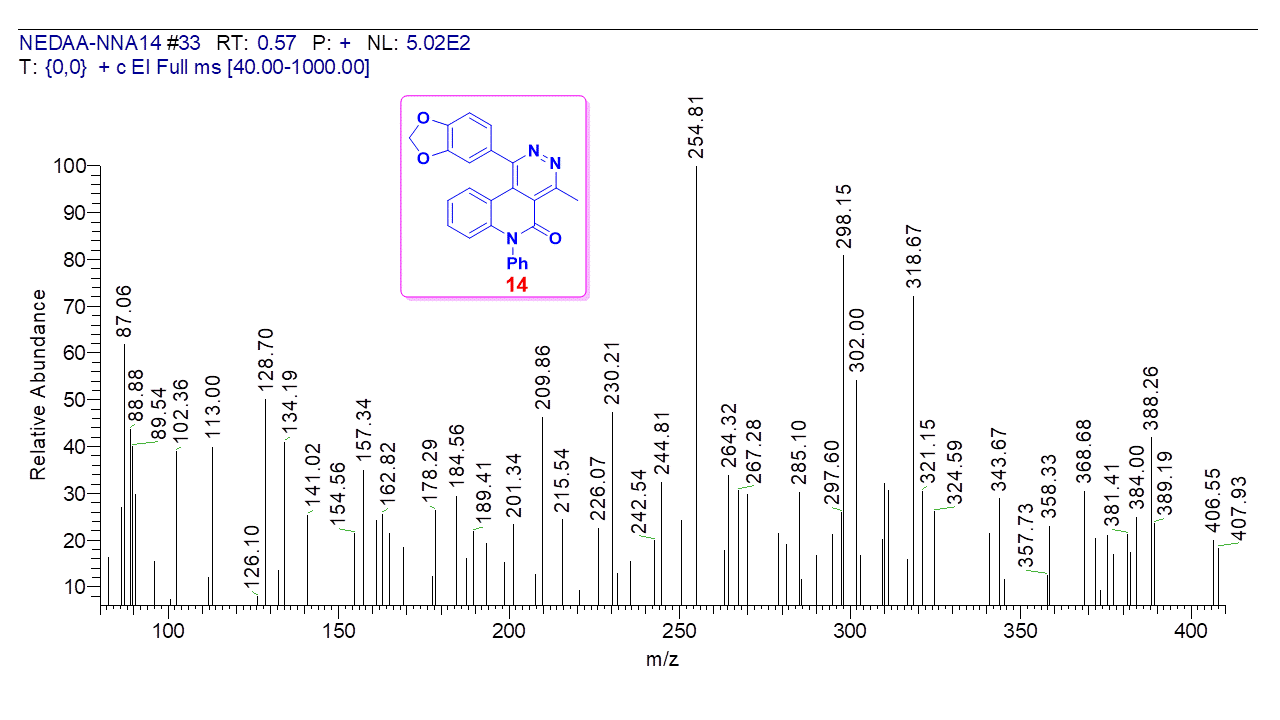
Figure 50: Mass spectrometry of compound 14**

**
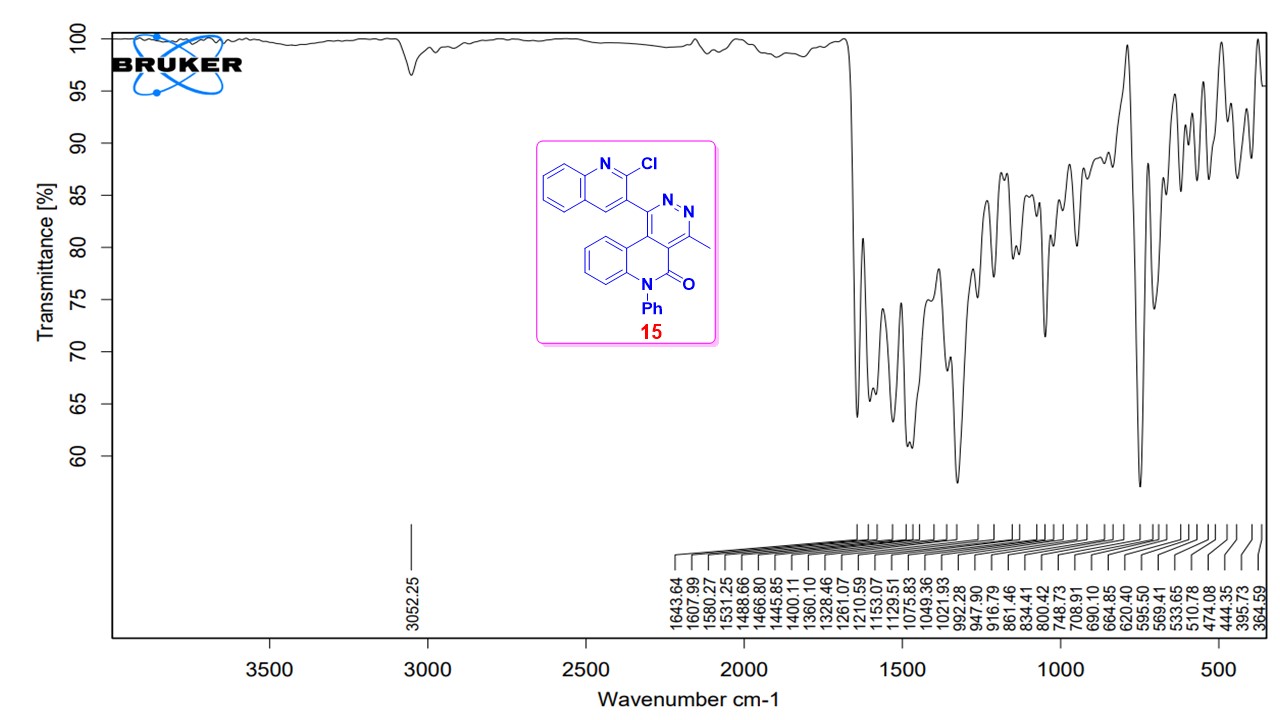
Figure 51: IR Spectrum of compound 15**

**
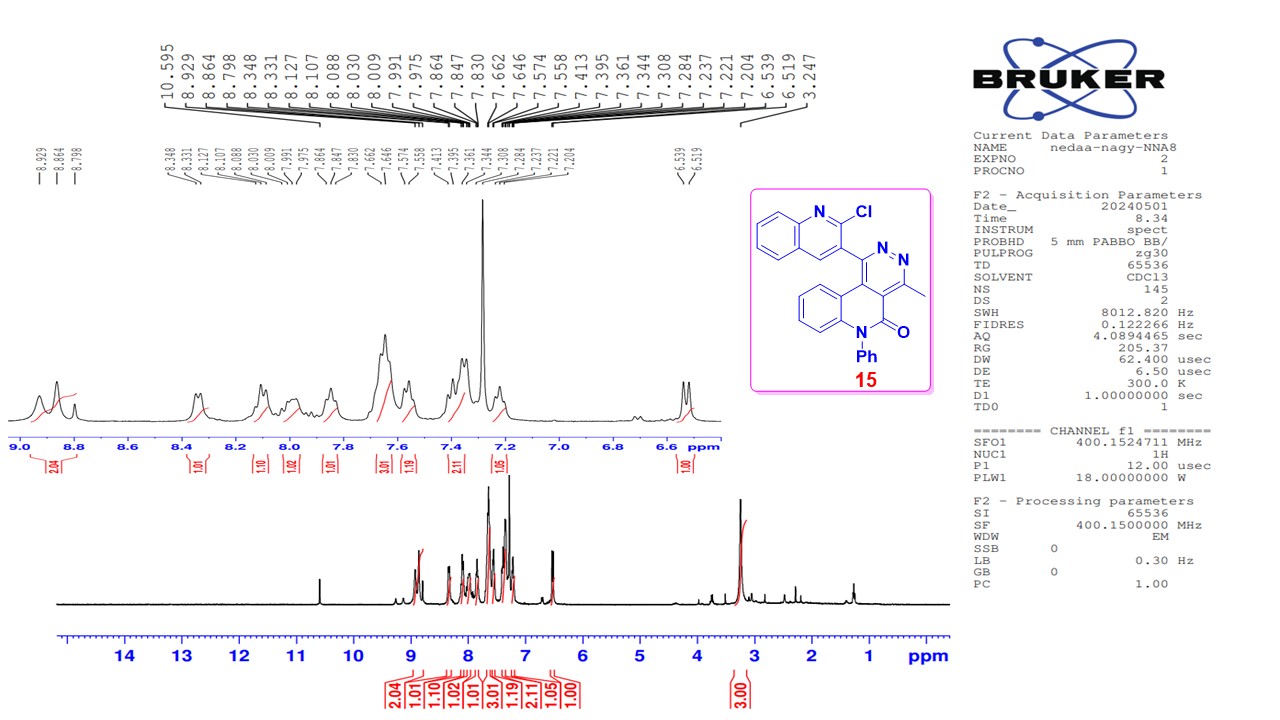
Figure 52: ^1^H NMR Spectrum of compound 15**

**
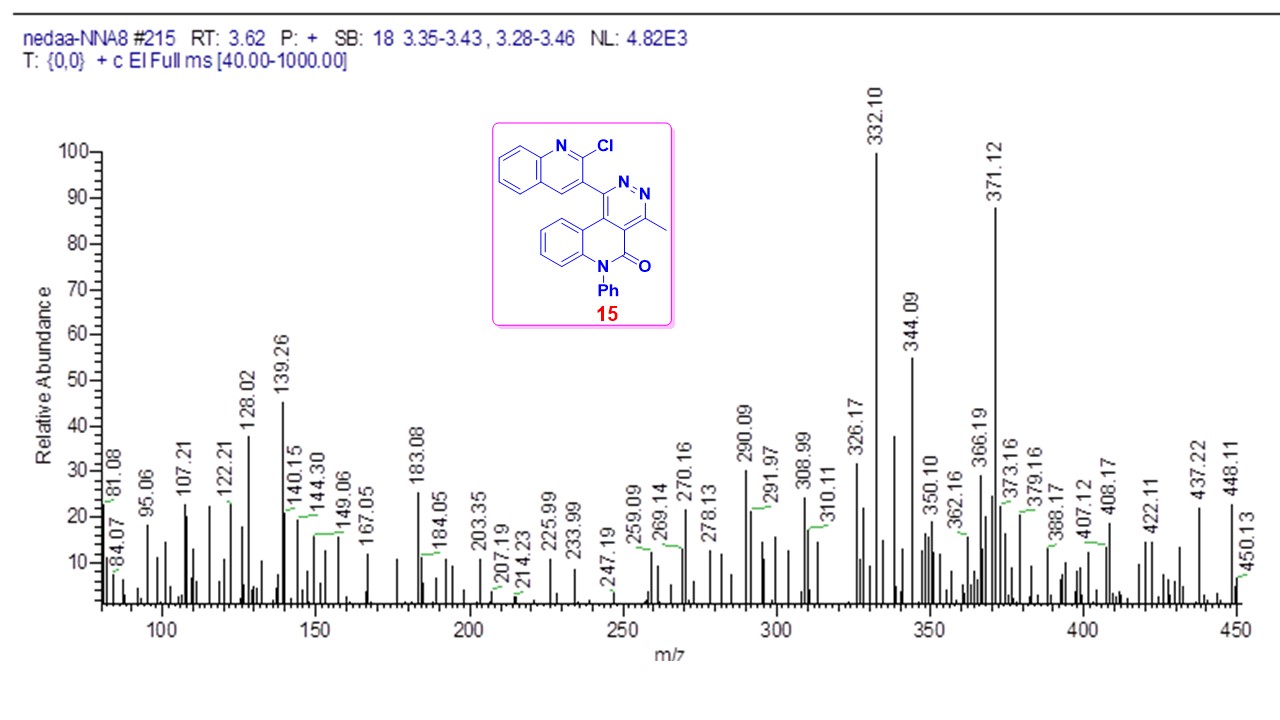
Figure 53: Mass spectrometry of compound 15**

**
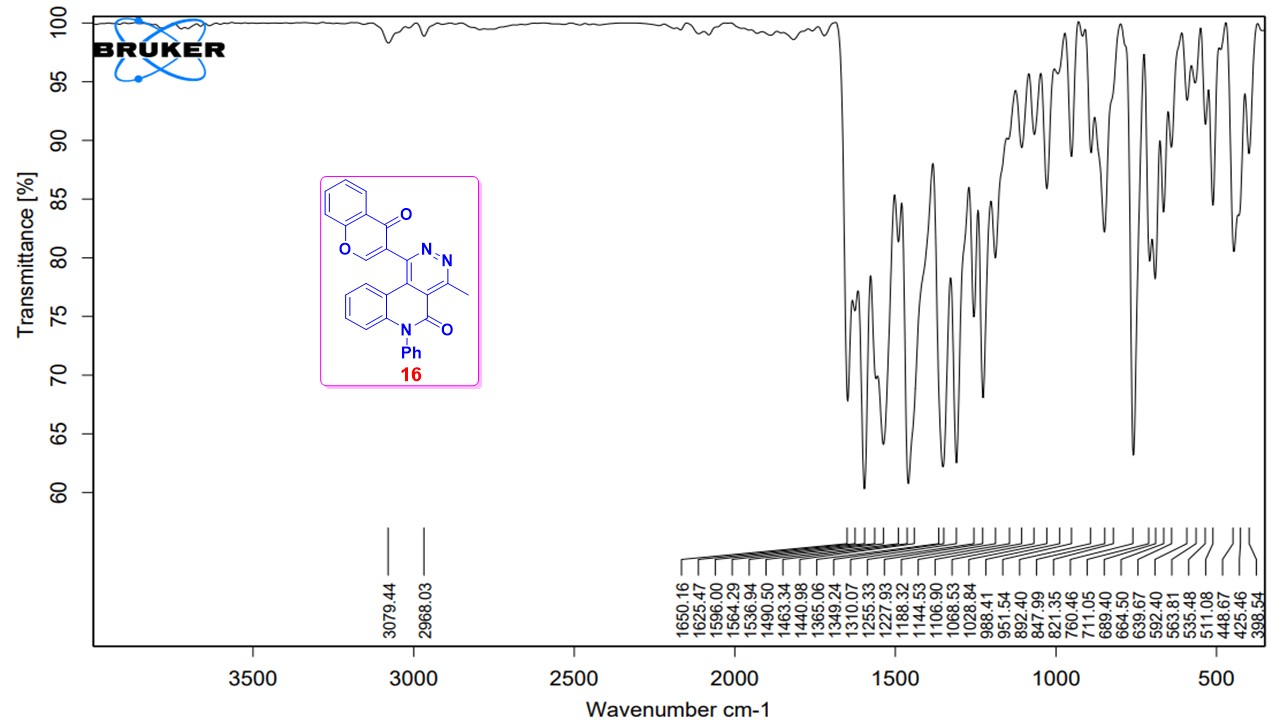
Figure 54: IR Spectrum of compound 16**

**
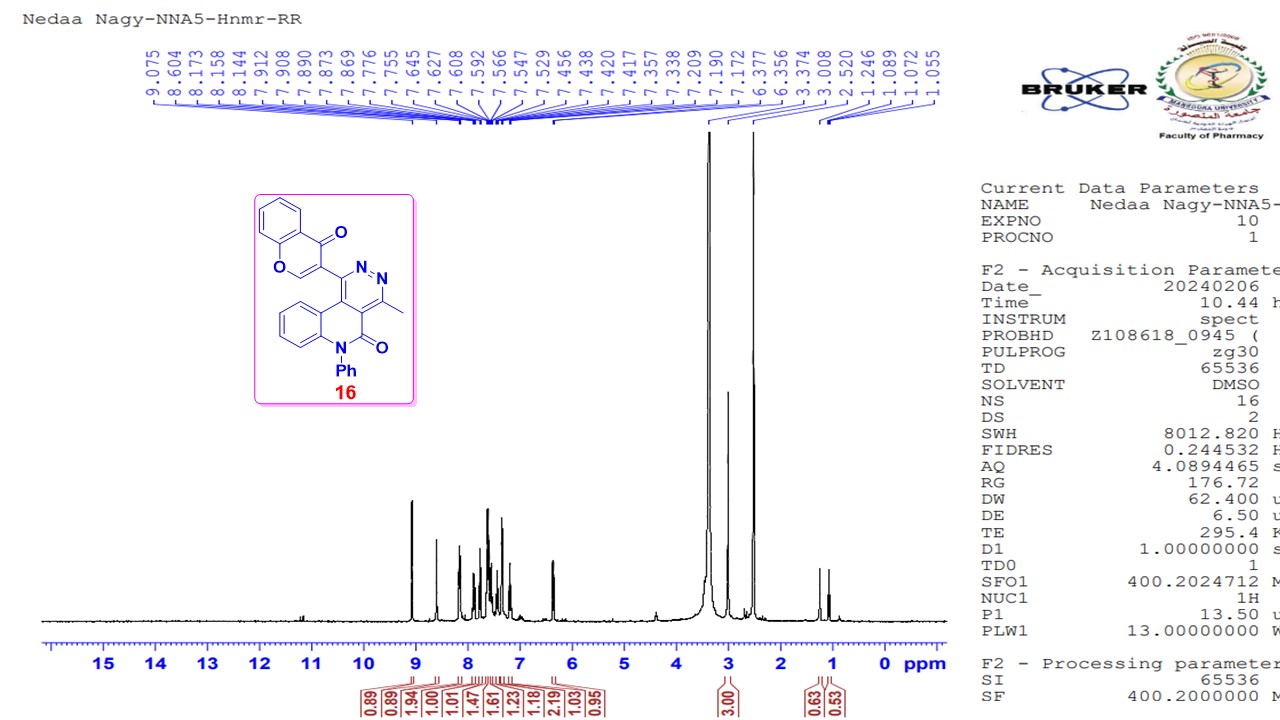
Figure 55: ^1^H NMR Spectrum of compound 16**

**
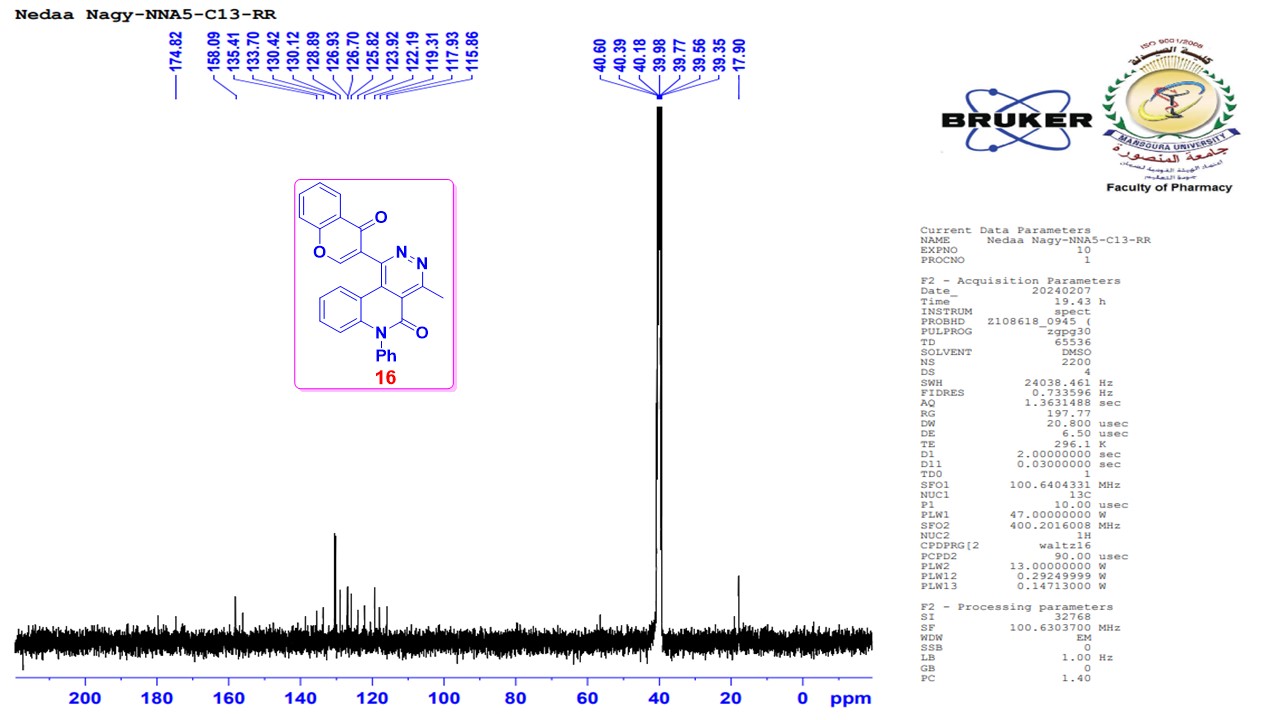
Figure 56: ^13^C NMR Spectrum of compound 16**

**
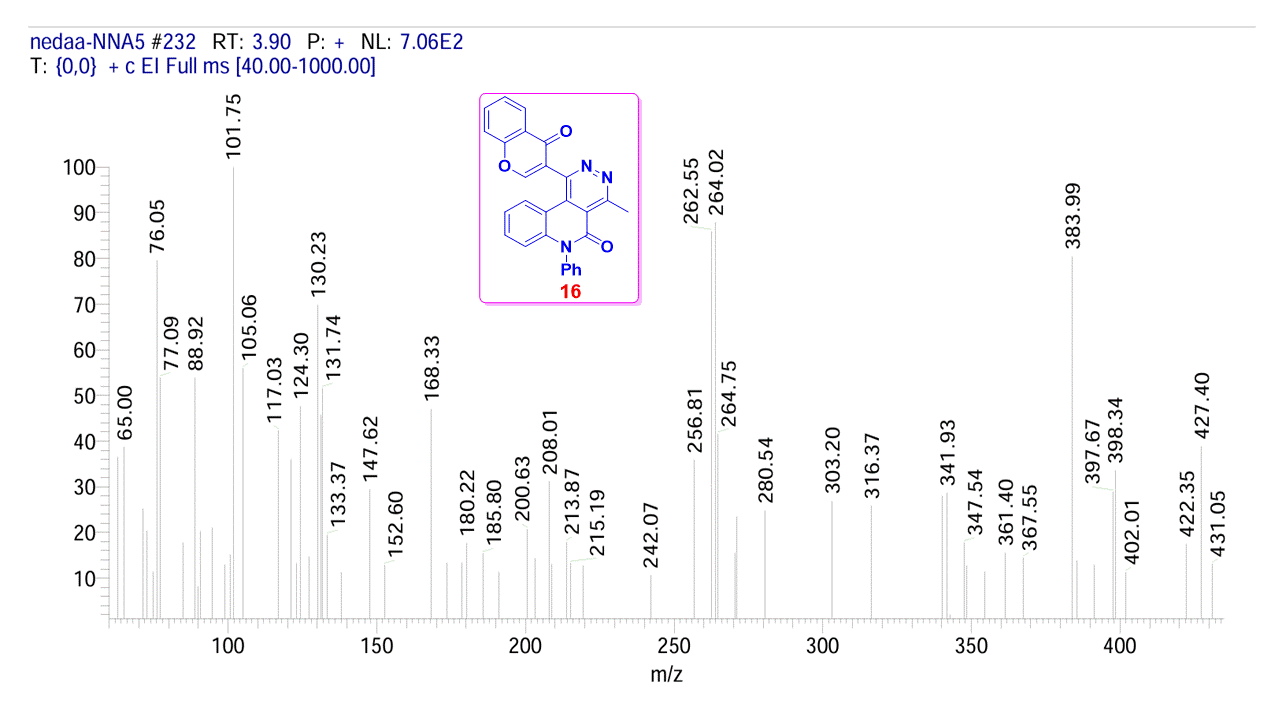
Figure 57: Mass spectrometry of compound 16**

**
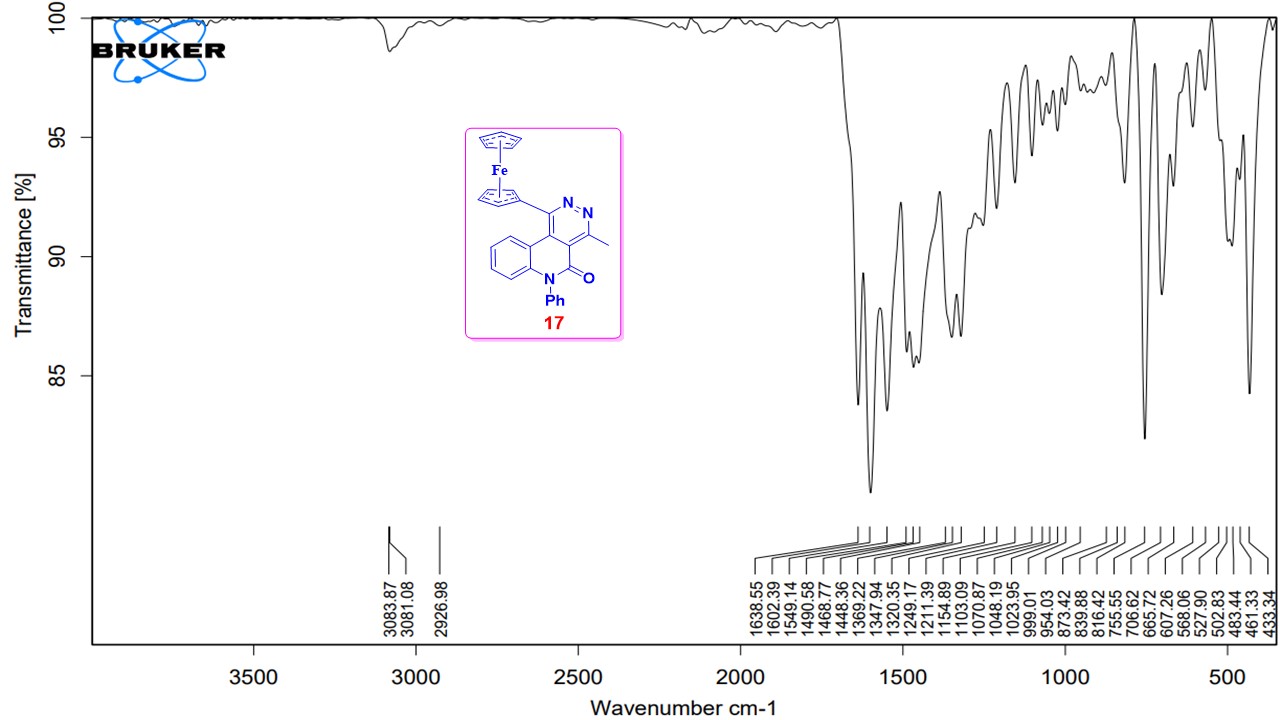
Figure 58: IR Spectrum of compound 17**

**
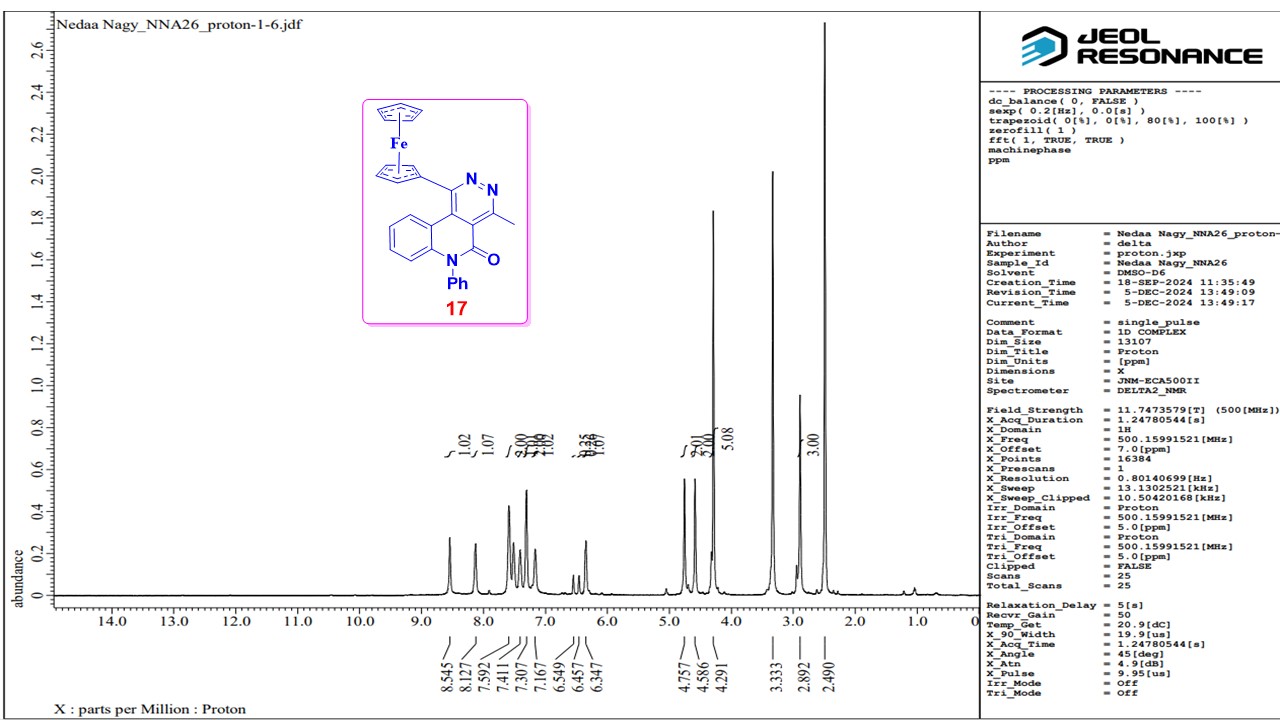
Figure 59: ^1^H NMR Spectrum of compound 17**

**
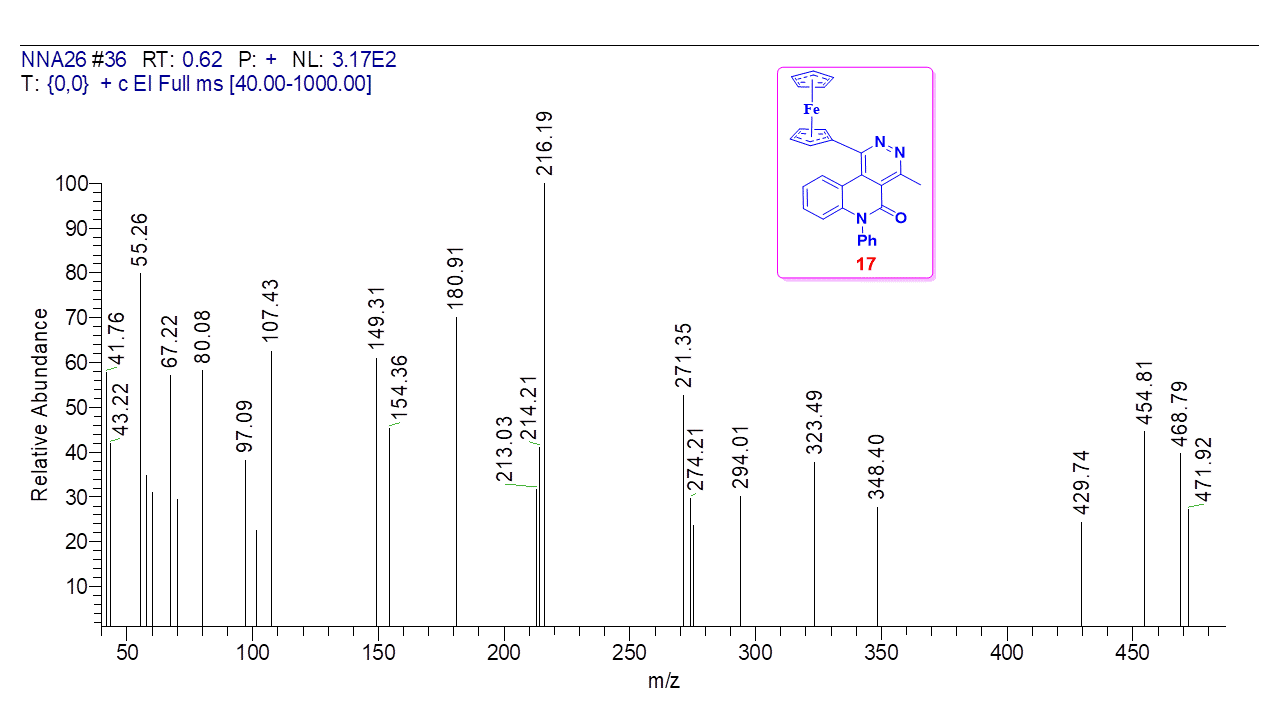
Figure 60: Mass spectrometry of compound 17**

**
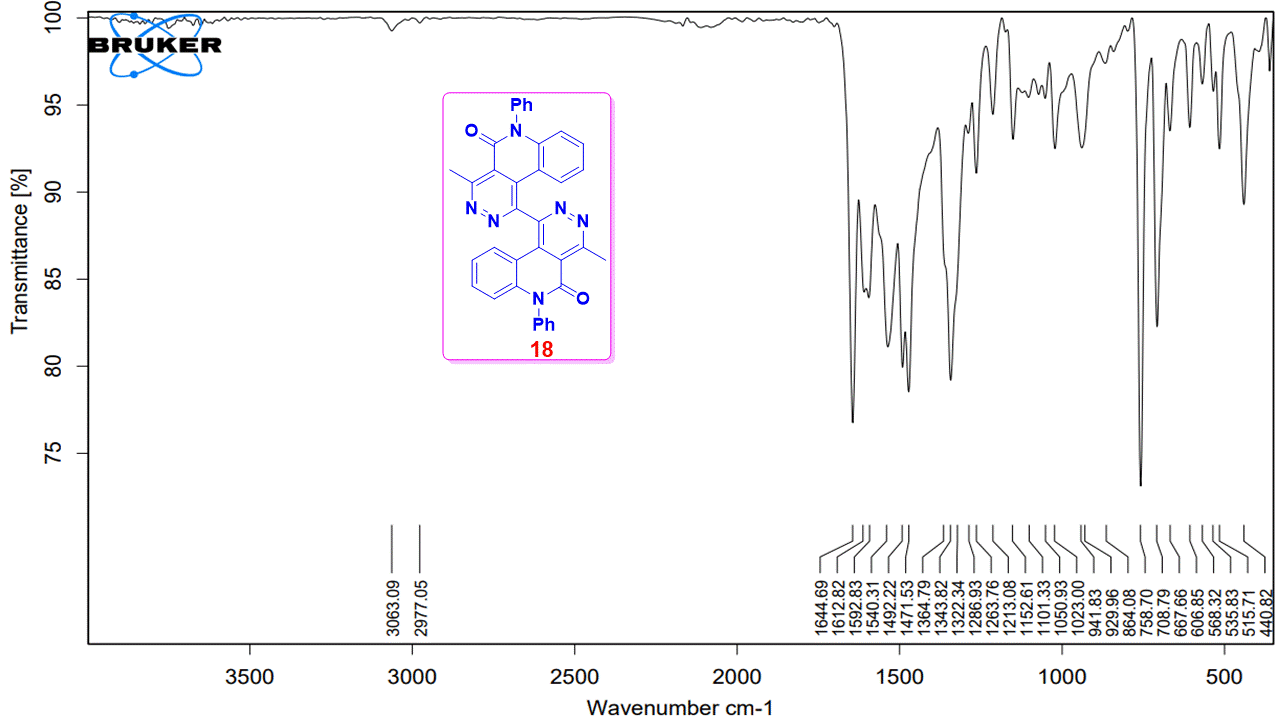
Figure 61: IR Spectrum of compound 18**

**
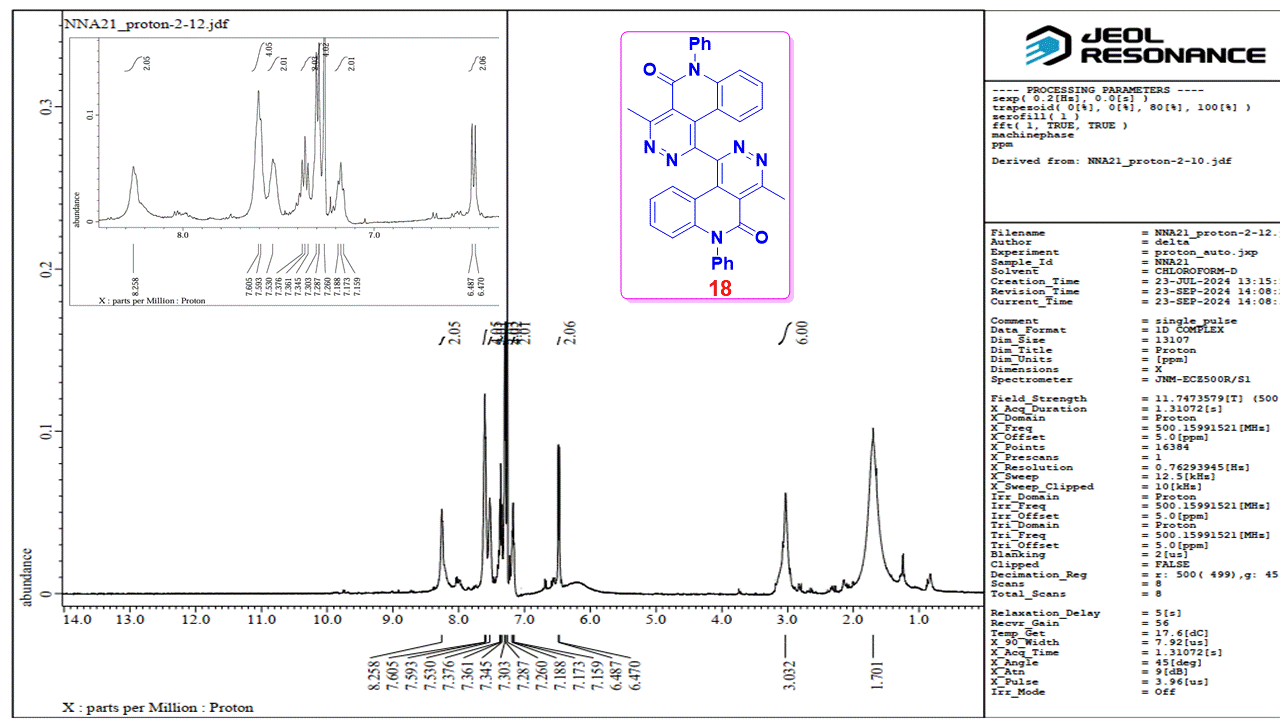
Figure 62: ^1^H NMR Spectrum of compound 18**

**
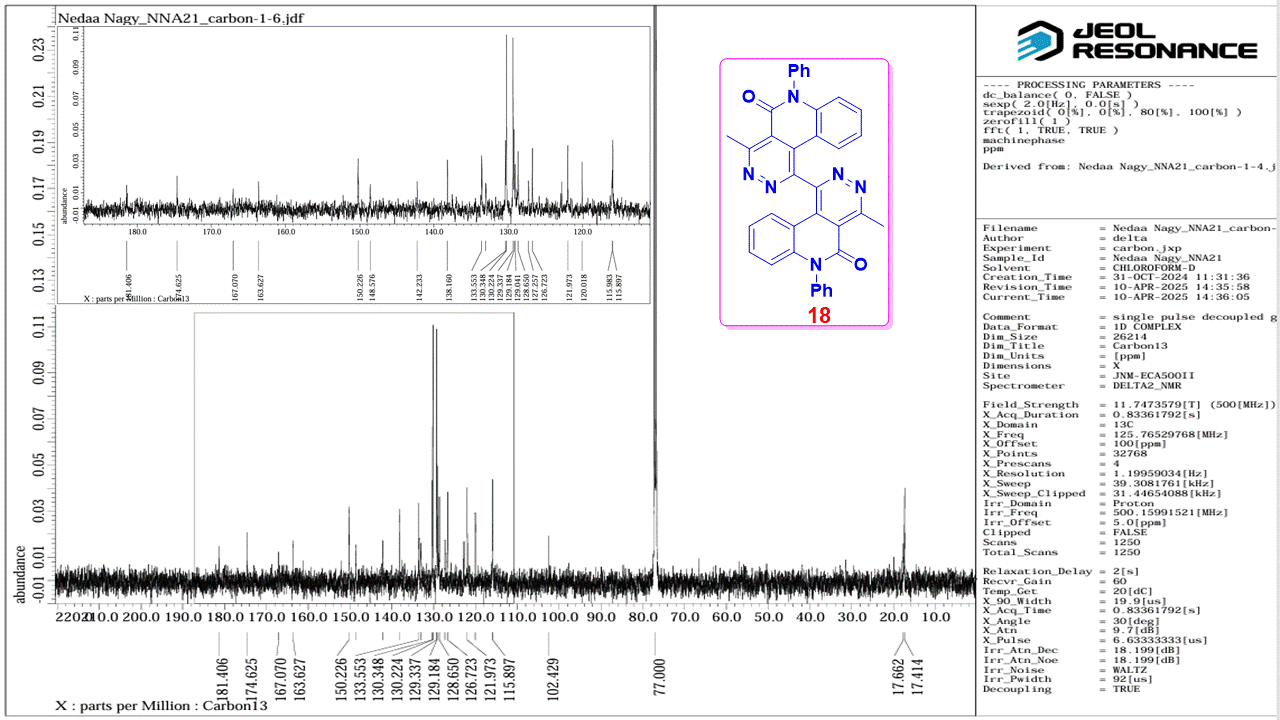
Figure 63: ^13^C NMR Spectrum of compound 18**

**
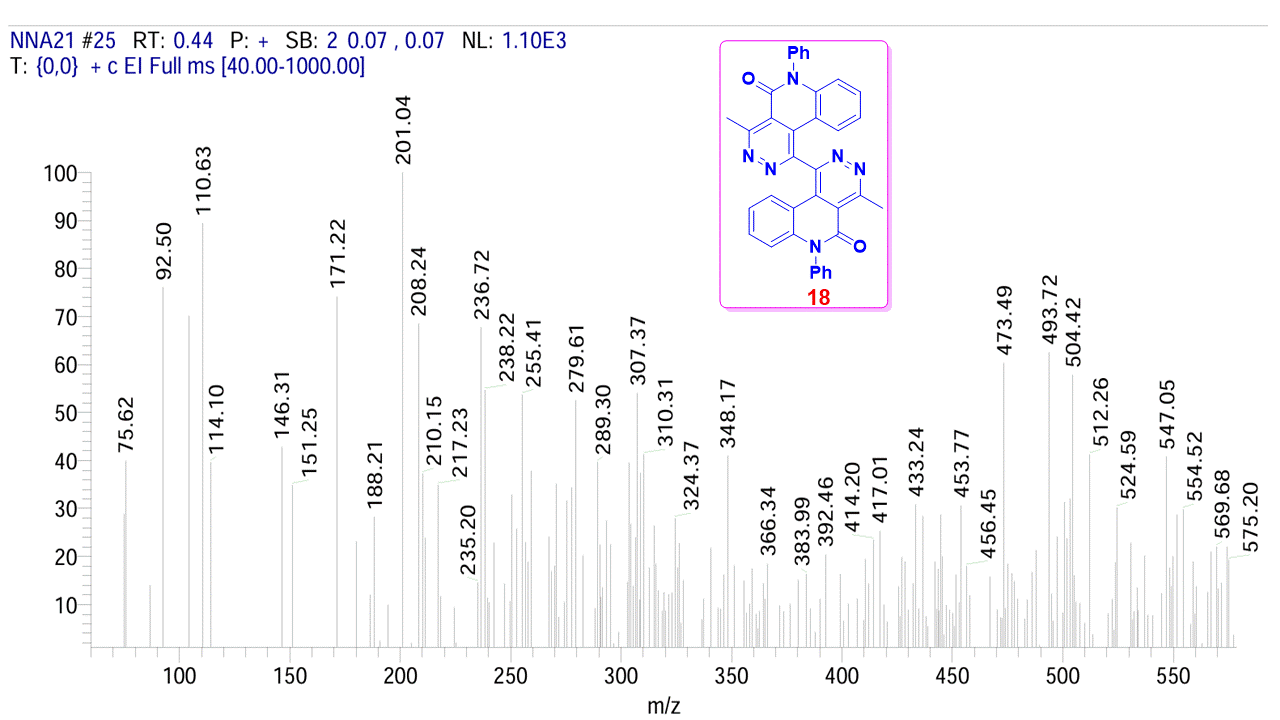
Figure 64: Mass spectrometry of compound 18**

**
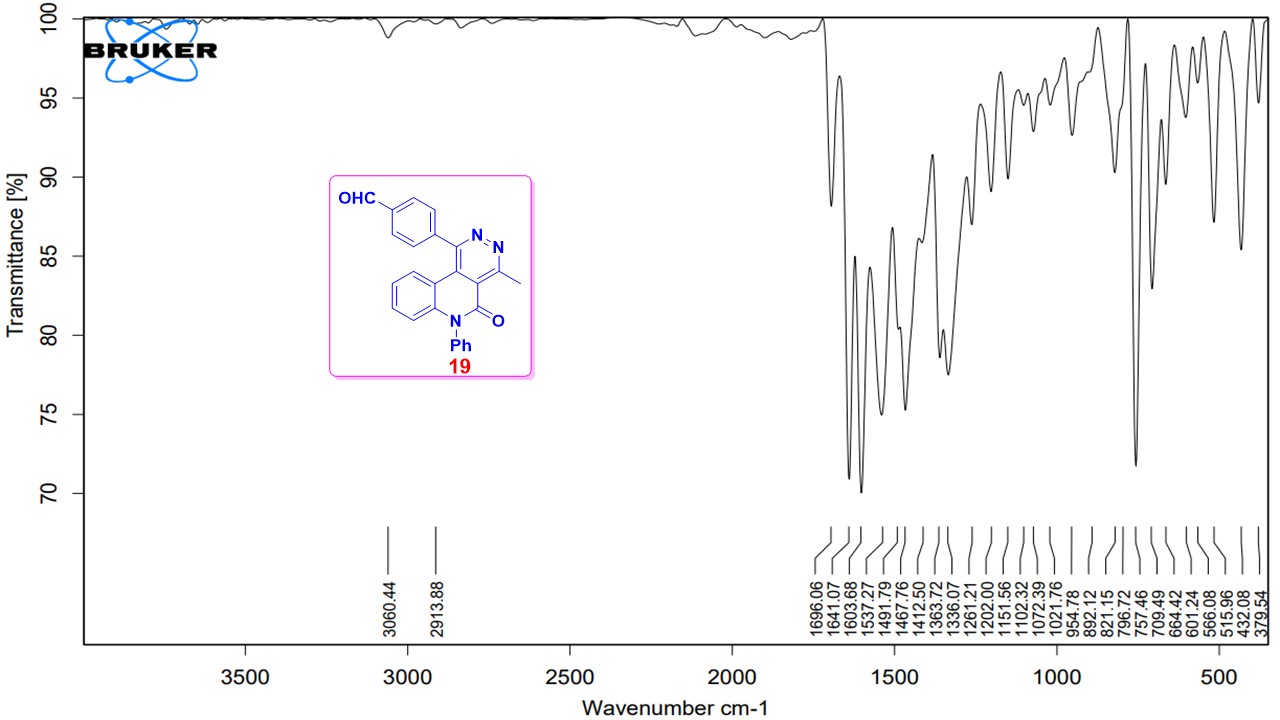
Figure 65: IR Spectrum of compound 19**

**
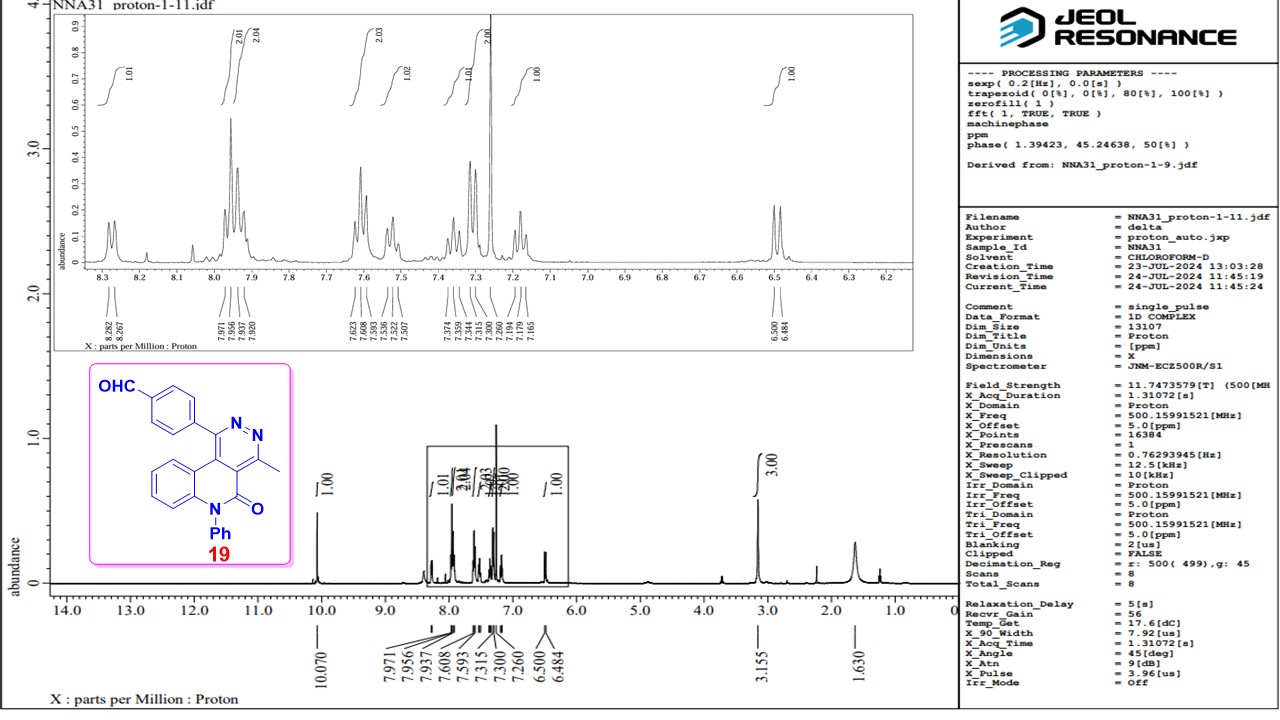
Figure 66: ^1^H NMR Spectrum of compound 19**

**
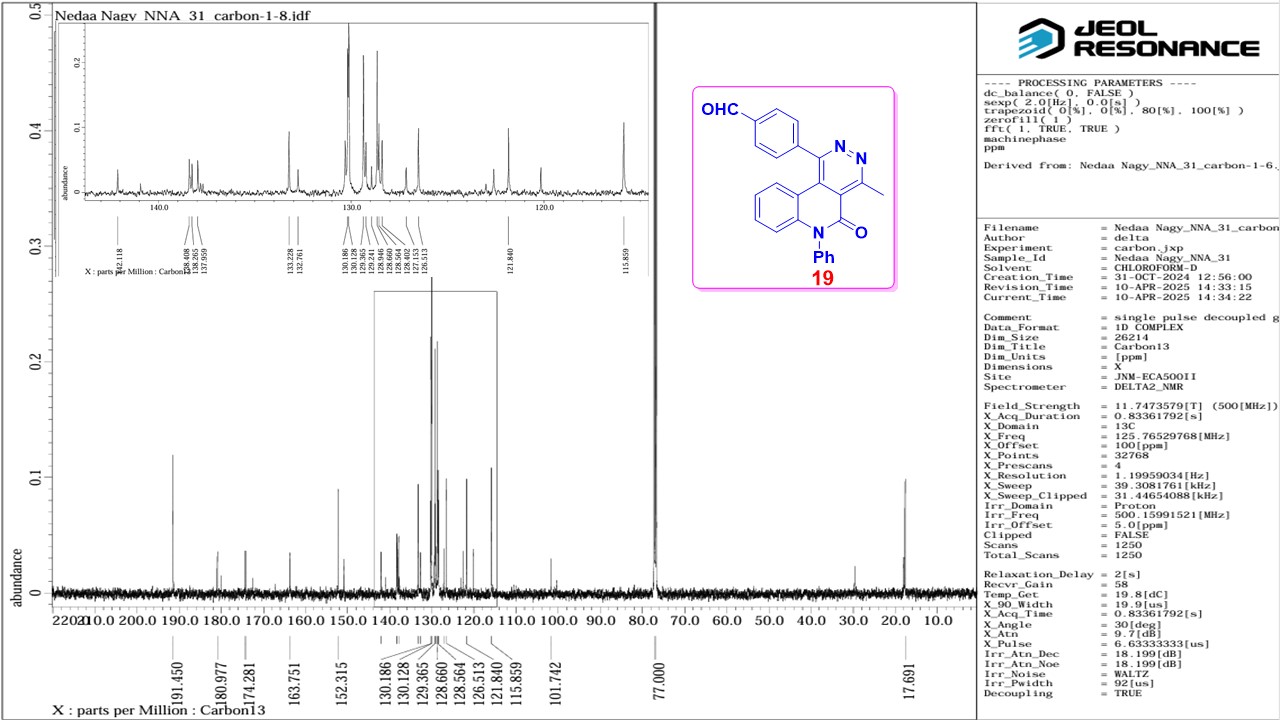
Figure 67: ^13^C NMR Spectrum of compound 19**

**
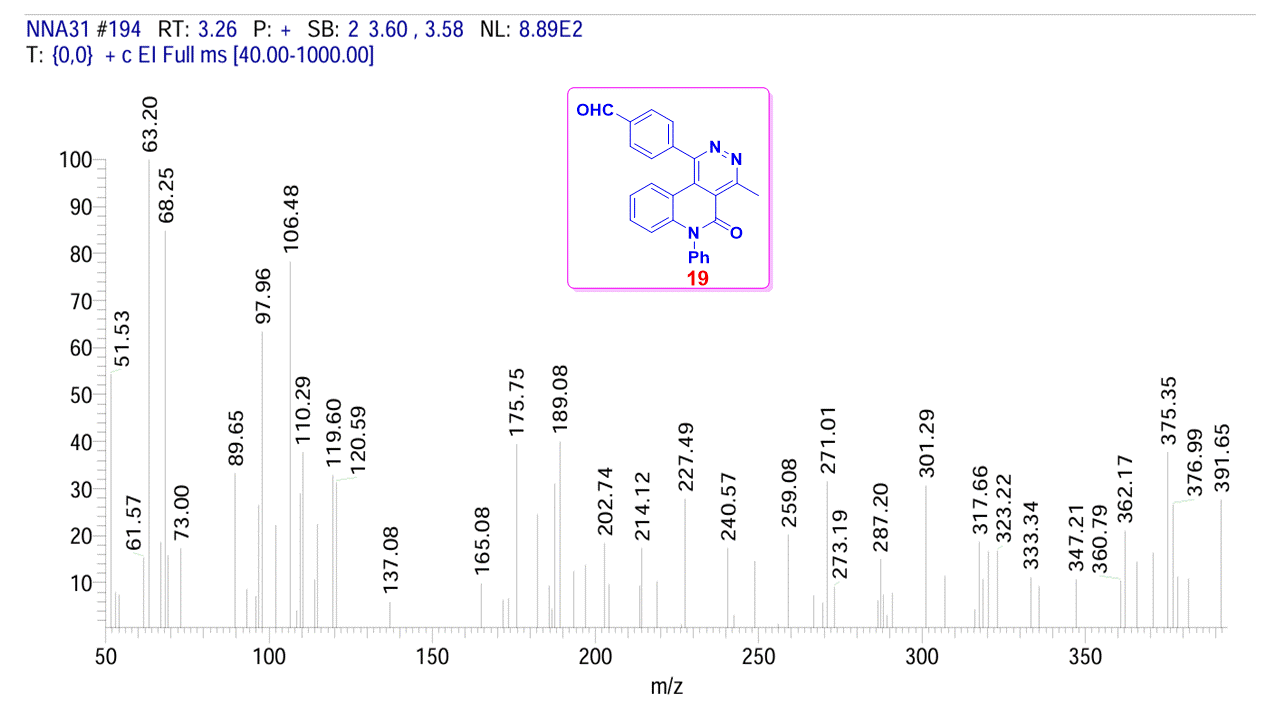
Figure 68: Mass spectrometry of compound 19**

**
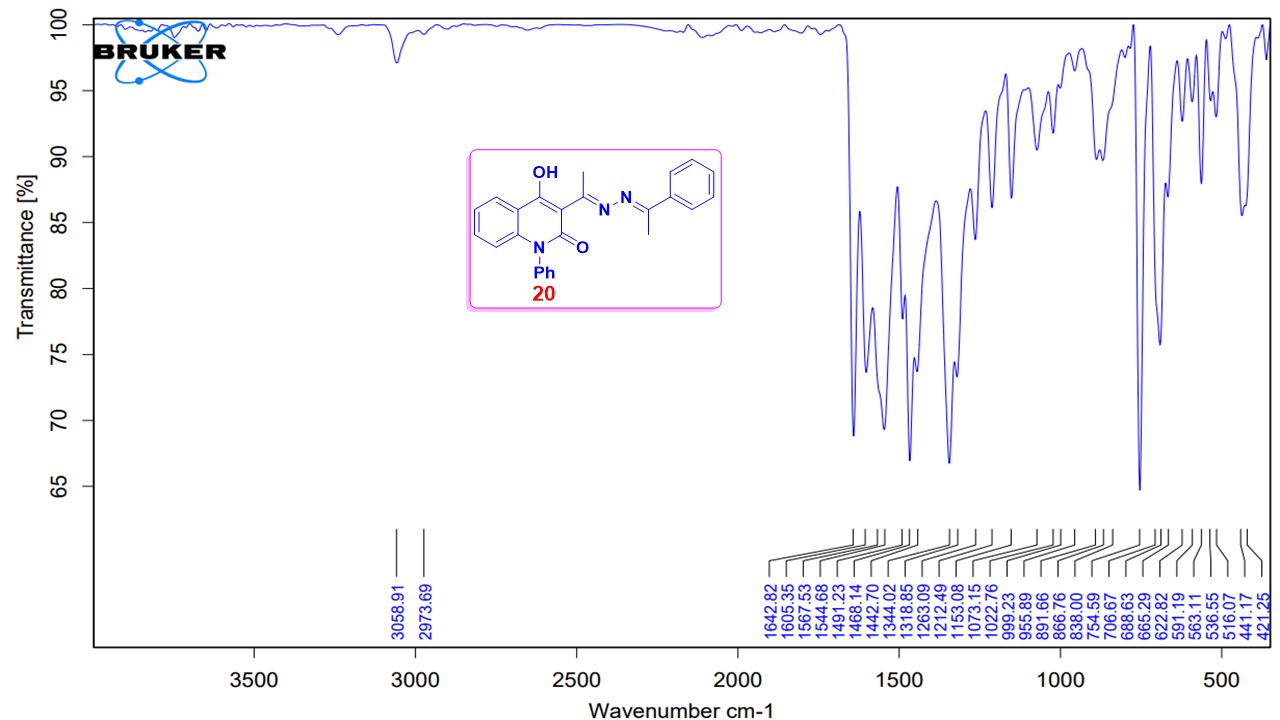
Figure 69: IR Spectrum of compound 20**

**
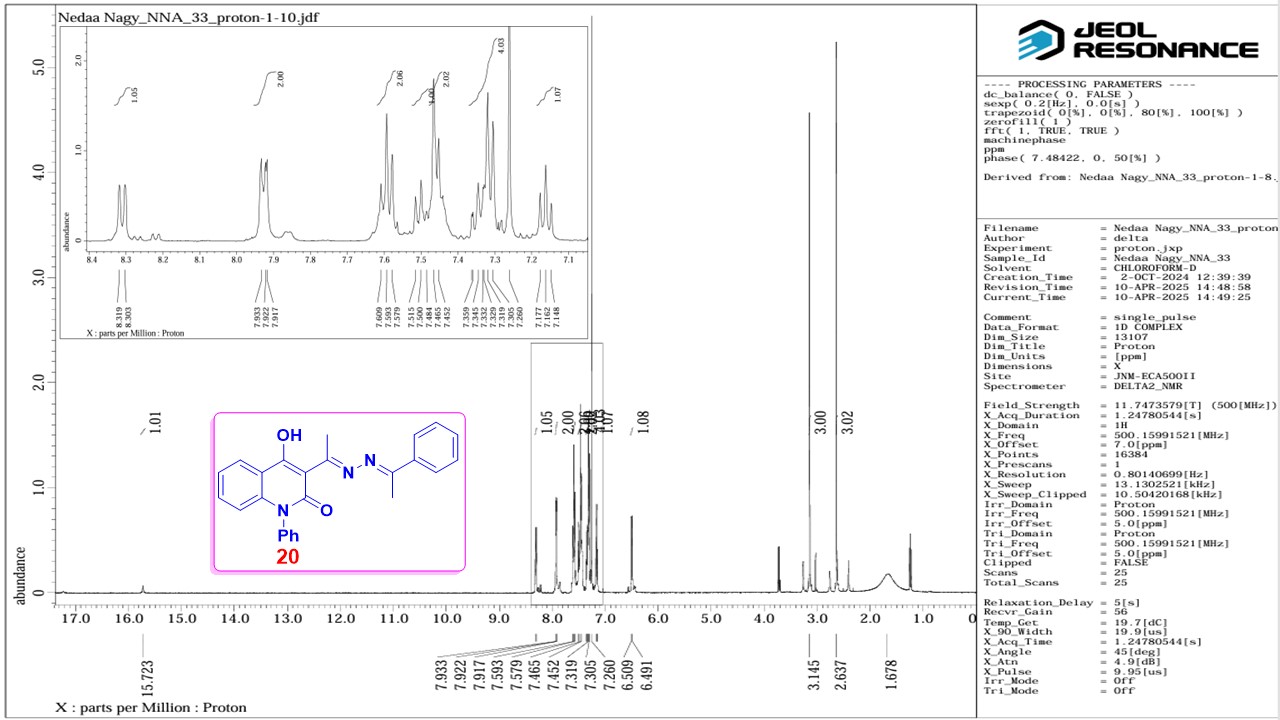
Figure 70: ^1^H NMR Spectrum of compound 20**

**
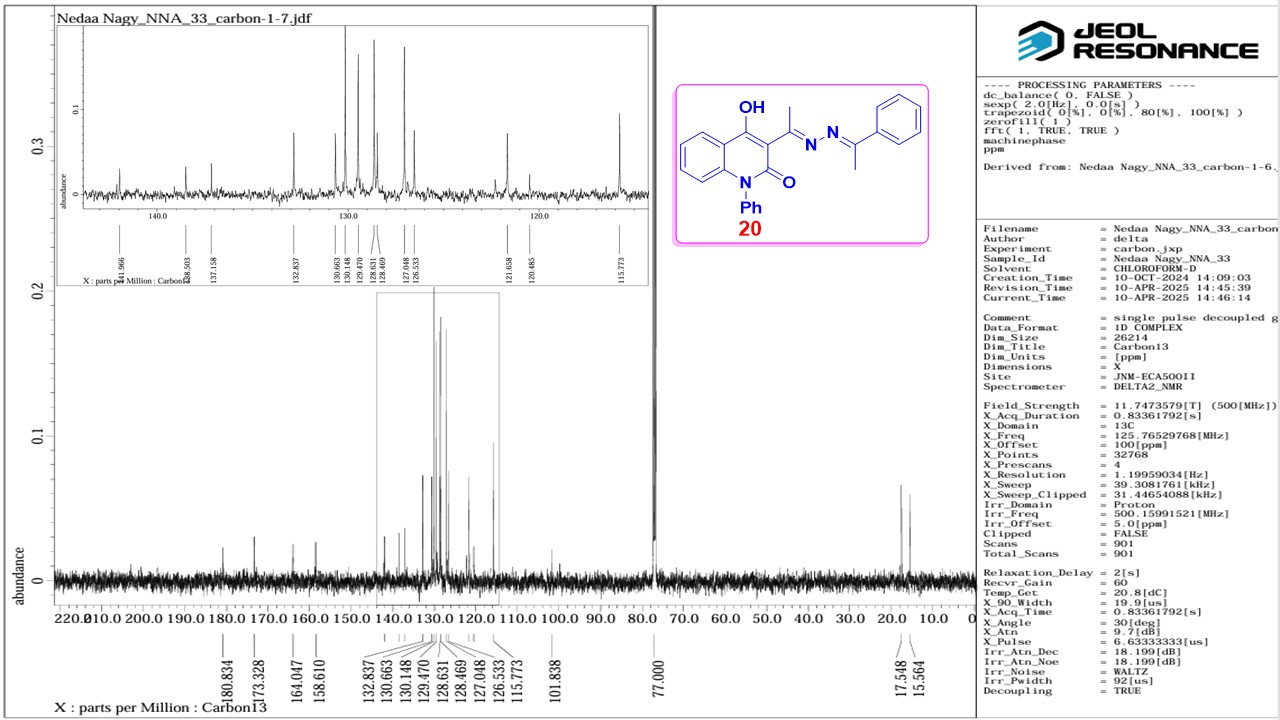
Figure 71: ^13^C NMR Spectrum of compound 20**

**
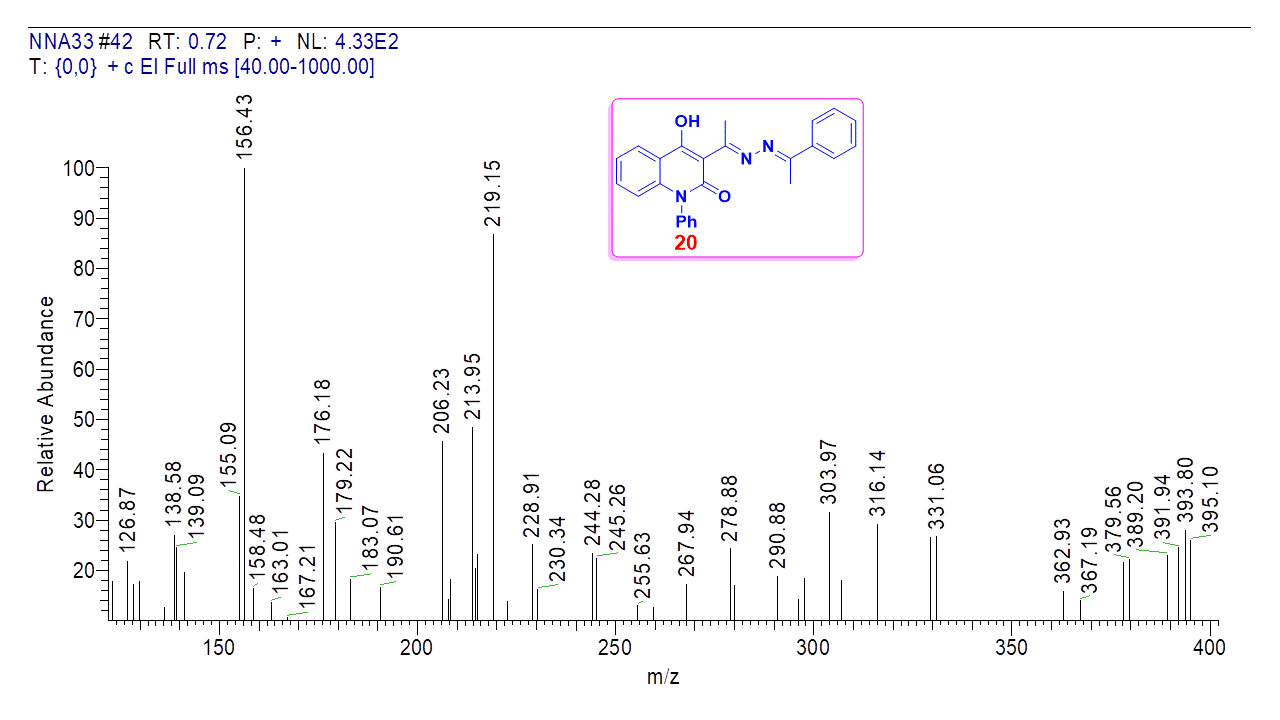
Figure 72: Mass spectrometry of compound 20**

**
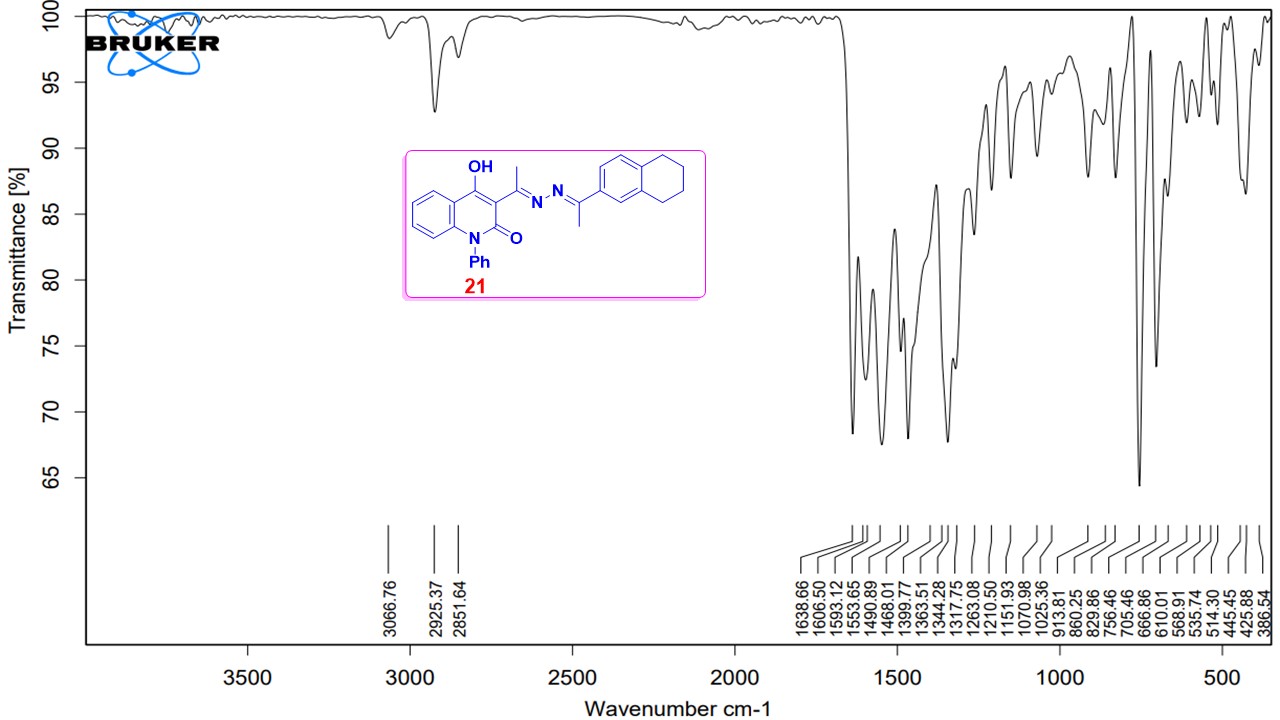
Figure 73: IR Spectrum of compound 21**

**
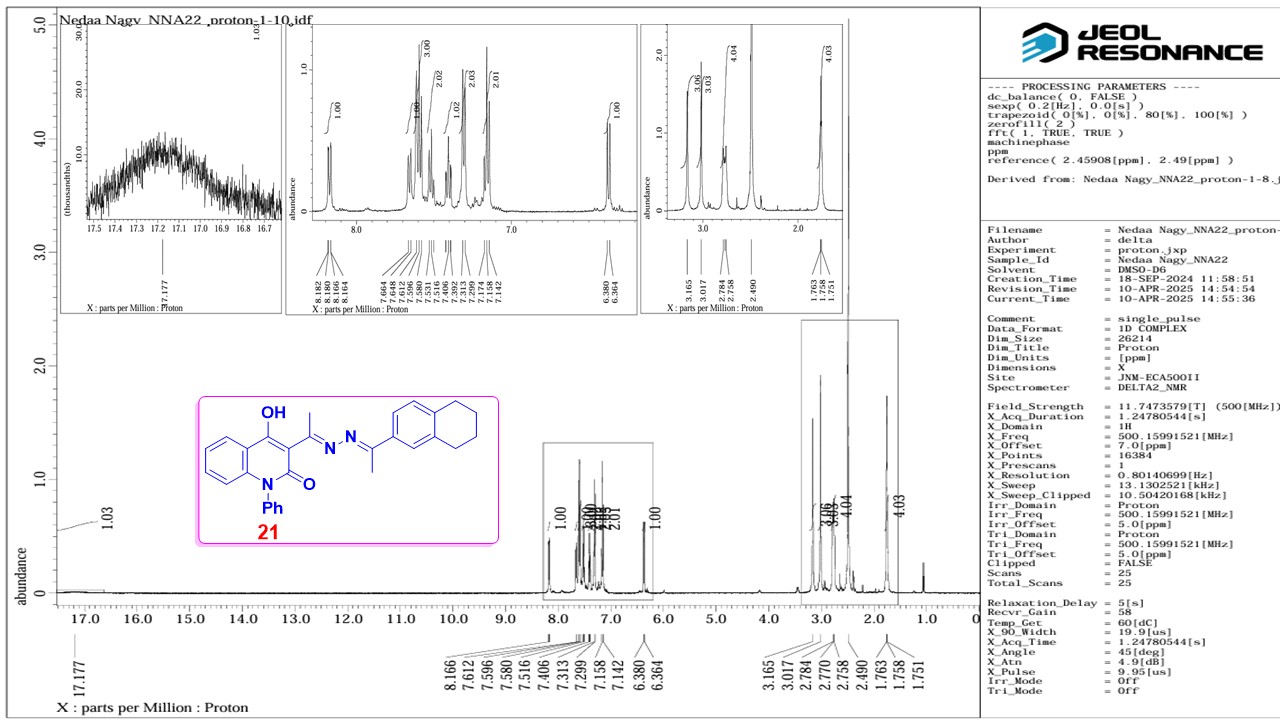
Figure 74: ^1^H NMR Spectrum of compound 21**

**
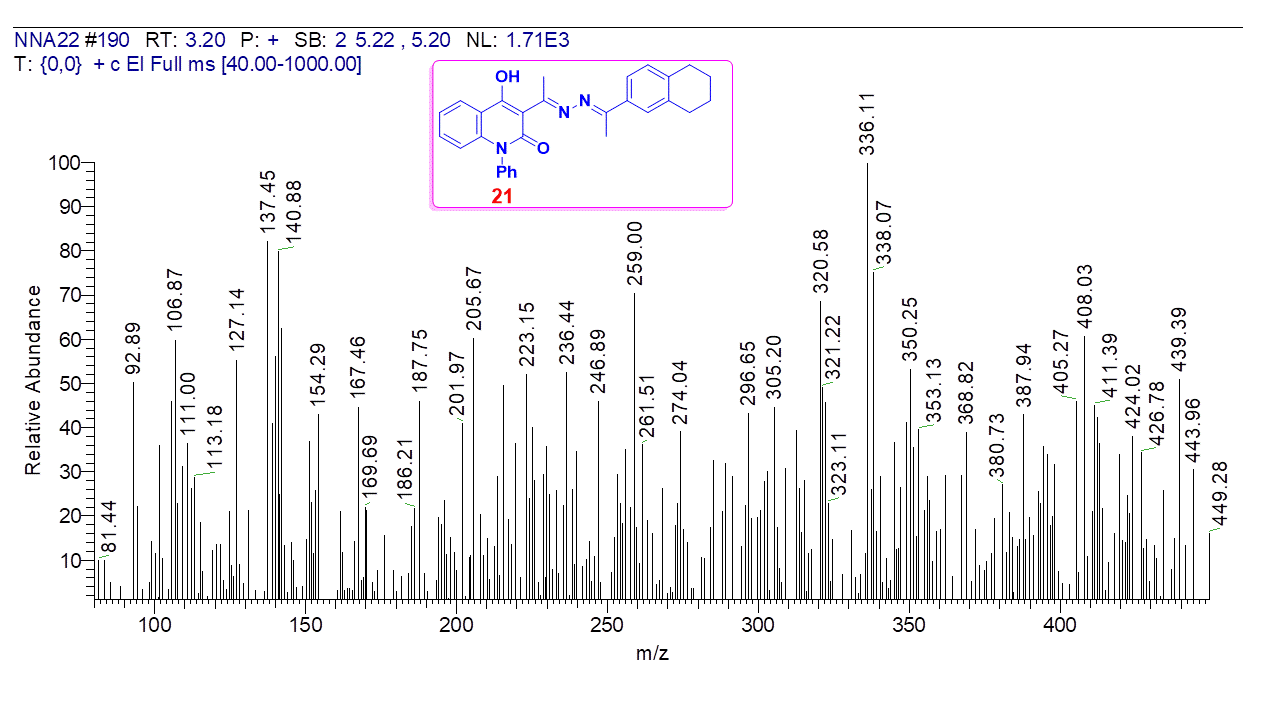
Figure 75: Mass spectrometry of compound 21**

**
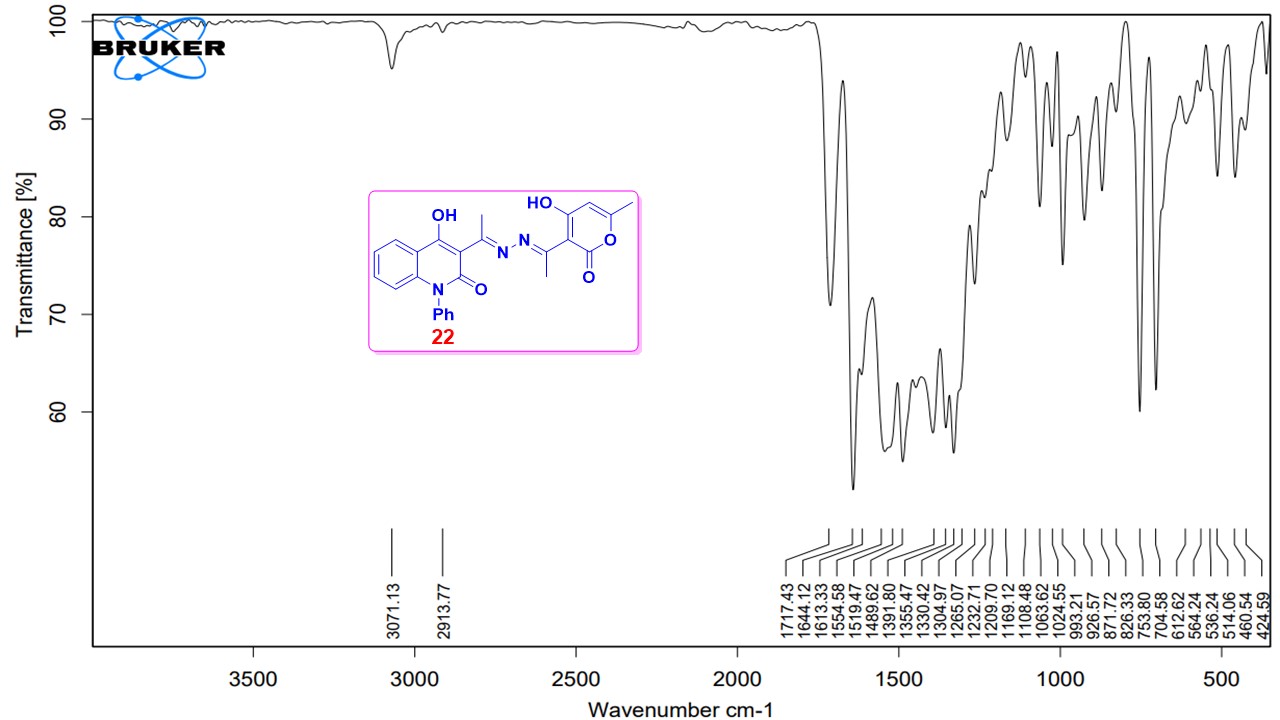
Figure 76: IR Spectrum of compound 22**

**
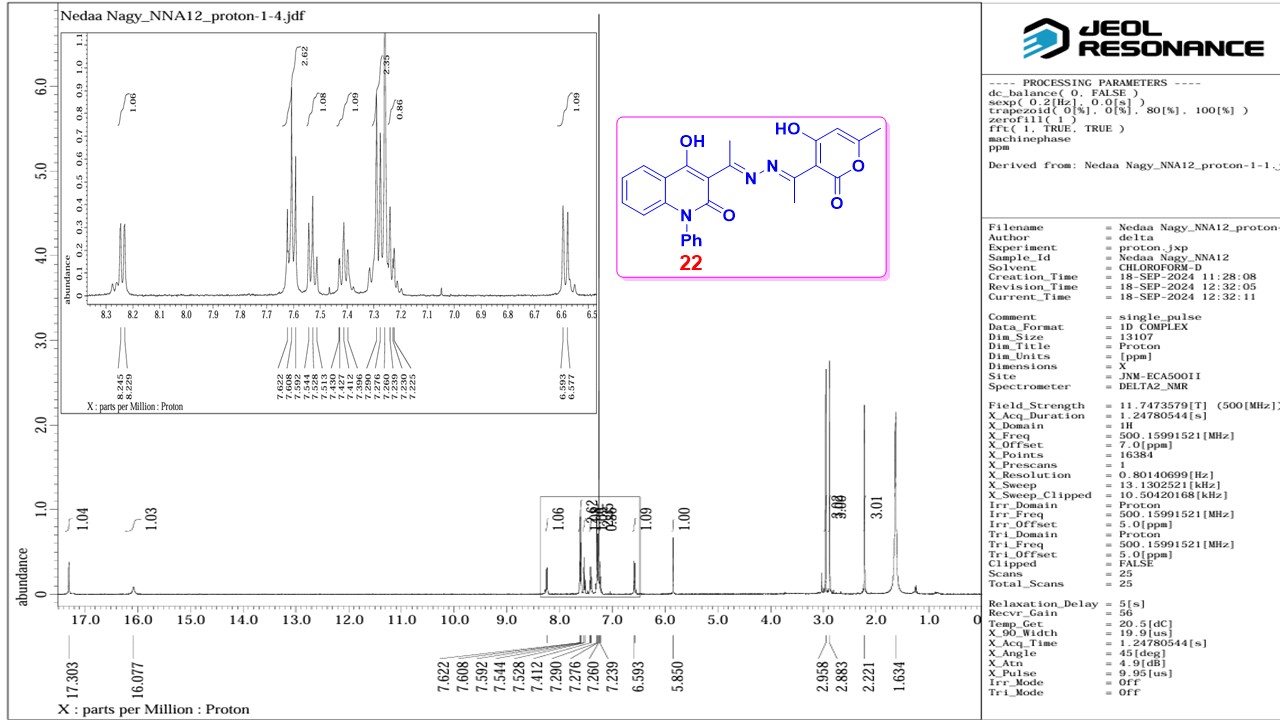
Figure 77: ^1^H NMR Spectrum of compound 22**

**
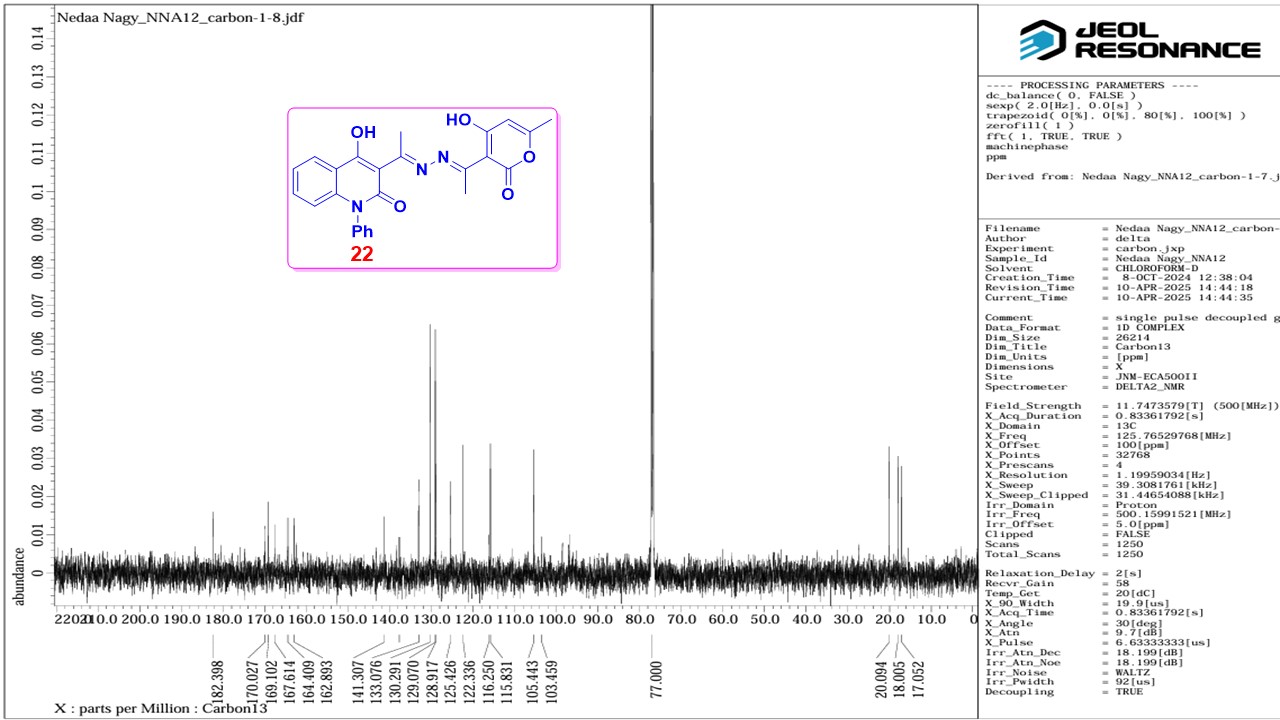
Figure 78: ^13^C NMR Spectrum of compound 22**

**
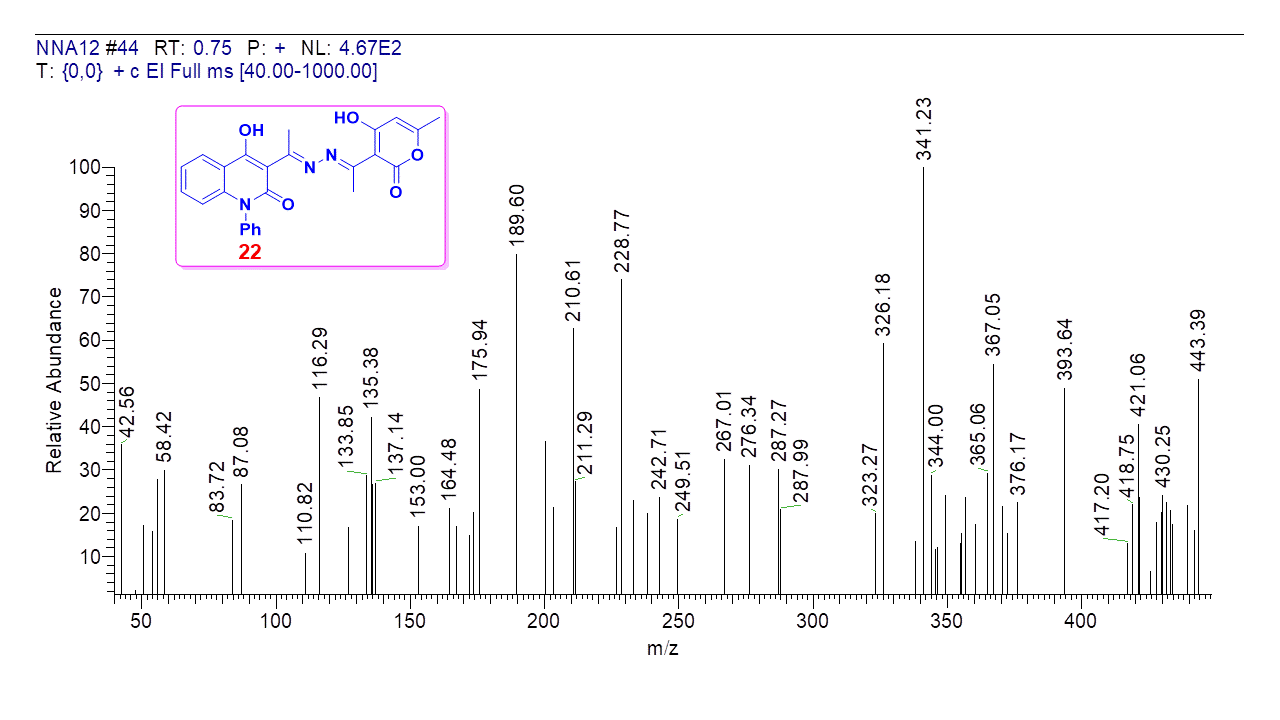
Figure 79: Mass spectrometry of compound 22**

**3.2. Biological evaluation**

**3.2.1. *In vitro* cytotoxicity assay on A549 cell line**

**3.2.1.1. Determination of sample cytotoxicity on cells (MTT protocol)**

All the synthesized compounds were screened for their effect on cell viability against A549 cell line which was obtained from VACSERA, Cairo, Egypt. Whereas, the cells were maintained in DMEM with L-glutamine, supplemented with 10% heat-inactivated FBS and 1% Penicillin/Streptomycin. Then, cells were incubated in humidified conditions at 95% air, 5% CO2, and 37°C). The biological evaluation was screened at Bioscience Research Laboratory, Cairo, Egypt ([bioscience.lase@gmail.com](mailto:bioscience.lase@gmail.com)). Also, it was evaluated using 3-(4,5-dimethylthiazol-2-yl)-2,5-diphenyltetrazolium bromide (MTT) proliferation assay. A594 cells were seeded in 96-well plates (1 × 10^5^ cells/ per well) and incubated at 37°C for 24 hours to develop a complete monolayer sheet. Growth medium was decanted from 96 well micro liter plates after confluent sheet of cells were formed, cell monolayer was washed twice with wash media. After that, two-fold dilutions of tested sample were made in RPMI medium with 2% serum (maintenance medium). Following that 0.1 ml of each dilution was tested in different wells leaving 3 wells as control, receiving only maintenance medium. Next, the plate was incubated at 37°C and examined and the cells were checked for any physical signs of toxicity, e.g. partial or complete loss of the monolayer, rounding, shrinkage, or cell granulation. MTT solution was prepared (5mg/ml in PBS) (BIO BASIC CANADA INC) and 20µl MTT solution were added to each well, place on a shaking table, 150 rpm for 5 minutes, to thoroughly mix the MTT into the media, incubate (37^o^C, 5% CO_2_) for 4 hours to allow the MTT to be metabolized, dump off the media, dry plate on paper towels to remove residue if necessary. Next, resuspend formazan (MTT metabolic product) in 200µl DMSO. Place on a shaking table, 150rpm for 5 minutes, to thoroughly mix the formazan into the solvent. Finally, read optical density at 560 nm and subtract background at 620 nm. Optical density should be directly correlated with cell quantity.

**3.2.1.2. Morphological assay**

❖ Large-scale, morphological changes that occur at the cell surface, or in the cytoskeleton, can be followed and related to cell viability.

❖ Damage can be identified by large decreases in volume secondary to losses in protein and intracellular ions of due to altered permeability to sodium or potassium.

❖ Necrotic cells: nuclear swelling, chromatin flocculation, loss of nuclear basophilia

❖ Apoptotic cells: cell shrinkage, nuclear condensation, nuclear fragmentation

**3.2.2. *In-vitro* anti-tubercular activity**

All the synthesized compounds were screened for their potential to inhibit the growth of M.TB H37Rv strain using the microplate Alamar Blue assay (MABA) method at Bioscience Research Laboratory Cairo, Egypt ([bioscience.lase@gmail.com](mailto:bioscience.lase@gmail.com)). This methodology is non-toxic, uses a thermally stable reagent and shows good correlation with proportional and BACTEC radiometric method. Isoniazid was included as a positive drug control. The experiment was carried out in triplicate using a 96-welled plate under the standard conditions. Briefly, 200µl of sterile deionized water was added to all outer perimeter wells of the plate to minimize evaporation of medium in the test wells during incubation. Subsequently, the 96 wells plate received 100 µl of the Middlebrook 7H9 broth and serial dilution of compounds were made directly on plate using a two-fold serial dilution method. Consequently, the concentrations of tested compounds were in the range of 100 to 3.12 µg/ml. The plates were sealed with parafilm and incubated at 37 °C for 5 days. After this time, 25µl of freshly prepared 1:1 mixture of Alamar Blue reagent and 10% tween 80 was added to the plate and incubated for 24 hrs. A blue color in the well was interpreted as no bacterial growth, and pink color was scored as growth. The lowest concentration which prevented the color change from blue to pink and apparently caused complete inhibition of growth of microorganisms was considered as the minimum inhibitory concentration (MIC).

**3.3. Molecular simulations**

Docking simulations were performed using Auto-Dock version 4.2 and MGL 1.5.6 tools for predicting the protein-ligand interactions. The X-ray crystallographic structures of the target enzymes were retrieved from Protein Data Bank (PDB) with ID: 1M17 and 1OUZ. All target structures were loaded to Discovery Studio Visualizer, and water, unwanted ligands, and hydrogen atoms were removed. Co-crystallized ligands of the target proteins were selected to define the binding sites, and attributes of active pocket were noted. The target proteins were saved in pdb format, loaded to Auto-Dock 4.2 for protein preparation by adding polar hydrogen atoms and Kollman charges, and saved in pdbqt format. The ligand molecules were built using Chem Draw (19.2) as mol file. The mol file of all the molecules was energy minimized using Chem 3D. The energy minimization was carried out using Molecular Mechanics (MM2) and MOPAC module. The energy minimized ligand was saved in pdb format in order to be recognized by the Auto-Dock 4.2 software. The optimized structures were loaded to Auto-Dock tools and saved in pdbqt format. Grid dimensions were set manually as per the attributes defined in Discovery Studio Visualizer. Further docking was continued, and binding interactions of each pose was analyzed in Discovery Studio Visualizer. Whereas, the co-crystallized ligands of each protein to contextualize binding affinity and interaction profiles were reported for full compatible comparison (Figure 80).


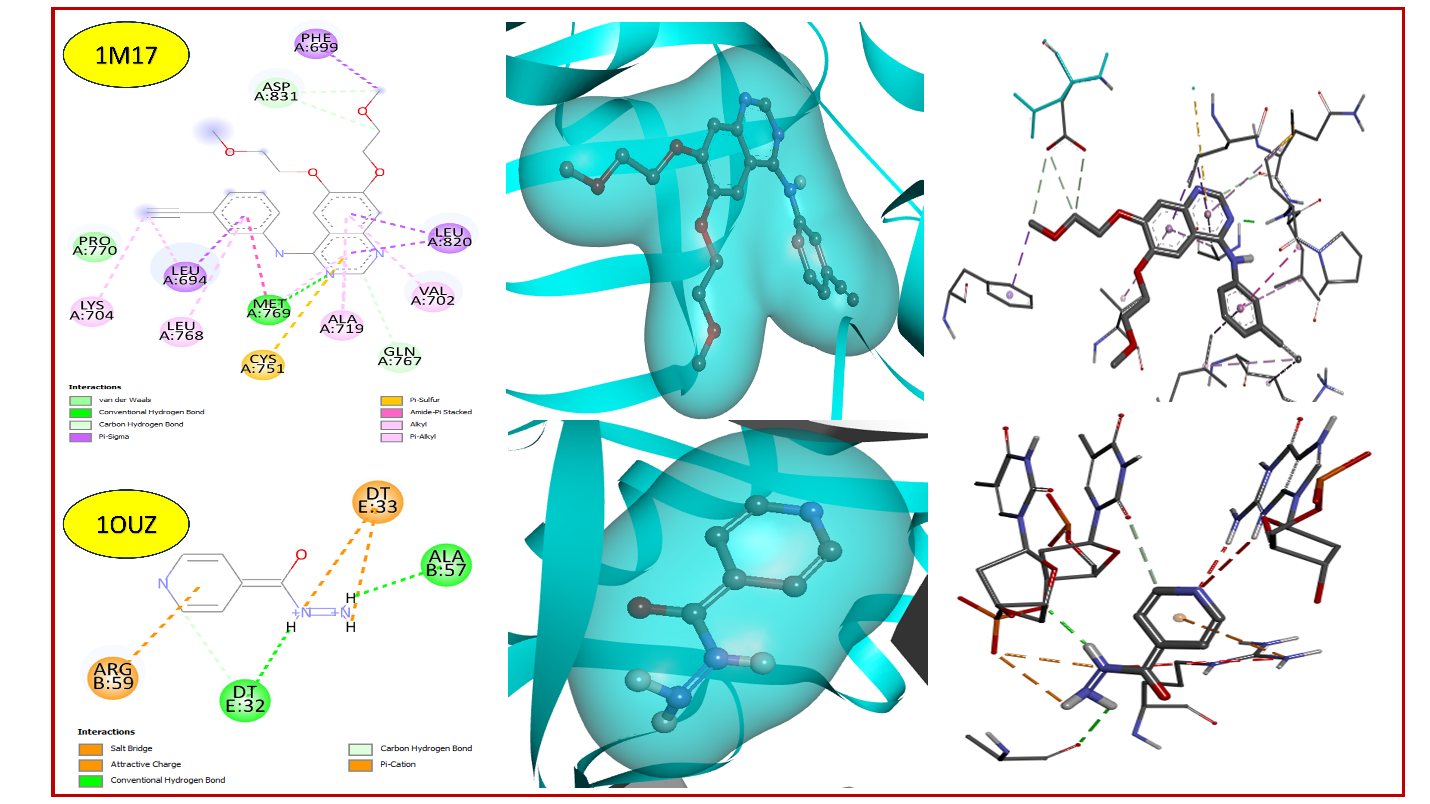


**Figure 80: 2D and 3D interaction plots of the co-crystallized ligands of 1M17 and 1OUZ proteins.**

**3.4.** **Molecular Modeling**

The design of molecularly synthesized compounds can be anticipated through the use of computational chemistry tools, which provide an effective method for analyzing their stability and calculating the total energy of tautomeric or isomeric forms. The Gaussian 09 software package was employed to conduct cluster calculations, utilizing the B3LYP exchange functional within the framework of density functional theory (DFT). A basis set of 6-31G (d to p) was applied to perform the DFT analysis of the synthesized compounds.

**3.5. In silico pharmacokinetics ADME prediction.**

The pharmacokinetic parameters of all substances were predicted using an online web tool, the SwissADME program of the Molecular Modelling Group of the Swiss Institute of Bioinformatics [1], which is freely available at <http://www.swissadme.ch>.The software calculated several pharmacokinetic parameters and descriptors, including the octanol/water partitioning coefficient, aqueous solubility, brain/blood permeability, and HIA capacity. The percentage of oral absorption (%ABS) was estimated using the approach of Zhao et al13 using the following equation:

*ABS*% = 109 – (0.345 * *TPSA*)

where TPSA was topological polar surface area.

**References**

[1] Daina A, Michielin O, Zoete V. SwissADME: a free web tool to evaluate pharmacokinetics, drug-likeness and medicinal chemistry friendliness of small molecules. *Sci Rep*. 2017; 7:42717.
